# Elucidation of Xenobiotic Metabolism Pathways in Human Skin and Human Skin Models by Proteomic Profiling

**DOI:** 10.1371/journal.pone.0041721

**Published:** 2012-07-26

**Authors:** Sven van Eijl, Zheying Zhu, John Cupitt, Magdalena Gierula, Christine Götz, Ellen Fritsche, Robert J. Edwards

**Affiliations:** 1 Centre for Pharmacology and Therapeutics, Division of Experimental Medicine, Imperial College London, London, United Kingdom; 2 Leibniz Institut für Umweltmedizinische Forschung, Heinrich-Heine-Universität, Düsseldorf, Germany; 3 Department of Dermatology and Allergology, University Clinic RWTH, Aachen, Germany; University Paris Diderot-Paris 7, France

## Abstract

**Background:**

Human skin has the capacity to metabolise foreign chemicals (xenobiotics), but knowledge of the various enzymes involved is incomplete. A broad-based unbiased proteomics approach was used to describe the profile of xenobiotic metabolising enzymes present in human skin and hence indicate principal routes of metabolism of xenobiotic compounds. Several *in vitro* models of human skin have been developed for the purpose of safety assessment of chemicals. The suitability of these epidermal models for studies involving biotransformation was assessed by comparing their profiles of xenobiotic metabolising enzymes with those of human skin.

**Methodology/Principal Findings:**

Label-free proteomic analysis of whole human skin (10 donors) was applied and analysed using custom-built PROTSIFT software. The results showed the presence of enzymes with a capacity for the metabolism of alcohols through dehydrogenation, aldehydes through dehydrogenation and oxidation, amines through oxidation, carbonyls through reduction, epoxides and carboxylesters through hydrolysis and, of many compounds, by conjugation to glutathione. Whereas protein levels of these enzymes in skin were mostly just 4–10 fold lower than those in liver and sufficient to support metabolism, the levels of cytochrome P450 enzymes were at least 300-fold lower indicating they play no significant role. Four epidermal models of human skin had profiles very similar to one another and these overlapped substantially with that of whole skin.

**Conclusions/Significance:**

The proteomics profiling approach was successful in producing a comprehensive analysis of the biotransformation characteristics of whole human skin and various *in vitro* skin models. The results show that skin contains a range of defined enzymes capable of metabolising different classes of chemicals. The degree of similarity of the profiles of the *in vitro* models indicates their suitability for epidermal toxicity testing. Overall, these results provide a rational basis for explaining the fate of xenobiotics in skin and will aid chemical safety testing programmes.

## Introduction

Human skin is important as a passive physical barrier that protects the body from the deleterious effects of noxious chemicals, but it also protects the body in a more active fashion, through enzymes that are present inside the skin. These xenobiotic metabolising enzymes (XMEs) are located mostly in keratinocytes within the epidermis, although levels are lower than those found in liver [1], [2]. Accurate detection and characterisation of the low levels of skin XMEs has proved difficult and as a consequence comprehensive information that describes the xenobiotic-metabolising capacity of skin is lacking [3].

Animals are widely used for the testing of new chemical entities, and until recently these have included cosmetic ingredients that have been tested for skin irritation, corrosion and genotoxicity. However, under the 7th Amendment to the EU Cosmetics Directive, which came into force in March 2009, animal testing of cosmetic ingredients has been discontinued. As a result of this there is a pressing need to develop ethically acceptable alternative model systems that can be used to assess the safety of such chemicals [4]. To this end, a number of artificial human skin models have been developed for application as toxicity tests to replace those that previously made use of animals for this purpose. These include EpiDerm, a three-dimensional multilayered skin culture derived from human neonatal foreskin keratinocytes, Episkin, a reconstructed human epidermis model derived from female adult keratinocytes from mammoplasty, and RHE, an *in vitro* reconstructed human epidermis, consisting of normal human keratinocytes derived from human neonatal foreskin. These three-dimensional models reproduce many of the characteristics of normal human epidermis [5]. In addition, HaCaT cells, a spontaneously immortalized male human keratinocyte cell line [6], have been widely used for skin cytotoxicity and sensitization testing [7], [8]. Ideally, biotransformation of xenobiotics by these *in vitro* models should mimic closely that which occurs *in vivo*, although at present knowledge of the enzymes involved is far from complete.

In this study, we set out to determine the XME profiles of whole human skin (comprising both epidermis and dermis) and four *in vitro* epidermal skin models of that are currently being used for toxicity testing. The aim was to characterise the major pathways of biotransformation present and to determine the consistency of the XME profiles between the models.

## Results

### XMEs expression in whole human skin

Ten whole skin samples and 5 liver samples were analysed for the presence of XMEs. Proteomic analysis was performed separately on both microsomal and cytosol fractions and the data combined. Overall, the proteomic analysis indicated the presence of >2000 proteins in both skin and liver. PROTSIFT was used to analyse these data and showed that whole skin contained 36 XMEs (Table 1). Almost all of these proteins were also found in liver. The comparative levels in skin were mostly 4- 10 fold lower than liver, although some enzymes were detected more readily in skin (Table 1). Skin XMEs encompassed those with a variety of functional processes including oxidoreduction, hydrolase, transferase and antioxidant. Many of these oxidoreduction enzymes are responsible for the metabolism of alcohols, aldehydes and ketones. The two hydrolase enzymes were detected in all skin samples analysed. As for the transferases, several isoforms of glutathione S-transferase (GST) were identified in skin. Apart from GST pi which was ∼2-fold higher than in liver, the levels were 2–8 fold lower than those in liver. GST activity was measured in skin using 1-chloro-2,4-dinitrobenzene as a substrate and showed clearly detected levels (91±42 nmol/mg/min, n = 10), which were ∼8-fold higher in liver (753±134 nmol/mg/min, n = 5), and consistent with the relative levels of the GST alpha, mu and omega isoforms. Similarly, enzymes that function as antioxidants were detected in the majority of skin samples analysed and present at levels similar or slightly lower to those of liver.

**Table 1:** XMEs detected in whole skin. Protein identification was based on the presence of ≥2 different tryptic peptides in at least two donors.

			Detection rate (% of samples)		Relative level	
Protein	NCBI number	Fraction	Skin	Liver	skin/liver	p-value
OXIDOREDUCTASE						
3-hydroxyacyl-CoA dehydrogenase type-2	NP_004484.1, NP_001032900.1	cytosol	80	100	0.10	<0.001
alcohol dehydrogenase 1B	NP_000659.2	cytosol	100	100	0.25	<0.001
alcohol dehydrogenase 4	NP_000661.2	cytosol	30	100	0.23	<0.001
alcohol dehydrogenase class-3	NP_000662.3	cytosol	30	20	0.67	0.31
aldehyde dehydrogenase 1A1	NP_000680.2	cytosol	70	100	0.13	<0.001
aldehyde dehydrogenase 1L1	NP_036322.2	cytosol	20	100	0.08	<0.001
aldehyde dehydrogenase 2	NP_000681.2	microsome	100	100	0.07	<0.001
aldehyde dehydrogenase 3A2	NP_001026976.1, NP_000373.1	microsome	40	100	0.07	<0.001
aldehyde dehydrogenase 9A1	NP_000687.3	cytosol	50	100	0.39	<0.001
aldehyde oxidase	NP_001150.3	cytosol	30	100	0.26	<0.001
aldo-keto reductase 1A1	NP_697021.1, NP_006057.1	cytosol	100	100	0.06	<0.001
aldo-keto reductase 1C	NP_001809.2, NP_995317.1, NP_001128713.1, NP_001345.1, NP_001344.2, NP_003730.4	cytosol	100	100	0.04	<0.001
amine oxidase [flavin-containing] B	NP_000889.3	microsome	80	100	0.03	<0.001
carbonyl reductase [NADPH] 1	NP_001748.1	cytosol	100	100	0.11	<0.001
carbonyl reductase [NADPH] 3	NP_001227.1	cytosol	70	100	0.17	<0.001
membrane primary amine oxidase	NP_003725.1	microsome	90	0	>6.7	<0.001
NADH-ubiquinone oxidoreductase	NP_004997.4	microsome	100	100	1.11	0.51
prostacyclin synthase	NP_000952.1	microsome	90	0	>1.7	<0.001
short-chain dehydrogenase/reductase 7	NP_057113.1, NP_001099041.1, NP_056325.2	microsome	70	100	0.21	<0.05
HYDROLYASE						
epoxide hydrolase 1	NP_001129490.1, NP_000111.1	microsome	100	100	0.10	<0.001
liver carboxylesterase 1	NP_057113.1, NP_001099041.1, NP_056325.2	microsome	100	100	0.62	0.21
TRANSFERASE						
gamma-glutamyltransferase 5	NP_001093252.1, NP_004112.2, NP_001093251.1	microsome	100	40	1.20	0.31
glutathione S-transferase alpha	NP_665683.1, NP_001503.1, NP_714543.1, NP_000837.3, NP_000838.3	cytosol	50	100	0.12	<0.001
glutathione S-transferase mu	NP_671489.1, NP_001135840.1, NP_666533.1, NP_000552.2, NP_000840.2, NP_000842.2, NP_000841.1, NP_000839.1	cytosol	100	100	0.17	<0.001
glutathione S-transferase omega	NP_899062.1, NP_004823.1	cytosol	40	100	0.12	<0.001
glutathione S-transferase pi	NP_000843.1	cytosol	100	100	1.97	<0.05
glutathione S-transferase theta	NP_000845.1, NP_001074312.1, NP_000844.2	cytosol	50	100	0.57	<0.05
ANTIOXIDANT						
catalase	NP_001743.1	cytosol	90	100	0.24	<0.001
glutathione peroxidase 3	NP_002075.2	cytosol	100	0	>1.2	<0.001
peroxiredoxin-1	NP_002565.1, NP_859047.1, NP_859048.1	cytosol	100	100	0.21	<0.001
peroxiredoxin-2	NP_005800.3, NP_859428.1	cytosol	100	100	0.84	0.51
peroxiredoxin-5	NP_857635.1, NP_857634.1, NP_036226.1	cytosol	70	100	0.25	<0.001
peroxiredoxin-6	NP_004896.1	cytosol	100	100	0.05	<0.001
OTHER						
14-3-3 protein beta/alpha	NP_003395.1, NP_647539.1	cytosol	100	100	1.56	<0.05
glyceraldehyde-3-phosphate dehydrogenase	NP_002037.2	cytosol	100	100	0.82	0.51
long chain fatty acid-CoA ligase 1	NP_004449.1, NP_055977.3, NP_001986.2	microsome	60	100	0.03	<0.001

The proteins identified have been classified into functional groups as indicated. The corresponding NCBI numbers are indicated for each protein and for all members of groups of related proteins. The sub-cellular fraction in which each protein was principally detected is shown. The proportion of donor samples (skin n = 10, liver n = 5) in which each protein was identified is indicated. Fold difference was calculated by summing the intensity values of all detected peptides for a protein and comparing the values obtained for skin and liver. Where no peptides were detected, an intensity value equivalent to the limit of detection was used. Statistical significance was assessed using the Mann-Whitney U test.

**Figure 1:**
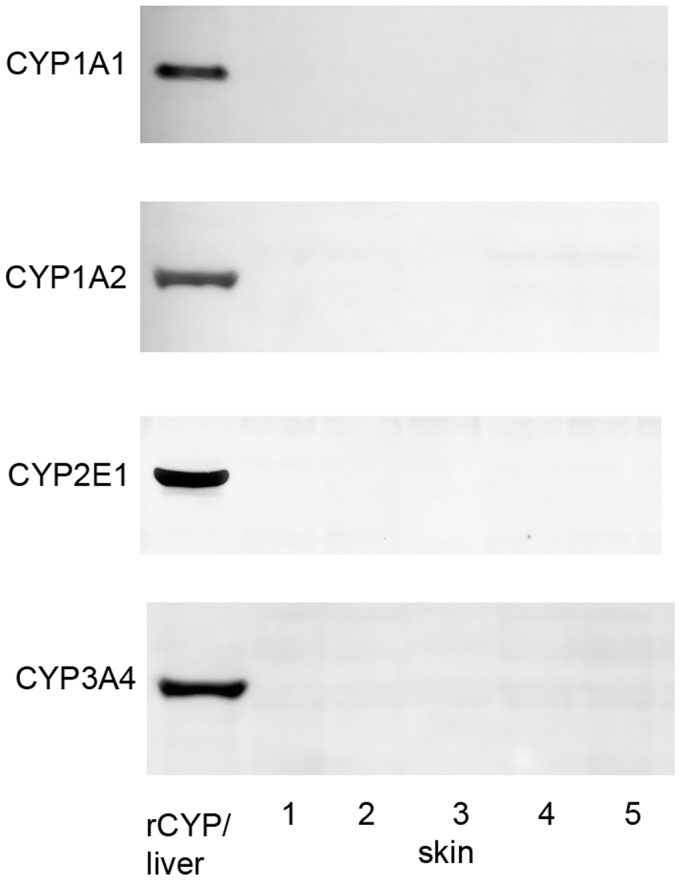
Analysis of CYP expression in skin by immunoblotting. Samples of human whole skin microsomal fraction (75 µg) prepared from 5 donors were separated by SDS-PAGE, transferred to nitrocellulose filters and the presence of CYP1A1, CYP1A2, CYP2E1 and CYP3A4 detected using antibodies specific to each form. The lane on the left hand side contained either 25 µg lymphoblast cell microsomes containing recombinant human CYP1A1 (∼2 pmol), or a sample of human liver loaded with 25, 35 or 5 µg microsomal fraction for detection of CYP1A2, CYP2E1, or CYP3A4, respectively. Immunoreactive bands were developed using goat anti-rabbit-horseradish peroxidase and ECL detection.

The proteomics analysis also showed that liver contained an additional 46 XMEs that were not detected in skin (Table 2). This includes 13 CYP proteins encompassing all of the major forms involved in xenobiotic metabolism. CYP1A2, CYP2E1 and CYP3A4 could not be detected in skin under immunoblotting conditions that readily showed their presence in human liver and CYP1A1 could not be detected in either skin or liver, although a preparation containing recombinant CYP1A1 was readily detected (Figure 1). The limit of detection of these CYP proteins by immunoblotting is 2.5 pmol/mg microsomal protein (Figure S1). Application of increased amounts of the respective antibodies used for detection or increasing the development time only produced non-specific bands without improving the detection of CYPs. The protein loading of 75 µg used was the maximum amount possible without causing problems with the electrophoretic separation of the proteins. Further attempts were also made to detect CYP2B6, CYP2C8, CYP2C9, CYP2C19, CYP2D6 and CYP3A5 in skin samples using appropriate antibodies [9], but no specific immunoreactive bands were detected (data not shown). Similarly, no immunoreactive bands corresponding to any of these CYPs were detected in any of the *in vitro* models tested. The low levels of CYP proteins in skin was consistent with measurement of ethoxyresorufin O-deethylase activity, which was <0.1 pmol/min/mg in skin microsomal fraction (below the limit of detection), whereas the activity in human liver microsomal fraction was 39.5±16.1 pmol/min/mg (n = 5).

**Table 2:** XMEs detected in liver but not detected in whole skin. Protein identification was based on the presence of ≥2 different tryptic peptides.

Protein	NCBI number	Fraction	Detection rate (% of samples)
OXIDOREDUCTASE			
alcohol dehydrogenase 1A	NP_000658.1	cytosol	100
alcohol dehydrogenase 1C	NP_000660.1	cytosol	100
alcohol dehydrogenase 6	NP_001095940.1, NP_000663.1	cytosol	100
aldehyde dehydrogenase 7A1	NP_001173.2	cytosol	100
aldehyde dehydrogenase 8A1	NP_739577.1, NP_072090.1	cytosol	100
aldehyde dehydrogenase X	NP_000683.3	cytosol	100
amine oxidase [flavin-containing] A	NP_000231.1	microsome	100
carbonyl reductase 4	NP_116172.2	cytosol	40
cytochrome P450 1A2	NP_000752.2	microsome	100
cytochrome P450 2A6/2A7/2A13	NP_000753.3, NP_000755.2, NP_000757.2, NP_085079.2	microsome	100
cytochrome P450 2B6	NP_000758.1	microsome	80
cytochrome P450 2C19	NP_000760.1	microsome	100
cytochrome P450 2C8	NP_000761.3	microsome	100
cytochrome P450 2C9	NP_000762.2	microsome	100
cytochrome P450 2D6	NP_001020332.1, NP_000097.2	microsome	100
cytochrome P450 2E1	NP_000764.1	microsome	100
cytochrome P450 2J2	NP_000766.2	microsome	40
cytochrome P450 3A4	NP_073731.1, NP_476437.1, NP_476436.1, NP_059488.2	microsome	100
cytochrome P450 3A5	NP_000768.1	microsome	40
cytochrome P450 4A11	NP_000769.2	microsome	100
cytochrome P450 4F12	NP_076433.2	microsome	100
dimethylaniline monooxygenase [N-oxide-forming] 3	NP_001002294.1, NP_008825.4	microsome	100
dimethylaniline monooxygenase [N-oxide-forming] 5	NP_001138301.1, NP_001452.2	microsome	100
electron transfer flavoprotein-ubiquinone oxidoreductase	NP_004444.2	microsome	100
kynurenine 3-monooxygenase	NP_003670.2	microsome	100
methylmalonate-semialdehyde dehydrogenase [acylating]	NP_005580.1	cytosol	100
NADPH-cytochrome P450 reductase	NP_000932.3	microsome	100
succinate-semialdehyde dehydrogenase	NP_733936.1, NP_001071.1	cytosol	100
sulfide:quinone oxidoreductase	NP_067022.1	microsome	100
xanthine dehydrogenase/oxidase	NP_000370.2	cytosol	100
HYDROLYSIS			
carboxylesterase 2	NP_003860.2, NP_932327.1	microsome	100
epoxide hydrolase 2	NP_001970.2	cytosol	100
TRANSFERASE			
bile salt sulfotransferase	NP_003158.2	cytosol	100
glutathione S-transferase kappa	NP_001137151.1, NP_001137153.1, NP_001137152.1, NP_057001.1	cytosol	100
glutathione S-transferase zeta	NP_665877.1, NP_665878.2, NP_001504.2	cytosol	100
histamine N-methyltransferase	NP_001019246.1, NP_001019245.1, NP_008826.1	cytosol	40
microsomal glutathione S-transferase 1	NP_665734.1, NP_665735.1, NP_064696.1, NP_665707.1	microsome	100
nicotinamide N-methyltransferase	NP_006160.1	cytosol	100
sulfotransferase 1A	NP_803880.1, NP_001045.1, NP_803878.1, NP_803880.1, NP_001046.2, NP_003157.1, NP_001017390.1, NP_001017389.1, NP_808220.1, NP_803564.1, NP_803566.1, NP_803565.1	cytosol	100
thiosulfate sulfurtransferase	NP_003303.2	cytosol	100
STEROID METABOLISM			
11-beta-hydroxysteroid dehydrogenase 1	NP_005516.1, NP_861420.1	microsome	100
17-beta-hydroxysteroid dehydrogenase 13	NP_835236.2, NP_001129702.1	microsome	100
17-beta-hydroxysteroid dehydrogenase type 6	NP_003716.2	microsome	100
hydroxysteroid dehydrogenase like 2	NP_115679.2	cytosol	80
OTHERS			
catechol O-methyltransferase	NP_000745.1, NP_009294.1, NP_001128633.1, NP_001128634.1	cytosol	100
gamma-glutamyl carboxylase	NP_001135741.1, NP_000812.2	microsome	80
glutamate-cysteine ligase catalytic subunit	NP_001489.1	cytosol	40

The proteins identified have been classified into functional groups. The corresponding NCBI numbers are indicated for each protein and for all members of groups of related proteins. The sub-cellular fraction in which they were principally detected is shown. The proportion of liver samples (n = 5) in which each protein was identified is indicated.

Detection of CYP proteins in skin was investigated further by estimating the limit of detection of CYP proteins by the proteomic approach employed. Samples of skin microsomal fraction were spiked with a range of concentrations of CYP1A1, CYP1A2, CYP2E1, CYP3A4 and CYP3A5 and following the standard proteomics workflow tryptic peptides corresponding to these CYP proteins were identified and the limits of detection of each CYP determined (Table 3). The values obtained are similar to those determined previously for non-CYP proteins [10]. These values were then used to calculate the limit of detection of each of the CYP proteins in a 75 µg protein sample (the maximum that could be separated by SDS-PAGE). On this basis the minimum amount of CYP that can be detected in a sample by the procedure used was found to be in the range of 0.1–0.2 pmol/mg microsomal protein. Compared with average values measured in the microsomal fraction from a panel of donors it is apparent that the levels in skin are at least 300-fold lower than that of liver (Table 3).

**Table 3:** Detection of CYP proteins in skin and liver microsomal fraction by LC-MS/MS.

	Limit of detection (fmol)	Microsomal level (pmol/mg)		Relative level in skin compared with liver
Protein		Skin	Liver	
CYP1A1	1.5	<0.11	<0.11	n/a
CYP1A2	2.0	<0.16	50	<0.003
CYP2E1	1.5	<0.11	35	<0.003
CYP3A4	3.0	<0.23	105	<0.002
CYP3A5	1.0	<0.08	25	<0.003

Samples of skin microsomal fraction were spiked with a range of quantities of either recombinant CYP1A1 (expressed in lymphoblast cells) or with human liver microsomal fraction that contains known amounts of CYP1A2, CYP2E1, CYP3A4, and CYP3A5. The normal proteomics workflow was followed to identify peptides corresponding to the CYP proteins. Limits of detection based on the use of at least 2 tryptic peptides were established and based on these values the minimum level detectable by this technique was calculated for skin and compared with the mean level measured in liver. From these values the minimum comparative level in skin was calculated. CYP1A1 was not detected in either skin or liver making any comparison redundant (n/a; not applicable).

The proteomic approach also failed to detect any N-acetyltransferase (NAT) isoform in skin or liver. This was despite the ready detection of NAT activity using p-toluidene as substrate. Samples of skin and liver cytosol contained 0.7–3.0 (range of 10 samples) [11] and 0.1–0.6 (range of 5 samples) nmol/min/mg protein activity, respectively. Consequently, samples of skin cytosol were spiked with a series of amounts of recombinant NAT1 and the proteomics analysis performed to determine a limit of detection for this protein. This was found to be 3 pmol/mg cytosolic protein.

### XME expression in skin models

**Figure 2:**
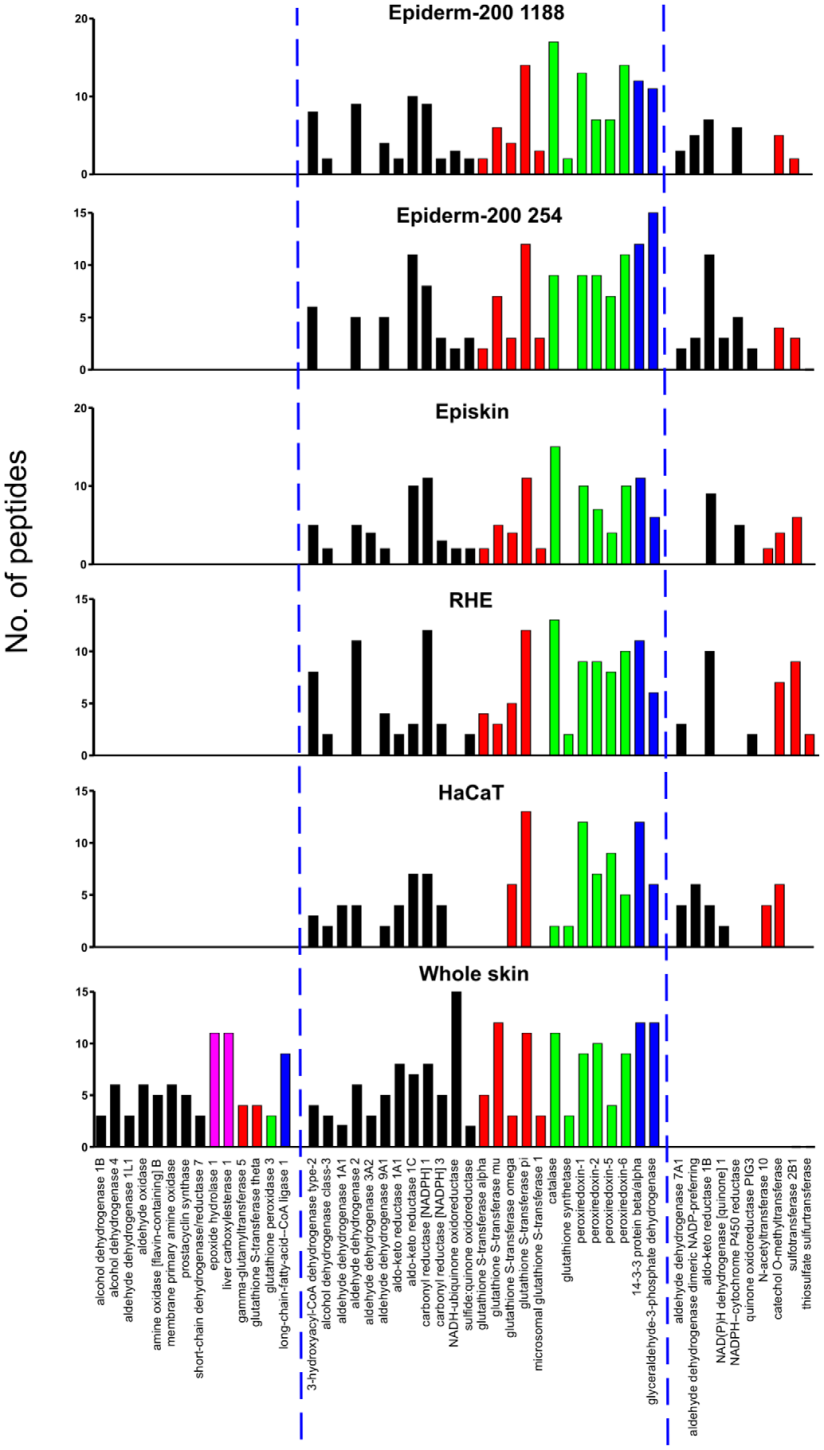
Comparison of XME profiles from *in vitro* skin models and whole skin. The relative amount of each protein is represented by the number of different tryptic peptides specific to each protein or protein family that were detected. Details of the protein accession numbers and their subcellular location are shown in Table S1. Shading indicates different enzyme classes: oxidoreductase (black), hydrolase (magenta), transferase (red), antioxidant (green), and other (blue).

XME profiles of RHE, HaCaT cells, EpiSkin and 2 different donors (254 and 1188) of the Epiderm-200 model were determined by proteomics. The number of unique tryptic peptides detected for each protein by liquid chromatography-tandem mass spectroscopy (LC-MS/MS) was used as an indirect measure of the relative quantity of each protein (Figure 2). Overall the profiles of expression of XMEs amongst the models are very similar. The majority of the XMEs present in the *in vitro* models were also present in whole human skin (Figure 2). However, some XMEs that were readily detected in whole skin were not detected in the *in vitro* model cells including epoxide hydrolase 1, liver carboxylesterase 1, alcohol dehydrogenase 1B and 4, aldehyde dehydrogenase 1L1, membrane primary amine oxidase, amine oxidase B, aldehyde oxidase and GST theta (Figure 2). On the other hand some proteins were detected in all or most of the *in vitro* models that were not detected in whole skin, including aldehyde dehydrogenase 7A1, NADPH-cytochrome P450 reductase and sulfotransferase 2B1 (Figure 2).

### Stability of XME expression during culture of the EpiDerm-200 model

**Figure 3:**
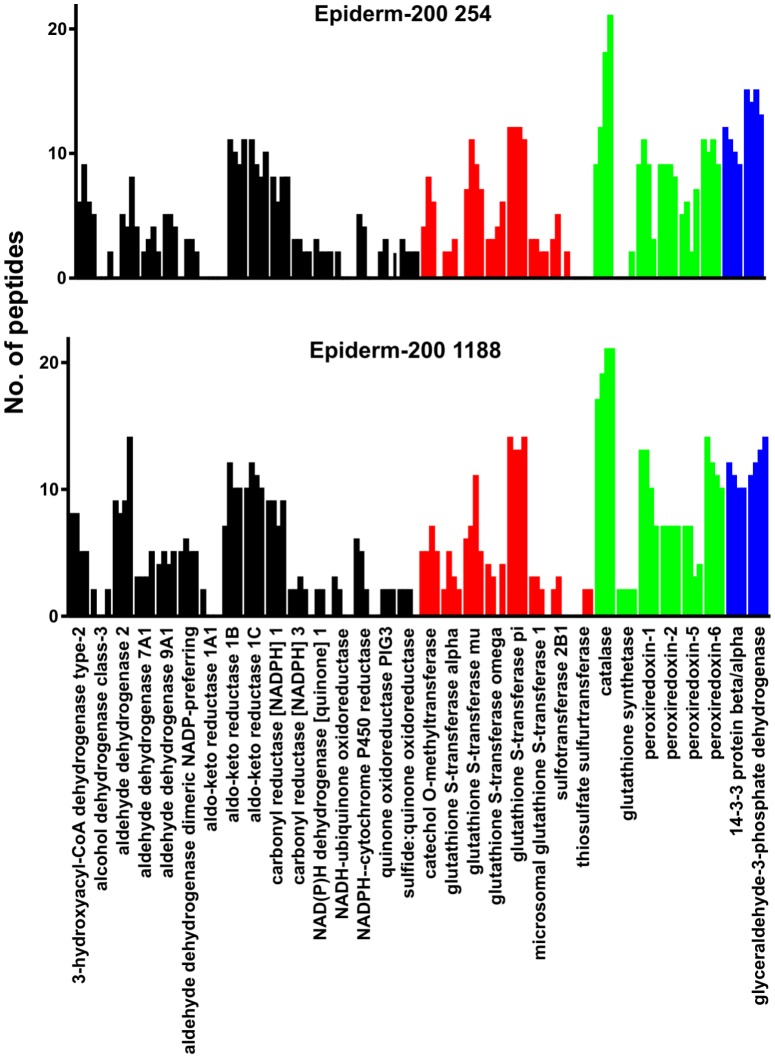
Stability of Epiderm-200 XME expression profiles in culture. Epiderm-200 cultures were derived from either donor 254 or 1188 and were maintained for up to 3 days. The relative amount of each protein is indicated by the number of different tryptic peptides specific to each protein or protein family that were detected with adjacent bars representing results from 0, 1, 2 and 3 days, respectively. Details of the protein accession numbers and their subcellular location are shown in Table S2. Shading indicates different enzyme classes: oxidoreductase (black), transferase (red), antioxidant (green), and other (blue).

The Epiderm-200 model is often used for genotoxicity studies [12] and such assessments are typically performed over several days. In order to determine whether the profile of XMEs varies over the course of such prolonged incubations, Epiderm-200 derived from two donors (254 and 1188) were cultured for up to 3 days, sampling at 1 day intervals. On the basis of the number of unique tryptic peptides detected for each protein, the results show that, with just a few exceptions, the detected XMEs were present at similar levels over incubation times of up to 3 days in both EpiDerm-200 models (Figure 3).

## Discussion

To our knowledge, this is the first study to offer a comprehensive overview of protein expression profiles of XMEs in human skin and *in vitro* skin models. To generate these data, a process by which proteomic data is analysed was devised to produce a defined and transparent (non-subjective) method by which XMEs were identified. As the identification is based on an inclusion and exclusion keyword list, the approach can be modified easily to identify other groups of proteins. The use of the PROTSIFT software allows the major part of the data analysis to take place in an automated fashion, minimizing human error and enabling easy re-analysis of large mass spectrometry datasets using different criteria to explore the data. The approach relies on the accuracy of the keywords used to extract data from the proteins of relevance, in this case to those involved in the biotransformation of chemicals. In an attempt to capture all relevant information a list of inclusion keywords were derived from gene ontology annotation data sources for positive identification of XMEs and to reduce the number of falsely assigned proteins an exclusion keyword list was also utilised. Overall, the majority of the proteins identified in this way appeared to have a clear function in chemical biotransformation, although the process is limited by the extent of current knowledge and the lack of a clear definition an XME. Nevertheless, the process may be further refined to include or exclude proteins based on further or more specific knowledge of the function of the proteins involved.

**Figure 4.Potential:**
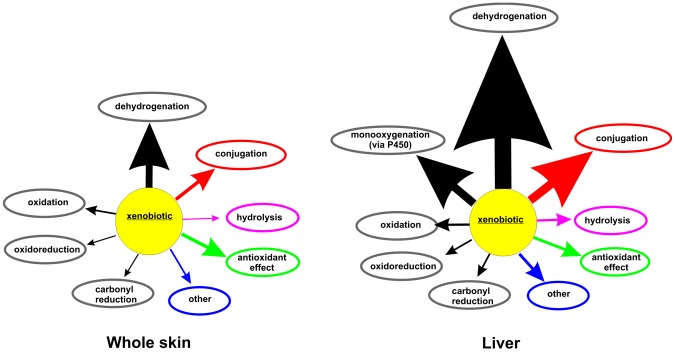
routes of xenobiotic metabolism in skin and liver. The size of each arrow is proportional to the number of XMEs detected that may catalyse each bioconversion indicated.

Overall the profile of detected XMEs in skin indicates a capacity for the phase I metabolism of alcohols through dehydrogenation, aldehydes through dehydrogenation and oxidation, amines through oxidation, carbonyls through reduction, and epoxides and carboxylesters through hydrolysis, whereas phase II metabolism is represented by several forms of GST. Examples of compounds metabolised this way in human skin include aliphatic alcohols [13], cinnamaldehyde, cinnamic alcohol [14], 5-hydroxytryptamine [15], capsaicin [16], betamethasone 17-valerate and propranolol [17]. Skin also appears to possess an antioxidant capacity as it contains catalase, glutathione peroxidase 3 and several peroxiredoxin forms. Overall, whole skin contains a reasonable diversity of XMEs that facilitate a range of biotransformation reactions (Figure 4). Almost all of the XMEs detected in skin are also present in liver. In general the relative levels are higher in liver and liver contains more XMEs in each class. Thus, the routes of metabolism in skin are likely to be more limited than those that occur in liver. There were exceptions to this as skin contained some proteins that were not detected in liver, including prostacyclin synthase (CYP8), and membrane primary amine oxidase, as well as some other proteins, such as alcohol dehydrogenase class-3 and GST pi, that were expressed at similar or greater levels in skin compared to liver. GST pi has been shown to be the predominant form of GST in epidermis [18] but not in the liver, where the alpha and mu gene families are highly expressed [19].

Many of the XMEs in skin identified at the protein level have also been identified at the RNA level. If an arbitrary threshold of 200,000 copies per µg RNA is considered then almost all of the gene products found in a study of human whole skin are represented here as proteins [20]. Similarly, those gene products detected above a signal intensity threshold value of 10,000 in a microarray approach to examine expression in whole skin [21] are consistent with the proteins identified in this present study. Support for the identification of alcohol/aldehyde dehydrogenase and esterase enzymes is from enzyme activity assays performed on whole human skin [3]. These workers also detected sulfotransferase activity, whereas the proteomics approach used here failed to detect such an enzyme in whole skin although detection was evident in the *in vitro* models.

CYP enzymes in families 1–3 metabolise xenobiotics and none of these were detected in whole skin or any of the *in vitro* skin models of skin by either proteomics or immunoblotting. This contrasts with liver where 13 CYP XMEs, encompassing all of the major forms involved in xenobiotic metabolism, were detected by proteomics and by immunochemical methods [9]. These results are quite different from those found in rat skin where expression of several CYP proteins was evident [22]. There have been a number of publications that claim the presence of various members of the CYP1, CYP2 and CYP3 families in skin, some on the basis of gene expression and others by immunochemical detection of the proteins [1]; [2]; [23]; [24]; [24]. The use of PCR to detect mRNA species is very sensitive but the relationship of low mRNA levels to CYP protein expression levels is uncertain (e.g. [25]), although, as described above, when quantified, low RNA levels in skin appear to correspond to low levels of protein expression. Immunochemical methods of detection are dependent on both the method and the properties of the antibody used, particularly with respect to its specificity and sensitivity. It is possible that some of the previous results may be explained by the use of antibodies with markedly superior sensitivity. However, this does not seem likely, and as none of the previous studies indicate the sensitivity of the immunochemical methods used it is not possible to compare those results with this current study, nevertheless, under the conditions specified here, the LC-MS/MS method applied is about 25-times more sensitive than immunoblotting and the specificity is assured by the MS identification of characteristic tryptic peptides. Based on the limit of detection of CYPs by LC-MS/MS of 0.1 pmol/mg microsomal protein it is possible to make an assessment of the likely relative capacity of skin to perform CYP-catalysed reactions compared with liver and hence place the results in some rational context. The finding that skin CYP levels are at least 300-fold lower than those of liver indicates that the rate of oxidative biotransformation of a xenobiotic catalysed by any CYP enzyme present will be very low, limiting its contribution to overall dermal metabolism.

The lack of detection of NAT in skin by proteomics is perhaps surprising, although this is consistent with the levels of NAT1 and NAT2 mRNA which have been found to be very low or undetectable in human epidermis and dermis [20]. A previous study has determined that the specific activity of pure recombinant human NAT1 is 254 µmol/min/mg NAT1 (i.e. 8.62 nmol/min/pmol NAT1) using p-amino benzoic acid as substrate [26]. On this basis the limit of detection possible by proteomics of 3 pmol NAT1/mg cytosolic protein equates to an activity of ∼25 nmol/min/mg cytosolic protein and this value exceeds that of skin cytosol and liver cytosol by approximately 3- or 10-fold, respectively [27], [28]. Thus, measurements of NAT levels by enzyme activity are more sensitive than the proteomics approach used here.

The proteomics approach is limited by the ability to detect a sufficient number of tryptic peptides derived from each protein. Digestion of a single protein with trypsin will yield many peptides, the number being proportional to the size of the protein and the frequency of occurrence of trypsin-sensitive sites. The detection of peptides by LC-MS is dependent on the size of the peptides, their ionisation in the electrospray, separation by LC, and both the resolution and sensitivity on the MS used. Thus, not all tryptic peptides will be identified and indeed it is generally sufficient if just two peptides can be clearly identified provided adequate fragmentation data can be obtained and the peptides identified occur in just one protein. Nevertheless, in cases where more peptides are identified, this indicates not only a greater abundance of a protein but also leads to an improved certainty of the identification of that protein. Relative label-free quantification, as used here, is based on the same criteria of identification and utilises peptide ion intensity to compare levels, thus those peptides that ionise well provide the clearest data and as we and others have shown previously the relationship between ion intensity and quantity holds for up to a range of 3-orders of magnitude [10]. As with all methods, the current approach is limited in its sensitivity. Improved sensitivity could be obtained by the use of an MS with better inherent resolution and accuracy and also by the use of MS methods with improved quantitative capacity such as multiple reaction monitoring [29]. It is possible that with the application of such methods and/or superior instrumentation that the levels of CYP and NAT proteins in skin samples may be detected and quantified.

The XME profiles of the *in vitro* skin models were similar to one another, suggesting that many biotransformation pathways are comparable. A number of XMEs that were identified in whole human skin were not detected in the *in vitro* models. Lower levels of mRNA encoding for several XMEs including epoxide hydrolase 1 have been found in the epidermis compared with the dermis [20]. It is possible therefore that the *in vitro* skin models which are epidermal in origin may contain lower levels of some XMEs. Interestingly though, the *in vitro* models contain some important enzymes including NADPH-cytochrome P450 reductase, aldehyde dehydrogenase 7A1 and sulfur transferase enzymes that were not detected in whole skin. This may be due to the culture conditions in which the models were maintained. Sulfotransferase 2B1b has been shown to be expressed at higher levels in normal human epidermal keratinocytes after culture, especially when the medium contains increased levels of calcium [30]. The stability of XME expression profiles of both Epiderm-200 models were examined and found to be similar for incubation times of up to 3 days. The main exception to this was NADPH-cytochrome P450 reductase, which was readily detected in both models on day 0, but levels decreased during culture until by 3 days was undetectable. Overall though, the profiles appeared relatively stable, suggesting their suitability for studies requiring longer culture times.

In conclusion, a proteomics approach has been successfully applied to make a comprehensive analysis of the biotransformation characteristics of whole human skin and various *in vitro* skin models. The results indicate that skin contains a range of XMEs capable of metabolising a variety of classes of chemicals. However, the capacity for CYP-mediated metabolism of xenobiotics in skin appears to be very low in comparison with liver. The various *in vitro* models of human skin examined had profiles similar to one another and to that of whole skin. In the case of the EpiDerm models the levels of enzymes remained stable for at least 3 days, supporting their suitability in tests that require relatively long exposure times, such as those used for the assessment of genotoxicity. Overall, these data should help in the development of a rational basis for understanding the fate of xenobiotics in skin.

## Materials and Methods

### Sample preparation

Samples of human whole skin were obtained from healthy females undergoing reduction mammoplasty (n = 10) at the Kaiserswerther Diakonie hospital in Düsseldorf, Germany. The mean age was 44±13 years. During the procedure patients were sedated with propofol and/or remifentanil; none were receiving regular medication. Patients gave their written consent for the excess skin removed to be used for scientific research purposes and the project, which followed the Declaration of Helsinki protocols, was approved by the Ethickkommission der Medizinischen Fakultät der Heinrich-Heine-Universität, Düsseldorf. Skin samples were collected immediately following surgery, chilled on ice during transportation to the laboratory and then stored at −80°C until processed. Subcutaneous tissue was carefully removed before skin samples were cut into small pieces, and then homogenized (SW18, Ultra-Turrax, Germany) in 1.5-volumes of ice-cold 250 mM potassium phosphate buffer, pH 7.25 containing 150 mM KCl and 1 mM EDTA. Microsomal and cytosol fractions were prepared by differential centrifugation as described previously [31] and stored at −80°C until required. Human liver microsomal and cytosolic fractions had been prepared previously and stored at −80°C [9]. Epiderm-200 (MatTek Corporation, Ashland, MA, USA), RHE (SkinEthic Laboratories, Lyon, France), and EpiSkin (SkinEthic Laboratories) were cultured using the medium provided by the respective manufacturer until analysis. The stability of XME expression in Epiderm-200 was determined after the normal procedure to establish the 3-dimensional cultures and then after culture for 1, 2 and 3 days; in these experiments the medium was replaced daily. HaCaT cells (German Cancer Research Center, Heidelberg, Germany) were cultured in T75 flasks at 37°C with 5% CO_2_ in DMEM supplemented with 10% fetal bovine serum, 100 IU penicillin/streptomycin and 2 mM glutamine. The medium was changed every 3 days and cells were sub-cultured when confluent. The cultures were washed with PBS before being homogenized and subcellular fractions prepared as described for human skin.

### Immunoblotting and CYP content

Immunoblotting was performed using up to 75 µg of microsomal protein and rabbit antibodies targeted against either CYP1A1, CYP1A2, CYP2A6, CYP2B6, CYP2C forms, CYP2D6, CYP2E1, CYP3A4, CYP3A5, or CYP4A11, as described previously [9]. A preparation of recombinant CYP1A1 protein expressed in insect cell microsomal fraction was purchased from Gentest BD (Franklin Lakes, NJ USA). Estimation of the apoprotein content of CYP1A1, CYP1A2, CYP2E1, CYP3A4 and CYP3A5 was determined immunochemically using synthetic peptides as standards as described previously [32]. The limit of detection by immunoblotting was determined as described in the Figure S1.

### Enzyme activity assays

Ethoxyresorufin O-deethylase activity was determined as described previously [33]. The assay mixture contained 2 µM ethoxyresorufin and 5% final concentration of microsomal protein. NAT activity was estimated using p-toluidene as substrate as described previously [11]. GST activity was measured according to an established protocol using 1-chloro-2,4-dinitrobenzene as the substrate [34].

### Proteomics

**Figure 5:**
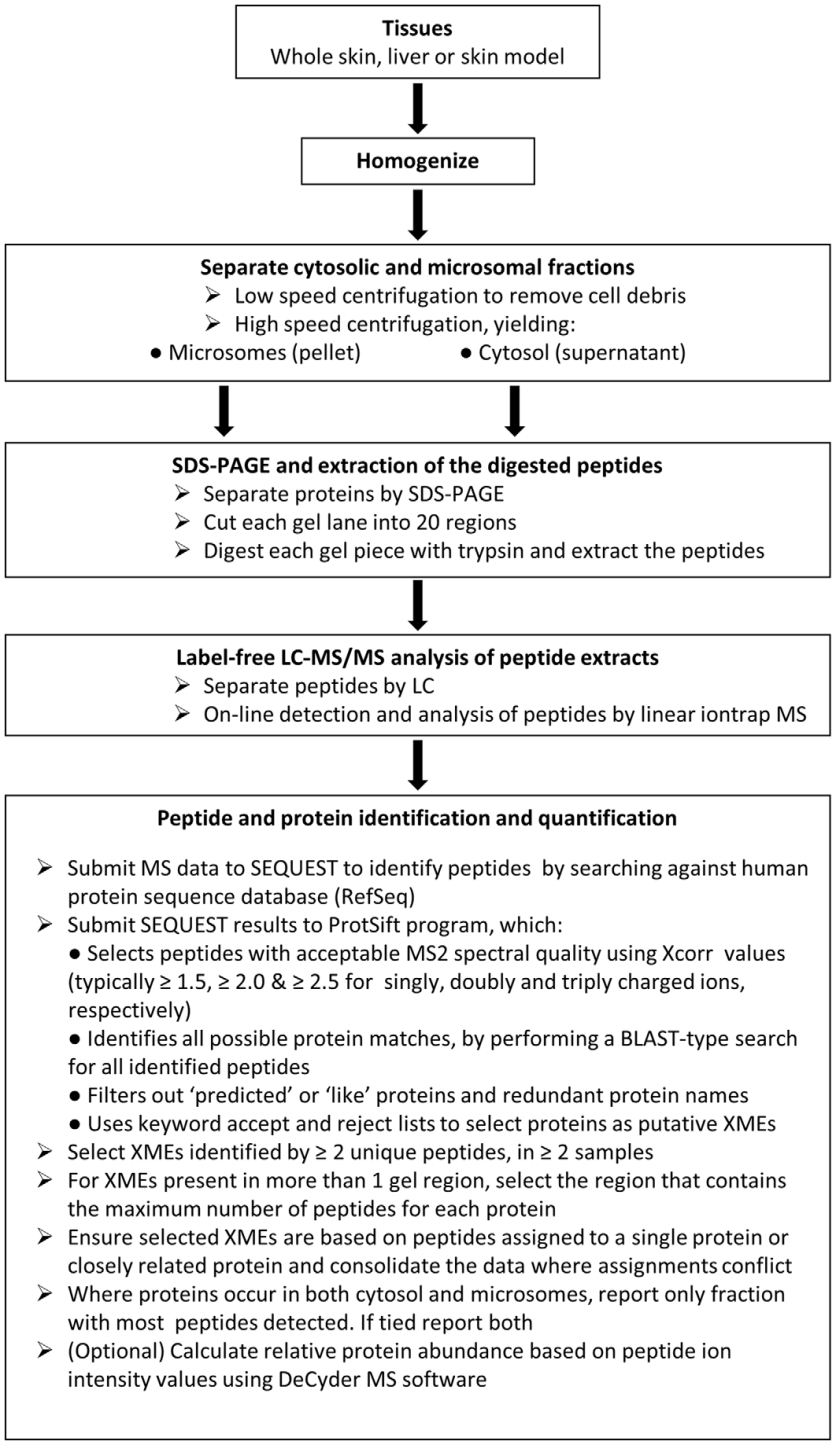
Flow diagram summarizing the methodology.

The workflow employed is summarized in figure 5. The protein content of all samples was estimated using the bicinchoninic acid method (Thermo Fischer Scientific, Cramlington, UK) with bovine albumin as the calibration standard. Proteins in microsomal and cytosol fractions from skin, skin models and liver samples were separated using 10% NuPAGE Novex bis-tris gels (Invitrogen Ltd., Paisley, UK). Gels were stained with InstantBlue® and each sample-containing lane cut into a series of 20 regions based on the position of molecular weight markers (SeaBlue Marker®, Invitrogen Ltd., Paisley, UK) and the distribution of proteins observed in the gel. Each gel piece was then digested with trypsin, peptides extracted and dried. Dried samples were reconstituted, injected onto a reverse phase column and the eluted peptides analysed by liquid chromatography tandem mass spectrometry on-line using an Agilent 1200 LC series (Agilent Technologies UK Ltd., Berkshire, UK) and a Thermo LTQ linear ion trap MS (Thermo Scientific, Hemel Hempstead, UK) as described previously [35].

Data analysis was performed essentially as described previously [35] with some modifications. Analysis was restricted to those proteins with a putative role in xenobiotic metabolism and these were selected in an automated fashion using PROTSIFT (https://github.com/jcupitt/protsift), which is software that was written for this purpose to allow application of a transparent procedure. In the process protein names containing the word ‘hypothetical’ or ‘like’ were filtered out automatically. The selection of proteins as putative XMEs also took place in an automated fashion, based on a keyword list search. To assemble the keyword list, a number of sources were used. Relevant enzyme category names were taken from [36] and combined with the results of a search on the AmiGO website (http://amigo.geneontology.org) for gene product associations to the term “xenobiotic metabolic process” (GO term 0006805). Also, gene products referred to in some recent studies examining XME expression at the RNA level [20], [21] were included. Proteins that were obviously not involved in xenobiotic metabolism were eliminated by use of a reject list to give a final list of putative XME proteins.

The PROTSIFT enzyme accept list comprised: 3-Hydroxyacyl-CoA dehydrogenase, acyl-coenzyme A synthetase ACSM1, adrenodoxin, alcohol dehydrogenase, aldehyde dehydrogenase, aldehyde oxidase, aldo-keto reductase, amine oxidase, arylacetamide deacetylase-like 1, bifunctional 3′-phosphoadenosine 5′-phosphosulfate synthase, carbonyl reductase, carboxylesterase, catalase, catechol-O-methyltransferase, catechol O-methyltransferase, cytochrome P450, dehydrogenase/reductase SDR family member, dopamine beta-hydroxylase, epoxide hydrolase, gamma-glutamyl carboxylase, gamma-glutamyltranspeptidase, glucuronidase, glutamate–cysteine ligase, glutathione peroxidase, glutathione transferase, glutathione synthetase, hepatocyte nuclear factor 4-alpha, histamine N-methyltransferase, hydroxysteroid dehydrogenase, kynureninase, lactoperoxidase, long-chain-fatty-acid–CoA ligase 1, monooxygenase, N-acetyltransferase, NAD\(P\)H dehydrogenase \[quinone\] 1, NAD(P) transhydrogenase, mitochondrial, nicotinamide N-methyltransferase, nitric oxide synthase, nuclear receptor subfamily 1 group I member 2, peroxiredoxin, prostaglandin G/H synthase, prostacyclin synthase, protein S100-A12, quinone oxidoreductase, steryl-sulfatase, sulfotransferase, thiopurine S-methyltransferase, thiosulfate sulfurtransferase, thyroid peroxidase, trans-1,2-dihydrobenzene-1,2-diol dehydrogenase, thromboxane-A synthase, and xanthine dehydrogenase. The PROTSIFT enzyme reject list comprised: carbohydrate, heparan, glucose-6-phosphate dehydrogenase, protein-glutamine gamma-glutamyltransferase, and dolichyl-diphosphooligosaccharide-protein glycosyltransferase.

Application of the keyword search of the whole human RefSeq database (version 38) resulted in a list of 443 proteins identified as potential XMEs from a total number of proteins of 38,783. These proteins are listed in Table S3.

The issue of redundancy in identified proteins was addressed by including all possible assignments in the primary results table. The peptides used to identify each protein were then examined to determine if they occurred in the sequence of any other protein in the database and these occurrences were also listed. The uniqueness (or otherwise) of the assignment of peptides to the sequence of one or more proteins was used to determine whether an assignment could be made to a specific protein or to a group of related proteins (such as isoforms). Where it was not possible to distinguish between related proteins from tryptic peptide data due to their close structural identity the data has been consolidated into a root description of the protein e.g. data from peptides overlapping in their assignment to one or more of the GST alpha isoforms 1, 2, 3 and 5 have been combined and the assignment reported as GST alpha. Hence proteins may be represented by a single accession number or a series of accession numbers. For proteins detected in more than one gel region, only data from the region that contained the greatest number of detected peptides for each protein was considered. Data from cytosolic and microsomal fractions were combined. Where proteins were found in both fractions, the fraction in which the protein was most readily detected is reported. When similar levels were detected in both fractions, and their location as such was confirmed using Uniprot (uniprot.org), both are reported. Details of the peptides detected for each protein are listing in the Table S4.

In some cases, limits of detection of proteins were determined by spiking samples of microsomal fraction or cytosol with recombinant CYPs (Gentest BD) or NAT1 with an N-terminal GST tag (Abnova, Taipei, Taiwan).

### Statistical analyses

Fold difference values were calculated on the basis of the summed ion intensities of the component peptides, using DeCyder MS (Version 2.0, GE Healthcare Biosciensces, Uppsala, Sweden) software. Statistical significance was determined by the Mann-Whitney U-test, using GraphPad Prism (version 4.00 for Windows, GraphPad Software, San Diego, California, USA).

## Supporting Information

Figure S1 **Examples of quantitation of CYP enzymes by immunoblotting.** In each case, respective synthetic peptide (coupled to lysozyme) or recombinant protein antigen was loaded onto SDS-polyacrylamide gels in quantities of up to 10 pmol and developed with rabbit antibodies raised against synthetic peptides corresponding to each of the CYP enzymes that were first immunodepleted of antibodies against carrier protein and coupling linkage as described previously [37]. Immunoreactivity was detected using a goat anti-rabbit IgG-horseradish peroxidase and visualised by enhanced chemiluminescence that was recorded on Hyperfilm and analysed using a Kodak Image Station as indicated in the Methods section. Each point represents the mean and SEM of at least 4 determinations. The relationship between the quantity loaded and the intensity of immunoreactivity and was reasonably linear for loadings of up to 1 pmol antigen but flattened with greater quantities. The least amount of antigen that could be detected was typically 0.2 pmol, which is equivalent to 2.5 pmol/mg microsomal protein for a sample loading of 75 µg microsomal protein.
(TIF)

Table S1 **Details of XME proteins detected in whole skin and** ***in vitro*** **skin models.** NCBI numbers for each protein and for all members of groups of related proteins are shown. The subcellular fraction in which each protein was principally detected is also indicated.
(DOCX)

Table S2 **Details of XME proteins detected in Epiderm-200.** NCBI numbers for each protein and for all members of groups of related proteins are shown. The subcellular fraction in which each protein was principally detected is also indicated.
(DOCX)

Table S3 **Proteins identified as putative XMEs in Refseq (version 38) based on a keyword search of names.**
(DOCX)

Table S4 **Summary of the proteomic data upon which protein identifications are based.** Each protein or family of proteins identified is listed in alphabetical order and is accompanied by corresponding NCBI numbers. The subcellular fraction containing the majority of the peptides for each protein is shown (microsomal: M or cytosolic: C) as well as the Sf value, which indicates the certainty of the protein identification in each group of samples. Details of the peptide sequences and charge upon which protein identity is based are shown, and the quality of the match of MS data to these sequences is indicated by Xcorr values that are shown for each group of samples. A BLAST-type search for each peptide sequence was performed and where matches to other proteins occur there are indicated by corresponding NCBI reference numbers.
(DOCX)
