# Supplementary figures and images for: Elucidation of Xenobiotic Metabolism Pathways in Human Skin and Human Skin Models by Proteomic Profiling
Source: PLoS One. 2012 Jul 26;7(7):e41721. doi: 10.1371/journal.pone.0041721 (PMC3406074; doi:10.1371/journal.pone.0041721)

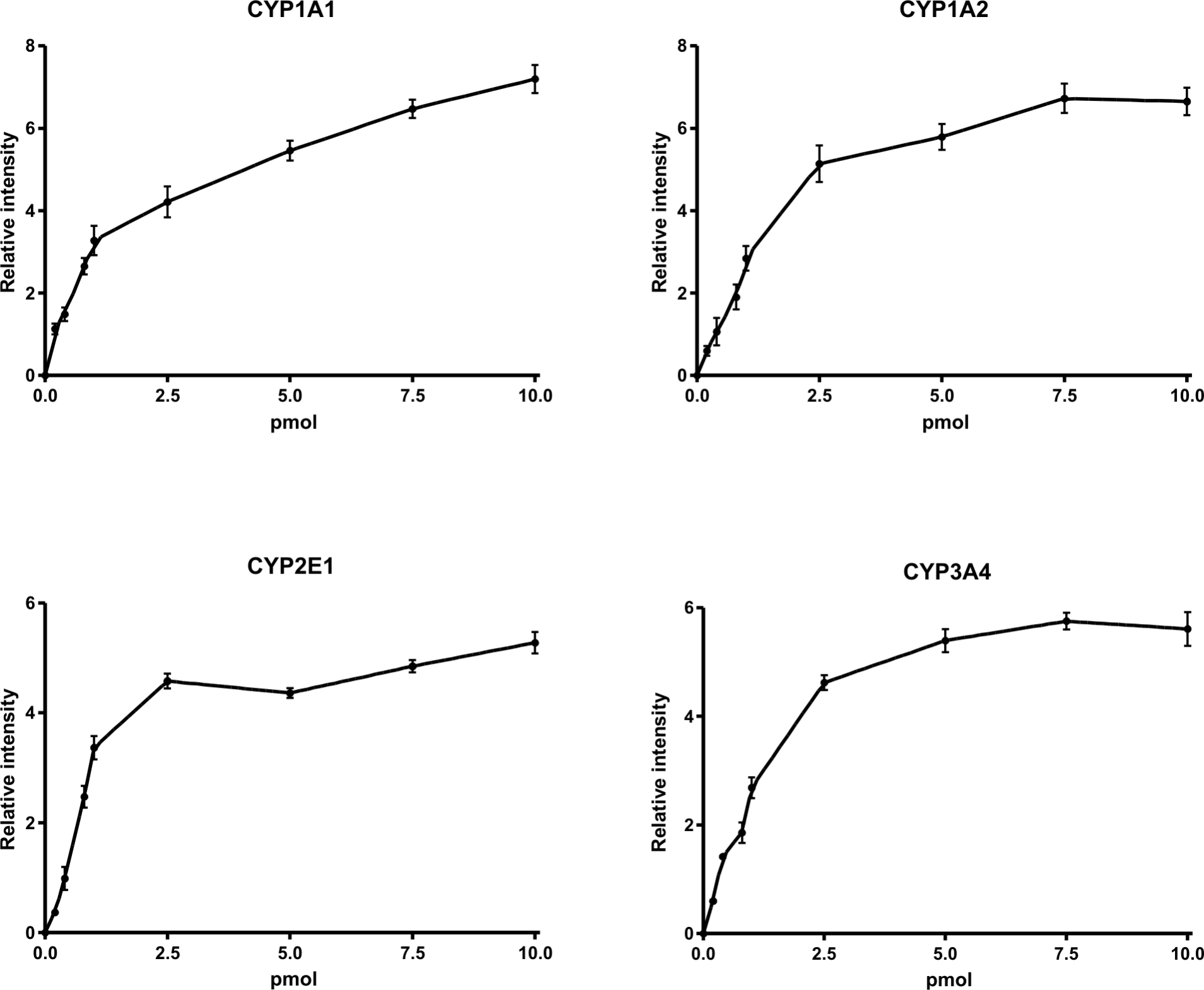

Supplement: Figure S1 — Examples of quantitation of CYP enzymes by immunoblotting. In each case, respective synthetic peptide (coupled to lysozyme) or recombinant protein antigen was loaded onto SDS-polyacrylamide gels in quantities of up to 10 pmol and developed with rabbit antibodies raised against synthetic peptides corresponding to each of the CYP enzymes that were first immunodepleted of antibodies against carrier protein and coupling linkage as described previously [37]. Immunoreactivity was detected using a goat anti-rabbit IgG-horseradish peroxidase and visualised by enhanced chemiluminescence that was recorded on Hyperfilm and analysed using a Kodak Image Station as indicated in the Methods section. Each point represents the mean and SEM of at least 4 determinations. The relationship between the quantity loaded and the intensity of immunoreactivity and was reasonably linear for loadings of up to 1 pmol antigen but flattened with greater quantities. The least amount of antigen that could be detected was typically 0.2 pmol, which is equivalent to 2.5 pmol/mg microsomal protein for a sample loading of 75 µg microsomal protein. (TIF) [file pone.0041721.s001.tif]
