# Supplementary material for: Elucidation of Xenobiotic Metabolism Pathways in Human Skin and Human Skin Models by Proteomic Profiling
Source: PLoS One. 2012 Jul 26;7(7):e41721. doi: 10.1371/journal.pone.0041721 (PMC3406074; doi:10.1371/journal.pone.0041721)
Supplement: Table S1 — Details of XME proteins detected in whole skin and in vitro skin models. NCBI numbers for each protein and for all members of groups of related proteins are shown. The subcellular fraction in which each protein was principally detected is also indicated. (DOCX) [file pone.0041721.s002.docx]

| Protein | NCBI number | Fraction |
| --- | --- | --- |
| alcohol dehydrogenase 1B | NP_000659.2 | cytosol |
| alcohol dehydrogenase 4 | NP_000661.2 | cytosol |
| aldehyde dehydrogenase 1L1 | NP_036322.2 | cytosol |
| aldehyde oxidase | NP_001150.3 | cytosol |
| amine oxidase [flavin-containing] B | NP_000889.3 | microsome |
| membrane primary amine oxidase | NP_003725.1 | microsome |
| prostacyclin synthase | NP_000952.1 | microsome |
| short-chain dehydrogenase/reductase 7 | NP_057113.1, NP_001099041.1, NP_056325.2 | microsome |
| epoxide hydrolase 1 | NP_001129490.1, NP_000111.1 | microsome |
| liver carboxylesterase 1 | NP_001020366.1, NP_001020365.1, NP_001257.4 | microsome |
| gamma-glutamyltransferase 5 | NP_001093252.1, NP_004112.2, NP_001093251.1 | microsome |
| glutathione S-transferase theta | NP_000845.1, NP_001074312.1, NP_000844.2 | cytosol |
| glutathione peroxidase 3 | NP_002075.2 | cytosol |
| long-chain-fatty-acid--CoA ligase 1 | NP_004449.1, NP_055977.3, NP_001986.2 | microsome |
| 3-hydroxyacyl-CoA dehydrogenase type-2 | NP_004484.1, NP_001032900.1 | cytosol |
| alcohol dehydrogenase class-3 | NP_000662.3 | cytosol |
| aldehyde dehydrogenase 1A1 | NP_000680.2 | cytosol |
| aldehyde dehydrogenase 2 | NP_000681.2 | cytosol |
| aldehyde dehydrogenase 3A2 | NP_001026976.1, NP_000373.1 | microsome |
| aldehyde dehydrogenase 9A1 | NP_000687.3 | cytosol |
| aldo-keto reductase 1A1 | NP_697021.1, NP_006057.1 | cytosol |
| aldo-keto reductase 1C | NP_001809.2, NP_995317.1, NP_001128713.1, NP_001345.1, NP_001344.2, NP_003730.4 | cytosol |
| carbonyl reductase [NADPH] 1 | NP_001748.1 | cytosol |
| carbonyl reductase [NADPH] 3 | NP_001227.1 | cytosol |
| NADH-ubiquinone oxidoreductase | NP_004997.4 | microsome |
| sulfide:quinone oxidoreductase | NP_067022.1 | microsome |
| glutathione S-transferase alpha | NP_665683.1, NP_001503.1, NP_714543.1, NP_000837.3, NP_000838.3 | cytosol |
| glutathione S-transferase mu | NP_671489.1, NP_001135840.1, NP_666533.1, NP_000552.2, NP_000840.2, NP_000842.2, NP_000841.1, NP_000839.1 | cytosol |
| glutathione S-transferase omega | NP_899062.1, NP_004823.1 | cytosol |
| glutathione S-transferase pi | NP_000843.1 | cytosol |
| microsomal glutathione S-transferase 1 | NP_665734.1, NP_665735.1, NP_064696.1, NP_665707.1 | microsome |
| catalase | NP_001743.1 | cytosol |
| glutathione synthetase | NP_000169.1 | cytosol |
| peroxiredoxin-1 | NP_002565.1, NP_859047.1, NP_859048.1 | cytosol |
| peroxiredoxin-2 | NP_005800.3, NP_859428.1 | cytosol |
| peroxiredoxin-5 | NP_857635.1, NP_857634.1, NP_036226.1 | cytosol |
| peroxiredoxin-6 | NP_004896.1 | cytosol |
| 14-3-3 protein beta/alpha | NP_003395.1, NP_647539.1 | cytosol |
| glyceraldehyde-3-phosphate dehydrogenase | NP_002037.2 | cytosol |
| aldehyde dehydrogenase 7A1 | NP_001173.2 | cytosol |
| aldehyde dehydrogenase dimeric NADP-preferring | NP_000682.3, NP_001128639.1, NP_001128640.1 | cytosol |
| aldo-keto reductase 1B | NP_001074007.2, NP_064695.3 | cytosol |
| NAD(P)H dehydrogenase [quinone] 1 | NP_000894.1, NP_001020605.1, NP_001020604.1 | cytosol |
| NADPH--cytochrome P450 reductase | NP_000932.3 | microsome |
| quinone oxidoreductase PIG3 | NP_004872.2, NP_671713.1 | cytosol |
| N-acetyltransferase 10 | NP_001137502.1, NP_078938.2 | cytosol |
| catechol O-methyltransferase | NP_000745.1, NP_009294.1, NP_001128633.1, NP_001128634.1 | cytosol |
| sulfotransferase 2B1 | NP_004596.2, NP_814444.1 | cytosol |
| thiosulfate sulfurtransferase | NP_003303.2 | cytosol |
