# Supplementary material for: Elucidation of Xenobiotic Metabolism Pathways in Human Skin and Human Skin Models by Proteomic Profiling
Source: PLoS One. 2012 Jul 26;7(7):e41721. doi: 10.1371/journal.pone.0041721 (PMC3406074; doi:10.1371/journal.pone.0041721)
Supplement: Table S2 — Details of XME proteins detected in Epiderm-200. NCBI numbers for each protein and for all members of groups of related proteins are shown. The subcellular fraction in which each protein was principally detected is also indicated. (DOCX) [file pone.0041721.s003.docx]

| Protein | NCBI number | Fraction |
| --- | --- | --- |
| 3-hydroxyacyl-CoA dehydrogenase type-2 | NP_004484.1, NP_001032900.1 | cytosol |
| alcohol dehydrogenase class-3 | NP_000662.3 | cytosol |
| aldehyde dehydrogenase 2 | NP_000681.2 | cytosol |
| aldehyde dehydrogenase 7A1 | NP_001173.2 | cytosol |
| aldehyde dehydrogenase 9A1 | NP_000687.3 | cytosol |
| aldehyde dehydrogenase dimeric NADP-preferring | NP_000682.3, NP_001128639.1, NP_001128640.1 | cytosol |
| aldo-keto reductase 1A1 | NP_697021.1, NP_006057.1 | cytosol |
| aldo-keto reductase 1B | NP_001074007.2, NP_064695.3 | cytosol |
| aldo-keto reductase 1C | NP_001809.2, NP_995317.1, NP_001128713.1, NP_001345.1, NP_001344.2, NP_003730.4 | cytosol |
| carbonyl reductase [NADPH] 1 | NP_001748.1 | cytosol |
| carbonyl reductase [NADPH] 3 | NP_001227.1 | cytosol |
| NAD(P)H dehydrogenase [quinone] 1 | NP_000894.1, NP_001020605.1, NP_001020604.1 | cytosol |
| NADH-ubiquinone oxidoreductase | NP_004997.4 | microsome |
| NADPH--cytochrome P450 reductase | NP_000932.3 | microsome |
| quinone oxidoreductase PIG3 | NP_004872.2, NP_671713.1 | cytosol |
| sulfide:quinone oxidoreductase | NP_067022.1 | microsome |
| catechol O-methyltransferase | NP_000745.1, NP_009294.1, NP_001128633.1, NP_001128634.1 | cytosol |
| glutathione S-transferase alpha | NP_665683.1, NP_001503.1, NP_714543.1, NP_000837.3, NP_000838.3 | cytosol |
| glutathione S-transferase mu | NP_671489.1, NP_001135840.1, NP_666533.1, NP_000552.2, NP_000840.2, NP_000842.2, NP_000841.1, NP_000839.1 | cytosol |
| glutathione S-transferase omega | NP_899062.1, NP_004823.1 | cytosol |
| glutathione S-transferase pi | NP_000843.1 | cytosol |
| microsomal glutathione S-transferase 1 | NP_665734.1, NP_665735.1, NP_064696.1, NP_665707.1 | microsome |
| sulfotransferase 2B1 | NP_004596.2, NP_814444.1 | cytosol |
| thiosulfate sulfurtransferase | NP_003303.2 | cytosol |
| catalase | NP_001743.1 | cytosol |
| glutathione synthetase | NP_000169.1 | cytosol |
| peroxiredoxin-1 | NP_002565.1, NP_859047.1, NP_859048.1 | cytosol |
| peroxiredoxin-2 | NP_005800.3, NP_859428.1 | cytosol |
| peroxiredoxin-5 | NP_857635.1, NP_857634.1, NP_036226.1 | cytosol |
| peroxiredoxin-6 | NP_004896.1 | cytosol |
| 14-3-3 protein beta/alpha | NP_003395.1, NP_647539.1 | cytosol |
| glyceraldehyde-3-phosphate dehydrogenase | NP_002037.2 | cytosol |
