# Supplementary material for: Elucidation of Xenobiotic Metabolism Pathways in Human Skin and Human Skin Models by Proteomic Profiling
Source: PLoS One. 2012 Jul 26;7(7):e41721. doi: 10.1371/journal.pone.0041721 (PMC3406074; doi:10.1371/journal.pone.0041721)
Supplement: Table S3 — Proteins identified as putative XMEs in Refseq (version 38) based on a keyword search of names. (DOCX) [file pone.0041721.s004.docx]

| **NCBI number** | **Protein description** |
| --- | --- |
| NP_861420.1 | 11-beta-hydroxysteroid dehydrogenase 1 |
| NP_005516.1 | 11-beta-hydroxysteroid dehydrogenase 1 |
| NP_835236.2 | 17-beta-hydroxysteroid dehydrogenase 13 isoform A |
| NP_001129702.1 | 17-beta-hydroxysteroid dehydrogenase 13 isoform B |
| NP_057330.2 | 17-beta-hydroxysteroid dehydrogenase 14 |
| NP_003716.2 | 17-beta-hydroxysteroid dehydrogenase type 6 precursor |
| NP_079469.2 | 3 beta-hydroxysteroid dehydrogenase type 7 isoform a |
| NP_001136249.1 | 3 beta-hydroxysteroid dehydrogenase type 7 isoform b |
| NP_001136250.1 | 3 beta-hydroxysteroid dehydrogenase type 7 isoform b |
| NP_000853.1 | 3 beta-hydroxysteroid dehydrogenase/Delta 5--_4-isomerase type 1 |
| NP_001159592.1 | 3 beta-hydroxysteroid dehydrogenase/Delta 5--_4-isomerase type 2 |
| NP_000189.1 | 3 beta-hydroxysteroid dehydrogenase/Delta 5--_4-isomerase type 2 |
| NP_004484.1 | 3-hydroxyacyl-CoA dehydrogenase type-2 isoform 1 |
| NP_001032900.1 | 3-hydroxyacyl-CoA dehydrogenase type-2 isoform 2 |
| NP_443188.2 | acyl-coenzyme A synthetase ACSM1, mitochondrial |
| NP_004100.1 | adrenodoxin, mitochondrial precursor |
| NP_001026904.1 | adrenodoxin-like protein, mitochondrial precursor |
| NP_775925.1 | AER61 glycosyltransferase |
| NP_697021.1 | alcohol dehydrogenase |
| NP_006057.1 | alcohol dehydrogenase |
| NP_000658.1 | alcohol dehydrogenase 1A |
| NP_000659.2 | alcohol dehydrogenase 1B |
| NP_000660.1 | alcohol dehydrogenase 1C |
| NP_000661.2 | alcohol dehydrogenase 4 |
| NP_001095940.1 | alcohol dehydrogenase 6 isoform 1 |
| NP_000663.1 | alcohol dehydrogenase 6 isoform 2 |
| NP_000662.3 | alcohol dehydrogenase class-3 |
| NP_000680.2 | aldehyde dehydrogenase 1A1 |
| NP_036322.2 | aldehyde dehydrogenase 1L1 |
| NP_001026976.1 | aldehyde dehydrogenase 3A2 isoform 1 |
| NP_000373.1 | aldehyde dehydrogenase 3A2 isoform 2 |
| NP_001173.2 | aldehyde dehydrogenase 7 family, member A1 |
| NP_000687.3 | aldehyde dehydrogenase 9A1 |
| NP_000684.2 | aldehyde dehydrogenase family 1 member A3 |
| NP_699160.2 | aldehyde dehydrogenase family 16 member A1 isoform 1 |
| NP_001138868.1 | aldehyde dehydrogenase family 16 member A1 isoform 2 |
| NP_001154945.1 | aldehyde dehydrogenase family 3 member B1 isoform a |
| NP_000685.1 | aldehyde dehydrogenase family 3 member B1 isoform a |
| NP_001025181.1 | aldehyde dehydrogenase family 3 member B1 isoform b |
| NP_000686.2 | aldehyde dehydrogenase family 3 member B2 |
| NP_001026786.1 | aldehyde dehydrogenase family 3 member B2 |
| NP_072090.1 | aldehyde dehydrogenase family 8 member A1 isoform 1 |
| NP_739577.1 | aldehyde dehydrogenase family 8 member A1 isoform 2 |
| NP_000683.3 | aldehyde dehydrogenase X, mitochondrial precursor |
| NP_001128639.1 | aldehyde dehydrogenase, dimeric NADP-preferring |
| NP_000682.3 | aldehyde dehydrogenase, dimeric NADP-preferring |
| NP_001128640.1 | aldehyde dehydrogenase, dimeric NADP-preferring |
| NP_000681.2 | aldehyde dehydrogenase, mitochondrial precursor |
| NP_001150.3 | aldehyde oxidase |
| NP_064695.3 | aldo-keto reductase family 1 member B10 |
| NP_001344.2 | aldo-keto reductase family 1 member C1 |
| NP_001345.1 | aldo-keto reductase family 1 member C2 isoform 1 |
| NP_995317.1 | aldo-keto reductase family 1 member C2 isoform 1 |
| NP_001128713.1 | aldo-keto reductase family 1 member C2 isoform 2 |
| NP_003730.4 | aldo-keto reductase family 1 member C3 |
| NP_001809.2 | aldo-keto reductase family 1 member C4 |
| NP_001074007.2 | aldo-keto reductase family 1, member B15 |
| NP_001035267.1 | aldo-keto reductase family 1, member E2 |
| NP_001082.2 | amiloride-sensitive amine oxidase precursor |
| NP_000231.1 | amine oxidase [flavin-containing] A |
| NP_000889.3 | amine oxidase [flavin-containing] B |
| NP_001139748.1 | arylacetamide deacetylase-like 1 isoform a |
| NP_065843.3 | arylacetamide deacetylase-like 1 isoform b |
| NP_001139749.1 | arylacetamide deacetylase-like 1 isoform c |
| NP_001139750.1 | arylacetamide deacetylase-like 1 isoform c |
| NP_001096640.1 | arylacetamide deacetylase-like 3 isoform 1 |
| NP_000006.2 | arylamine N-acetyltransferase 2 |
| NP_059125.2 | beta,beta-carotene 15,15'-monooxygenase |
| NP_000172.2 | beta-glucuronidase precursor |
| NP_005434.4 | bifunctional 3'-phosphoadenosine 5'-phosphosulfate synthase 1 |
| NP_004661.2 | bifunctional 3'-phosphoadenosine 5'-phosphosulfate synthase 2 isoform a |
| NP_001015880.1 | bifunctional 3'-phosphoadenosine 5'-phosphosulfate synthase 2 isoform b |
| NP_003158.2 | bile salt sulfotransferase |
| NP_001748.1 | carbonyl reductase [NADPH] 1 |
| NP_001227.1 | carbonyl reductase [NADPH] 3 |
| NP_116172.2 | carbonyl reductase family member 4 |
| NP_003860.2 | carboxylesterase 2 isoform 1 |
| NP_932327.1 | carboxylesterase 2 isoform 2 |
| NP_079198.2 | carboxylesterase 3 precursor |
| NP_001137157.1 | carboxylesterase 7 isoform 1 |
| NP_659461.1 | carboxylesterase 7 isoform 2 |
| NP_776176.3 | carboxylesterase 8 |
| NP_055512.1 | carboxyl-terminal PDZ ligand of neuronal nitric oxide synthase protein isoform 1 |
| NP_001119532.2 | carboxyl-terminal PDZ ligand of neuronal nitric oxide synthase protein isoform 2 |
| NP_001158229.1 | carboxyl-terminal PDZ ligand of neuronal nitric oxide synthase protein isoform 3 |
| NP_001743.1 | catalase |
| NP_001128633.1 | catechol O-methyltransferase isoform MB-COMT |
| NP_001128634.1 | catechol O-methyltransferase isoform MB-COMT |
| NP_000745.1 | catechol O-methyltransferase isoform MB-COMT |
| NP_009294.1 | catechol O-methyltransferase isoform S-COMT |
| NP_653190.2 | catechol-O-methyltransferase domain containing 1 |
| NP_653190.2 | catechol-O-methyltransferase domain containing 1 |
| NP_000771.2 | cholesterol 7-alpha-monooxygenase |
| NP_001159976.1 | class IV alcohol dehydrogenase, mu or sigma subunit isoform 1 |
| NP_000664.2 | class IV alcohol dehydrogenase, mu or sigma subunit isoform 2 |
| NP_000488.3 | cytochrome P450 11B1, mitochondrial isoform 1 precursor |
| NP_001021384.1 | cytochrome P450 11B1, mitochondrial isoform 2 precursor |
| NP_000489.3 | cytochrome P450 11B2, mitochondrial precursor |
| NP_000094.2 | cytochrome P450 19A1 |
| NP_112503.1 | cytochrome P450 19A1 |
| NP_000490.1 | cytochrome P450 1A1 |
| NP_000752.2 | cytochrome P450 1A2 |
| NP_000095.2 | cytochrome P450 1B1 |
| NP_803882.1 | cytochrome P450 20A1 |
| NP_000774.2 | cytochrome P450 26A1 isoform 1 |
| NP_476498.1 | cytochrome P450 26A1 isoform 2 |
| NP_063938.1 | cytochrome P450 26B1 |
| NP_899230.2 | cytochrome P450 26C1 |
| NP_000757.2 | cytochrome P450 2A13 |
| NP_000753.3 | cytochrome P450 2A6 |
| NP_000755.2 | cytochrome P450 2A7 isoform 1 |
| NP_085079.2 | cytochrome P450 2A7 isoform 2 |
| NP_000758.1 | cytochrome P450 2B6 |
| NP_000763.1 | cytochrome P450 2C18 isoform 1 |
| NP_001122397.1 | cytochrome P450 2C18 isoform 2 |
| NP_000760.1 | cytochrome P450 2C19 |
| NP_000761.3 | cytochrome P450 2C8 |
| NP_000762.2 | cytochrome P450 2C9 |
| NP_000097.2 | cytochrome P450 2D6 isoform 1 |
| NP_001020332.1 | cytochrome P450 2D6 isoform 2 |
| NP_000764.1 | cytochrome P450 2E1 |
| NP_000765.2 | cytochrome P450 2F1 |
| NP_000766.2 | cytochrome P450 2J2 |
| NP_085125.1 | cytochrome P450 2S1 |
| NP_898898.1 | cytochrome P450 2U1 |
| NP_060251.2 | cytochrome P450 2W1 |
| NP_059488.2 | cytochrome P450 3A4 |
| NP_073731.1 | cytochrome P450 3A43 isoform 1 |
| NP_476436.1 | cytochrome P450 3A43 isoform 2 |
| NP_476437.1 | cytochrome P450 3A43 isoform 3 |
| NP_000768.1 | cytochrome P450 3A5 |
| NP_000756.2 | cytochrome P450 3A7 |
| NP_000769.2 | cytochrome P450 4A11 |
| NP_001093242.1 | cytochrome P450 4B1 isoform a |
| NP_000770.2 | cytochrome P450 4B1 isoform b |
| NP_076433.2 | cytochrome P450 4F12 |
| NP_775754.2 | cytochrome P450 4F22 |
| NP_009184.1 | cytochrome P450 4F8 |
| NP_828847.1 | cytochrome P450 4X1 |
| NP_835235.1 | cytochrome P450 4Z1 |
| NP_078790.2 | cytochrome P450, family 2, subfamily R, polypeptide 1 |
| NP_001001665.3 | cytochrome P450, family 27, subfamily C, polypeptide 1 |
| NP_001010969.2 | cytochrome P450, family 4, subfamily A, polypeptide 22 precursor |
| NP_067010.3 | cytochrome P450, family 4, subfamily F, polypeptide 11 |
| NP_001122404.1 | cytochrome P450, family 4, subfamily F, polypeptide 11 |
| NP_997235.3 | cytochrome P450, family 4, subfamily v, polypeptide 2 |
| NP_006659.1 | cytochrome P450, family 46 |
| NP_056344.2 | DBH-like monooxygenase protein 1 isoform 2 |
| NP_001075957.1 | dehydrogenase/reductase (SDR family) member 4 like 1 |
| NP_612461.1 | dehydrogenase/reductase SDR family member 1 |
| NP_001129522.1 | dehydrogenase/reductase SDR family member 1 |
| NP_077284.2 | dehydrogenase/reductase SDR family member 11 precursor |
| NP_001026889.1 | dehydrogenase/reductase SDR family member 12 isoform 1 |
| NP_078981.1 | dehydrogenase/reductase SDR family member 12 isoform 2 |
| NP_653284.2 | dehydrogenase/reductase SDR family member 13 precursor |
| NP_878912.1 | dehydrogenase/reductase SDR family member 2 isoform 1 |
| NP_005785.1 | dehydrogenase/reductase SDR family member 2 isoform 2 |
| NP_066284.2 | dehydrogenase/reductase SDR family member 4 |
| NP_932349.2 | dehydrogenase/reductase SDR family member 4-like 2 |
| NP_057113.1 | dehydrogenase/reductase SDR family member 7 precursor |
| NP_056325.2 | dehydrogenase/reductase SDR family member 7B |
| NP_001099041.1 | dehydrogenase/reductase SDR family member 7C |
| NP_005762.2 | dehydrogenase/reductase SDR family member 9 |
| NP_001135742.1 | dehydrogenase/reductase SDR family member 9 |
| NP_001135743.1 | dehydrogenase/reductase SDR family member 9 |
| NP_954674.1 | dehydrogenase/reductase SDR family member 9 |
| NP_660160.2 | dehydrogenase/reductase SDR family member on chromosome X precursor |
| NP_112594.1 | deoxyhypusine hydroxylase/monooxygenase |
| NP_001138637.1 | deoxyhypusine hydroxylase/monooxygenase |
| NP_002961.1 | diamine N-acetyltransferase 1 |
| NP_002012.1 | dimethylaniline monooxygenase [N-oxide-forming] 1 |
| NP_001451.1 | dimethylaniline monooxygenase [N-oxide-forming] 2 |
| NP_001002294.1 | dimethylaniline monooxygenase [N-oxide-forming] 3 |
| NP_008825.4 | dimethylaniline monooxygenase [N-oxide-forming] 3 |
| NP_002013.1 | dimethylaniline monooxygenase [N-oxide-forming] 4 |
| NP_001452.2 | dimethylaniline monooxygenase [N-oxide-forming] 5 isoform 1 |
| NP_001138301.1 | dimethylaniline monooxygenase [N-oxide-forming] 5 isoform 2 |
| NP_001138302.1 | dimethylaniline monooxygenase [N-oxide-forming] 5 isoform 3 |
| NP_000778.3 | dopamine beta-hydroxylase precursor |
| NP_004444.2 | electron transfer flavoprotein-ubiquinone oxidoreductase, mitochondrial precursor |
| NP_001500.1 | epididymal secretory glutathione peroxidase isoform 1 precursor |
| NP_003987.2 | epididymal secretory glutathione peroxidase isoform 2 precursor |
| NP_001129490.1 | epoxide hydrolase 1 |
| NP_000111.1 | epoxide hydrolase 1 |
| NP_001970.2 | epoxide hydrolase 2 |
| NP_005411.1 | estrogen sulfotransferase |
| NP_071417.2 | galactose-3-O-sulfotransferase 2 |
| NP_149025.1 | galactose-3-O-sulfotransferase 3 |
| NP_078913.3 | galactose-3-O-sulfotransferase 4 |
| NP_004852.1 | galactosylceramide sulfotransferase |
| NP_113610.2 | GalNAc-4-sulfotransferase 2 |
| NP_000812.2 | gamma-glutamyl carboxylase isoform 1 |
| NP_001135741.1 | gamma-glutamyl carboxylase isoform 2 |
| NP_001093251.1 | gamma-glutamyltransferase 5 isoform a |
| NP_004112.2 | gamma-glutamyltransferase 5 isoform b |
| NP_001093252.1 | gamma-glutamyltransferase 5 isoform c |
| NP_001116362.1 | gamma-glutamyltransferase 6 isoform a |
| NP_699169.2 | gamma-glutamyltransferase 6 isoform b |
| NP_821158.2 | gamma-glutamyltransferase 7 |
| NP_842563.1 | gamma-glutamyltransferase light chain 1 |
| NP_842564.1 | gamma-glutamyltransferase light chain 1 |
| NP_954578.2 | gamma-glutamyltransferase-like 4 isoform 1 |
| NP_005256.2 | gamma-glutamyltranspeptidase 1 precursor |
| NP_038347.2 | gamma-glutamyltranspeptidase 1 precursor |
| NP_001027536.1 | gamma-glutamyltranspeptidase 1 precursor |
| NP_001027537.1 | gamma-glutamyltranspeptidase 1 precursor |
| NP_932332.1 | glucosamine 6-phosphate N-acetyltransferase |
| NP_001489.1 | glutamate--cysteine ligase catalytic subunit |
| NP_002052.1 | glutamate--cysteine ligase regulatory subunit |
| NP_000572.2 | glutathione peroxidase 1 isoform 1 |
| NP_958799.1 | glutathione peroxidase 1 isoform 2 |
| NP_002074.2 | glutathione peroxidase 2 |
| NP_002075.2 | glutathione peroxidase 3 precursor |
| NP_874360.1 | glutathione peroxidase 6 precursor |
| NP_056511.2 | glutathione peroxidase 7 precursor |
| NP_665683.1 | glutathione S-transferase alpha 1 |
| NP_000837.3 | glutathione S-transferase alpha 2 |
| NP_000838.3 | glutathione S-transferase alpha 3 |
| NP_001503.1 | glutathione S-transferase alpha 4 |
| NP_714543.1 | glutathione S-transferase alpha 5 |
| NP_001026890.2 | glutathione S-transferase C-terminal domain-containing protein isoform 1 |
| NP_079027.2 | glutathione S-transferase C-terminal domain-containing protein isoform 2 |
| NP_057001.1 | glutathione S-transferase kappa 1 isoform a |
| NP_001137151.1 | glutathione S-transferase kappa 1 isoform b |
| NP_001137152.1 | glutathione S-transferase kappa 1 isoform c |
| NP_001137153.1 | glutathione S-transferase kappa 1 isoform d |
| NP_000552.2 | glutathione S-transferase mu 1 isoform 1 |
| NP_666533.1 | glutathione S-transferase mu 1 isoform 2 |
| NP_000839.1 | glutathione S-transferase mu 2 isoform 1 |
| NP_001135840.1 | glutathione S-transferase mu 2 isoform 2 |
| NP_000840.2 | glutathione S-transferase Mu 3 |
| NP_000841.1 | glutathione S-transferase mu 4 isoform 1 |
| NP_671489.1 | glutathione S-transferase mu 4 isoform 2 |
| NP_000842.2 | glutathione S-transferase mu 5 |
| NP_899062.1 | glutathione S-transferase omega 2 |
| NP_004823.1 | glutathione S-transferase omega-1 |
| NP_000843.1 | glutathione S-transferase P |
| NP_001074312.1 | glutathione S-transferase theta 2B |
| NP_000844.2 | glutathione S-transferase theta-1 |
| NP_000845.1 | glutathione S-transferase theta-2 |
| NP_000169.1 | glutathione synthetase |
| NP_665877.1 | glutathione transferase zeta 1 isoform 1 |
| NP_665878.2 | glutathione transferase zeta 1 isoform 2 |
| NP_001504.2 | glutathione transferase zeta 1 isoform 3 |
| NP_002037.2 | glyceraldehyde-3-phosphate dehydrogenase |
| NP_055179.1 | glyceraldehyde-3-phosphate dehydrogenase, spermatogenic |
| NP_653270.1 | glycosyltransferase 1 domain containing 1 |
| NP_078932.2 | glycosyltransferase 25 domain containing 1 precursor |
| NP_057258.3 | glycosyltransferase 25 family member 3 precursor |
| NP_892019.2 | glycosyltransferase 6 domain containing 1 |
| NP_112592.1 | glycosyltransferase 8 domain containing 2 |
| NP_775872.1 | glycosyltransferase 8 domain containing 3 isoform 1 |
| NP_001093120.1 | glycosyltransferase 8 domain containing 3 isoform 2 |
| NP_690909.1 | glycosyltransferase 8 domain-containing protein 1 |
| NP_060916.1 | glycosyltransferase 8 domain-containing protein 1 |
| NP_001010983.1 | glycosyltransferase 8 domain-containing protein 1 |
| NP_116195.2 | glycosyltransferase precursor |
| NP_001158101.1 | glycosyltransferase-like domain-containing protein 1 isoform a |
| NP_001006637.1 | glycosyltransferase-like domain-containing protein 1 isoform a |
| NP_078935.2 | glycosyltransferase-like domain-containing protein 1 isoform b |
| NP_004728.1 | glycosyltransferase-like protein LARGE1 |
| NP_598397.1 | glycosyltransferase-like protein LARGE1 |
| NP_689525.3 | glycosyltransferase-like protein LARGE2 |
| NP_849180.1 | hepatocyte nuclear factor 4-alpha isoform a |
| NP_000448.3 | hepatocyte nuclear factor 4-alpha isoform b |
| NP_849181.1 | hepatocyte nuclear factor 4-alpha isoform c |
| NP_787110.2 | hepatocyte nuclear factor 4-alpha isoform d |
| NP_001025174.1 | hepatocyte nuclear factor 4-alpha isoform e |
| NP_001025175.1 | hepatocyte nuclear factor 4-alpha isoform f |
| NP_008826.1 | histamine N-methyltransferase isoform 1 |
| NP_001019245.1 | histamine N-methyltransferase isoform 2 |
| NP_001019246.1 | histamine N-methyltransferase isoform 3 |
| NP_941994.1 | hydroxysteroid 11-beta-dehydrogenase 1-like protein isoform a |
| NP_941995.1 | hydroxysteroid 11-beta-dehydrogenase 1-like protein isoform b |
| NP_941996.1 | hydroxysteroid 11-beta-dehydrogenase 1-like protein isoform c |
| NP_941997.1 | hydroxysteroid 11-beta-dehydrogenase 1-like protein isoform d |
| NP_940935.1 | hydroxysteroid 11-beta-dehydrogenase 1-like protein isoform e |
| NP_941993.1 | hydroxysteroid 11-beta-dehydrogenase 1-like protein isoform f |
| NP_115679.2 | hydroxysteroid dehydrogenase like 2 |
| NP_113651.4 | inactive hydroxysteroid dehydrogenase-like protein 1 isoform a |
| NP_001139523.1 | inactive hydroxysteroid dehydrogenase-like protein 1 isoform b |
| NP_003928.1 | kynureninase isoform a |
| NP_001028170.1 | kynureninase isoform b |
| NP_003670.2 | kynurenine 3-monooxygenase |
| NP_006142.1 | lactoperoxidase isoform 1 preproprotein |
| NP_001153574.1 | lactoperoxidase isoform 3 preproprotein |
| NP_056238.2 | L-aminoadipate-semialdehyde dehydrogenase-phosphopantetheinyl transferase |
| NP_001020366.1 | liver carboxylesterase 1 isoform a precursor |
| NP_001020365.1 | liver carboxylesterase 1 isoform b precursor |
| NP_001257.4 | liver carboxylesterase 1 isoform c precursor |
| NP_001986.2 | long-chain-fatty-acid--CoA ligase 1 |
| NP_004449.1 | long-chain-fatty-acid--CoA ligase 4 isoform 1 |
| NP_055977.3 | long-chain-fatty-acid--CoA ligase ACSBG1 |
| NP_003725.1 | membrane primary amine oxidase precursor |
| NP_005580.1 | methylmalonate-semialdehyde dehydrogenase [acylating], mitochondrial precursor |
| NP_064696.1 | microsomal glutathione S-transferase 1 |
| NP_665707.1 | microsomal glutathione S-transferase 1 |
| NP_665734.1 | microsomal glutathione S-transferase 1 |
| NP_665735.1 | microsomal glutathione S-transferase 1 |
| NP_002404.1 | microsomal glutathione S-transferase 2 |
| NP_004519.1 | microsomal glutathione S-transferase 3 |
| NP_001153643.1 | N-acetyltransferase 1 isoform a |
| NP_001153644.1 | N-acetyltransferase 1 isoform a |
| NP_001153645.1 | N-acetyltransferase 1 isoform a |
| NP_001153646.1 | N-acetyltransferase 1 isoform a |
| NP_000653.3 | N-acetyltransferase 1 isoform a |
| NP_001153642.1 | N-acetyltransferase 1 isoform a |
| NP_001153651.1 | N-acetyltransferase 1 isoform a |
| NP_001153647.1 | N-acetyltransferase 1 isoform b |
| NP_001153648.1 | N-acetyltransferase 1 isoform b |
| NP_078938.2 | N-acetyltransferase 10 isoform a |
| NP_001137502.1 | N-acetyltransferase 10 isoform b |
| NP_079047.2 | N-acetyltransferase 11 |
| NP_079422.1 | N-acetyltransferase 13 |
| NP_065111.1 | N-acetyltransferase 14 |
| NP_001077069.1 | N-acetyltransferase 15 |
| NP_079121.1 | N-acetyltransferase 15 |
| NP_001077070.1 | N-acetyltransferase 15 |
| NP_036323.2 | N-acetyltransferase 6 |
| NP_848652.2 | N-acetyltransferase 8-like protein |
| NP_056469.2 | N-acetyltransferase 9 |
| NP_443143.2 | N-acetyltransferase ESCO1 |
| NP_001017420.1 | N-acetyltransferase ESCO2 |
| NP_001011713.2 | N-acetyltransferase MAK3 homolog |
| NP_000894.1 | NAD(P)H dehydrogenase [quinone] 1 isoform a |
| NP_001020604.1 | NAD(P)H dehydrogenase [quinone] 1 isoform b |
| NP_001020605.1 | NAD(P)H dehydrogenase [quinone] 1 isoform c |
| NP_004997.4 | NADH-ubiquinone oxidoreductase 75 kDa subunit, mitochondrial precursor |
| NP_077728.2 | NADPH:adrenodoxin oxidoreductase, mitochondrial isoform 1 precursor |
| NP_004101.2 | NADPH:adrenodoxin oxidoreductase, mitochondrial isoform 2 precursor |
| NP_000932.3 | NADPH--cytochrome P450 reductase |
| NP_004775.1 | N-deacetylase/N-sulfotransferase 3 |
| NP_006160.1 | nicotinamide N-methyltransferase |
| NP_057037.1 | nitric oxide synthase interacting protein |
| NP_443178.2 | nitric oxide synthase trafficker isoform 1 |
| NP_001034813.2 | nitric oxide synthase trafficker isoform 2 |
| NP_001165103.1 | nitric oxide synthase trafficker isoform 3 |
| NP_001165102.1 | nitric oxide synthase trafficker isoform 4 |
| NP_000611.1 | nitric oxide synthase, brain |
| NP_000594.2 | nitric oxide synthase, endothelial isoform 1 |
| NP_001153581.1 | nitric oxide synthase, endothelial isoform 2 |
| NP_001153582.1 | nitric oxide synthase, endothelial isoform 3 |
| NP_001153583.1 | nitric oxide synthase, endothelial isoform 4 |
| NP_000616.3 | nitric oxide synthase, inducible |
| NP_005117.3 | nuclear receptor subfamily 1 group D member 2 isoform 1 |
| NP_001138897.1 | nuclear receptor subfamily 1 group D member 2 isoform 2 |
| NP_001070941.1 | nuclear receptor subfamily 1 group I member 3 isoform 12 |
| NP_001070948.1 | nuclear receptor subfamily 1 group I member 3 isoform 2 |
| NP_009052.3 | nuclear receptor subfamily 1, group H, member 2 |
| NP_003880.3 | nuclear receptor subfamily 1, group I, member 2 isoform 1 |
| NP_071285.1 | nuclear receptor subfamily 1, group I, member 2 isoform 2 |
| NP_148934.1 | nuclear receptor subfamily 1, group I, member 2 isoform 3 |
| NP_003288.2 | nuclear receptor subfamily 2 group C member 1 isoform a |
| NP_001027458.1 | nuclear receptor subfamily 2 group C member 1 isoform b |
| NP_001120834.1 | nuclear receptor subfamily 2 group C member 1 isoform c |
| NP_003260.1 | nuclear receptor subfamily 2 group E member 1 |
| NP_005645.1 | nuclear receptor subfamily 2, group F, member 1 |
| NP_995582.1 | nuclear receptor subfamily 5 group A member 2 isoform 1 |
| NP_001480.3 | nuclear receptor subfamily 6 group A member 1 isoform 2 |
| NP_000910.2 | peptidyl-glycine alpha-amidating monooxygenase isoform a preproprotein |
| NP_620121.1 | peptidyl-glycine alpha-amidating monooxygenase isoform b preproprotein |
| NP_620176.1 | peptidyl-glycine alpha-amidating monooxygenase isoform c preproprotein |
| NP_620177.1 | peptidyl-glycine alpha-amidating monooxygenase isoform d preproprotein |
| NP_001170777.1 | peptidyl-glycine alpha-amidating monooxygenase isoform e preproprotein |
| NP_002565.1 | peroxiredoxin-1 |
| NP_859047.1 | peroxiredoxin-1 |
| NP_859048.1 | peroxiredoxin-1 |
| NP_005800.3 | peroxiredoxin-2 isoform a |
| NP_859428.1 | peroxiredoxin-2 isoform c |
| NP_006397.1 | peroxiredoxin-4 |
| NP_036226.1 | peroxiredoxin-5, mitochondrial isoform a precursor |
| NP_857634.1 | peroxiredoxin-5, mitochondrial isoform b precursor |
| NP_857635.1 | peroxiredoxin-5, mitochondrial isoform c precursor |
| NP_004896.1 | peroxiredoxin-6 |
| NP_002076.2 | phospholipid hydroperoxide glutathione peroxidase, mitochondrial isoform A precursor |
| NP_001034936.1 | phospholipid hydroperoxide glutathione peroxidase, mitochondrial isoform B precursor |
| NP_001034937.1 | phospholipid hydroperoxide glutathione peroxidase, mitochondrial isoform C precursor |
| NP_690875.1 | polyamine oxidase isoform 1 |
| NP_997010.1 | polyamine oxidase isoform 2 |
| NP_997011.1 | polyamine oxidase isoform 4 |
| NP_000952.1 | prostacyclin synthase |
| NP_000953.2 | prostaglandin G/H synthase 1 isoform 1 precursor |
| NP_542158.1 | prostaglandin G/H synthase 1 isoform 2 precursor |
| NP_000954.1 | prostaglandin G/H synthase 2 precursor |
| NP_005612.1 | protein S100-A12 |
| NP_003587.1 | protein-tyrosine sulfotransferase 1 |
| NP_003586.3 | protein-tyrosine sulfotransferase 2 |
| NP_001008566.1 | protein-tyrosine sulfotransferase 2 |
| NP_004872.2 | quinone oxidoreductase PIG3 |
| NP_671713.1 | quinone oxidoreductase PIG3 |
| NP_665857.2 | quinone oxidoreductase-like protein 1 |
| NP_001149.2 | retina-specific copper amine oxidase isoform a |
| NP_033720.2 | retina-specific copper amine oxidase isoform b |
| NP_001160051.1 | serotonin N-acetyltransferase isoform 1 |
| NP_001079.1 | serotonin N-acetyltransferase isoform 2 |
| NP_003120.2 | squalene monooxygenase |
| NP_000342.2 | steryl-sulfatase precursor |
| NP_733936.1 | succinate-semialdehyde dehydrogenase, mitochondrial isoform 1 precursor |
| NP_001071.1 | succinate-semialdehyde dehydrogenase, mitochondrial isoform 2 precursor |
| NP_067022.1 | sulfide:quinone oxidoreductase, mitochondrial precursor |
| NP_001046.2 | sulfotransferase 1A1 isoform a |
| NP_803565.1 | sulfotransferase 1A1 isoform a |
| NP_803566.1 | sulfotransferase 1A1 isoform a |
| NP_803878.1 | sulfotransferase 1A1 isoform a |
| NP_803880.1 | sulfotransferase 1A1 isoform b |
| NP_803564.1 | sulfotransferase 1A2 |
| NP_001045.1 | sulfotransferase 1A2 |
| NP_003157.1 | sulfotransferase 1A3/1A4 |
| NP_808220.1 | sulfotransferase 1A3/1A4 |
| NP_001017389.1 | sulfotransferase 1A3/1A4 |
| NP_001017390.1 | sulfotransferase 1A3/1A4 |
| NP_001047.1 | sulfotransferase 1C2 isoform a |
| NP_789795.1 | sulfotransferase 1C2 isoform b |
| NP_006579.2 | sulfotransferase 1C4 |
| NP_055166.1 | sulfotransferase 4A1 |
| NP_055280.2 | sulfotransferase family cytosolic 1B member 1 |
| NP_004596.2 | sulfotransferase family cytosolic 2B member 1 isoform a |
| NP_814444.1 | sulfotransferase family cytosolic 2B member 1 isoform b |
| NP_001008743.1 | sulfotransferase family, cytosolic, 1C, member 3 |
| NP_001027549.1 | sulfotransferase family, cytosolic, 6B, member 1 |
| NP_000358.1 | thiopurine S-methyltransferase |
| NP_003303.2 | thiosulfate sulfurtransferase |
| NP_001106678.1 | thiosulfate sulfurtransferase/rhodanese-like domain-containing protein 1 isoform 1 |
| NP_001106677.1 | thiosulfate sulfurtransferase/rhodanese-like domain-containing protein 1 isoform 2 |
| NP_001106676.1 | thiosulfate sulfurtransferase/rhodanese-like domain-containing protein 1 isoform 3 |
| NP_640339.4 | thiosulfate sulfurtransferase/rhodanese-like domain-containing protein 2 |
| NP_001124438.1 | thromboxane-A synthase isoform 1 |
| NP_001052.2 | thromboxane-A synthase isoform 1 |
| NP_112246.2 | thromboxane-A synthase isoform 2 |
| NP_001159725.1 | thromboxane-A synthase isoform 3 |
| NP_001159726.1 | thromboxane-A synthase isoform 4 |
| NP_000538.3 | thyroid peroxidase isoform a |
| NP_783650.1 | thyroid peroxidase isoform b |
| NP_783652.1 | thyroid peroxidase isoform d |
| NP_783653.1 | thyroid peroxidase isoform e |
| NP_055290.1 | trans-1,2-dihydrobenzene-1,2-diol dehydrogenase |
| NP_003395.1 | tyrosine 3-monooxygenase/tryptophan 5-monooxygenase activation protein, beta polypeptide |
| NP_647539.1 | tyrosine 3-monooxygenase/tryptophan 5-monooxygenase activation protein, beta polypeptide |
| NP_872282.1 | ubiquinone biosynthesis monooxygenase COQ6 isoform a |
| NP_872286.1 | ubiquinone biosynthesis monooxygenase COQ6 isoform b |
| NP_005706.1 | uronyl-2-sulfotransferase |
| NP_000370.2 | xanthine dehydrogenase/oxidase |
| NP_787103.1 | zinc-binding alcohol dehydrogenase domain-containing protein 2 |
