# Supplementary material for: Elucidation of Xenobiotic Metabolism Pathways in Human Skin and Human Skin Models by Proteomic Profiling
Source: PLoS One. 2012 Jul 26;7(7):e41721. doi: 10.1371/journal.pone.0041721 (PMC3406074; doi:10.1371/journal.pone.0041721)
Supplement: Table S4 — Summary of the proteomic data upon which protein identifications are based. Each protein or family of proteins identified is listed in alphabetical order and is accompanied by corresponding NCBI numbers. The subcellular fraction containing the majority of the peptides for each protein is shown (microsomal: M or cytosolic: C) as well as the Sf value, which indicates the certainty of the protein identification in each group of samples. Details of the peptide sequences and charge upon which protein identity is based are shown, and the quality of the match of MS data to these sequences is indicated by Xcorr values that are shown for each group of samples. A BLAST-type search for each peptide sequence was performed and where matches to other proteins occur there are indicated by corresponding NCBI reference numbers. (DOCX) [file pone.0041721.s005.docx]

|  |  |  | EpiDerm 1188 | EpiDerm 254 | Episkin | RHE | HaCaT | Whole skin | Liver |  |
| --- | --- | --- | --- | --- | --- | --- | --- | --- | --- | --- |
| Protein |  |  |  |  |  |  |  |  |  |  |
|  | Fraction |  | Sample max Sf |  |  |  |  |  |  |  |
|  | Sequence | Charge | Peptide max Xc |  |  |  |  |  |  | Proteins to which peptide also matches |
|  |  |  | Sample npeps |  |  |  |  |  |  |  |
| 3-hydroxyacyl-CoA dehydrogenase type-2, NP_004484.1, NP_001032900.1 | | | |  |  |  |  |  |  |  |
|  | C |  | 0.98 | 0.98 | 0.98 | 0.98 | 0.98 | 0.98 | 0.99 |  |
|  | DLAPIGIR | 2 | 2.06 | 2.23 | - | - | - | - | 2.02 | - |
|  | DVQTALALAK | 2 | 2.73 | 2.64 | - | - | - | - | 2.81 | - |
|  | GGIVGMTLPIAR | 2 | - | - | - | - | - | - | 3.95 | - |
|  | GLVAVITGGASGLGLATAER | 3 | 6.38 | 6.48 | 6.09 | 6.29 | 6.37 | 6.32 | 7.24 | - |
|  | GQTHTLEDFQR | 2 | 3.64 | 3.65 | 2.86 | 3.60 | - | - | 4.00 | - |
|  | GVIINTASVAAFEGQVGQAAYSASK | 3 | 6.78 | 6.60 | 6.62 | 5.57 | 6.87 | 6.21 | 7.70 | - |
|  | KGQTHTLEDFQR | 2 | 3.01 | 3.03 | - | 2.58 | - | - | 3.78 | - |
|  | KLGNNCVFAPADVTSEK | 3 | - | - | - | - | - | - | 3.6 | - |
|  | KLGNNCVFAPADVTSEKDVQTALALAK | 3 | - | - | - | - | - | - | 8.26 | - |
|  | LGDPAEYAHLVQAIIENPFLNGEVIR | 3 | 7.02 | 7.15 | 5.23 | 6.46 | 2.90 | 5.59 | 6.36 | - |
|  | LGNNCVFAPADVTSEKDVQTALALAK | 3 | 3.85 | - | - | 4.25 | - | 4.59 | 4.93 | - |
|  | LVAGEMGQNEPDQGGQR | 2 | 3.00 | - | - | - | - | - | 5.34 | - |
|  | LVGQGASAVLLDLPNSGGEAQAK | 2 | 6.77 | 6.64 | 6.55 | 6.19 | - | 6.80 | 7.02 | - |
|  | VCNFLASQVPFPSR | 2 | 4.82 | 4.19 | - | 4.12 | - | - | 4.64 | - |
|  | VDVAVNCAGIAVASK | 2 | - | - | - | - | 5.20 | - | 5.57 | - |
|  | VLDVNLMGTFNVIR | 2 | - | - | - | - | - | - | 4.50 | - |
|  | VMTIAPGLFGTPLLTSLPEK | 2 | - | - | - | - | - | 5.30 | 5.60 | - |
|  |  |  | 11 | 9 | 5 | 8 | 4 | 6 | 17 |  |
| 11-beta-hydroxysteroid dehydrogenase 1, NP_005516.1, NP_861420.1 | | | |  |  |  |  |  |  |  |
|  | M |  | - | - | - | - | - | - | 0.96 |  |
|  | AVSGIVHMQAAPK | 2 | - | - | - | - | - | - | 3.77 | - |
|  | EECALEIIK | 2 | - | - | - | - | - | - | 2.52 | - |
|  | EMAYHLAK | 2 | - | - | - | - | - | - | 2.18 | - |
|  | FALDGFFSSIR | 2 | - | - | - | - | - | - | 3.96 | - |
|  | ILEFLYSTSYNMDR | 2 | - | - | - | - | - | - | 5.00 | - |
|  | VAYPMVAAYSASK | 2 | - | - | - | - | - | - | 3.93 | - |
|  |  |  | 0 | 0 | 0 | 0 | 0 | 0 | 6 |  |
| 14-3-3 protein beta/alpha, NP_003395.1, NP_647539.1 | |  |  |  |  |  |  |  |  |  |
|  | C |  | 0.98 | 0.98 | 0.98 | 0.98 | 0.98 | 0.98 | 0.98 |  |
|  | AVTEQGHELSNEER | 2 | 4.91 | 4.96 | - | 4.83 | 4.39 | 4.54 | 4.93 | - |
|  | DNLTLWTSENQGDEGDAGEGEN | 2 | 2.97 | 2.15 | - | - | 2.45 | 4.11 | - | - |
|  | DSTLIMQLLR | 2 | 3.83 | 4.13 | 3.85 | 3.54 | 3.75 | 3.02 | 4.08 | - |
|  | EKIEAELQDICNDVLELLDK | 3 | 7.31 | 6.91 | 7.28 | 6.62 | 6.97 | - | 7.14 | - |
|  | EMQPTHPIR | 2 | - | - | - | - | - | - | 2.14 | - |
|  | IEAELQDICNDVLELLDK | 2 | 6.29 | 6.12 | 6.06 | 5.98 | 6.19 | 6.12 | 6.69 | - |
|  | KEMQPTHPIR | 3 | - | - | 2.94 | - | - | - | 3.68 | - |
|  | LAEQAER | 2 | 2.62 | 2.61 | 2.66 | 2.66 | 2.47 | 2.16 | 2.58 | - |
|  | LGLALNFSVFYYEILNSPEK | 2 | 4.12 | 2.64 | - | - | - | - | - | - |
|  | MKGDYFR | 2 | - | - | - | - | - | - | 2.31 | - |
|  | NLLSVAYK | 2 | 2.60 | 2.89 | 2.64 | 2.72 | 2.47 | 2.49 | 2.60 | - |
|  | QTTVSNSQQAYQEAFEISK | 2 | 5.64 | 5.89 | 5.65 | 5.88 | 6.25 | 5.92 | 6.09 | - |
|  | TAFDEAIAELDTLNEESYK | 2 | 6.46 | 6.38 | 6.42 | 6.27 | 6.22 | 6.46 | 6.62 | - |
|  | TAFDEAIAELDTLNEESYKDSTLIMQLLR | 3 | - | - | - | - | - | - | 6.49 | - |
|  | VFYLK | 1 | 1.68 | 1.61 | 1.58 | 1.57 | 1.52 | 1.56 | 1.6 | - |
|  | YDDMAAAMK | 2 | - | - | - | - | - | 2.31 | 3.54 | - |
|  | YLIPNATQPESK | 2 | 3.48 | 3.73 | 3.54 | 3.63 | 3.26 | 3.93 | 3.92 | - |
|  | YLSEVASGDNK | 2 | 3.82 | 3.81 | 3.35 | 3.60 | 3.68 | 3.77 | 3.73 | - |
|  |  |  | 13 | 13 | 11 | 11 | 12 | 12 | 16 |  |
| 17-beta-hydroxysteroid dehydrogenase 13, NP_835236.2, NP_001129702.1 | | | |  |  |  |  |  |  |  |
|  | M |  | - | - | - | - | - | - | 0.95 |  |
|  | ALLPSMMER | 2 | - | - | - | - | - | - | 2.34 | - |
|  | FAAVGFHR | 2 | - | - | - | - | - | - | 2.64 | - |
|  | GLTSELQALGK | 2 | - | - | - | - | - | - | 3.42 | - |
|  | GVEETAAECR | 2 | - | - | - | - | - | - | 4.03 | - |
|  | GVEETAAECRK | 2 | - | - | - | - | - | - | 3.14 | - |
|  | LVLWDINK | 2 | - | - | - | - | - | - | 2.66 | - |
|  | LWPVLETDEVVR | 2 | - | - | - | - | - | - | 4.17 | - |
|  | QTTYEFAK | 2 | - | - | - | - | - | - | 2.11 | - |
|  | RGVEETAAECR | 3 | - | - | - | - | - | - | 3.48 | - |
|  | SLIDGILTNK | 2 | - | - | - | - | - | - | 3.67 | - |
|  | SLIDGILTNKK | 2 | - | - | - | - | - | - | 3.11 | - |
|  | TSCLCPVFVNTGFTK | 2 | - | - | - | - | - | - | 5.23 | - |
|  |  |  | 0 | 0 | 0 | 0 | 0 | 0 | 12 |  |
| 17-beta-hydroxysteroid dehydrogenase type 6, NP_003716.2 | | |  |  |  |  |  |  |  |  |
|  | M |  | - | - | - | - | - | - | 0.96 |  |
|  | EIQHFGVK | 2 | - | - | - | - | - | - | 2.40 | - |
|  | GQTSDRLETVTLDVTK | 3 | - | - | - | - | - | - | 3.57 | - |
|  | ISIVEPGYFR | 2 | - | - | - | - | - | - | 3.70 | - |
|  | IVNVSSILGR | 2 | - | - | - | - | - | - | 4.20 | - |
|  | LETVTLDVTK | 2 | - | - | - | - | - | - | 2.54 | - |
|  | MESIAAATQWVK | 2 | - | - | - | - | - | - | 4.83 | - |
|  | QVVSHLQDK | 2 | - | - | - | - | - | - | 2.65 | - |
|  | TGMTNMTQSLER | 2 | - | - | - | - | - | - | 4.08 | - |
|  | VAFFVGGYCVSK | 2 | - | - | - | - | - | - | 4.10 | - |
|  | VLAACLTEK | 2 | - | - | - | - | - | - | 3.65 | - |
|  | YGVEAFSDILR | 2 | - | - | - | - | - | - | 4.33 | - |
|  | YSAGWDAK | 2 | - | - | - | - | - | - | 2.89 | - |
|  |  |  | 0 | 0 | 0 | 0 | 0 | 0 | 12 |  |
| N-acetyltransferase 10, NP_001137502.1, NP_078938.2 | |  |  |  |  |  |  |  |  |  |
|  | C |  | - | - | 0.95 | - | 0.94 | - | - |  |
|  | EELEALFLPYDLK | 2 | - | - | 4.05 | - | 4.21 | - | - | - |
|  | GFDALQYQEHLDYEIIQSLNPEFNK | 3 | - | - | 3.92 | - | 4.67 | - | - | - |
|  | LGQAELVVIDEAAAIPLPLVK | 3 | - | - | - | - | 4.23 | - | - | - |
|  | VLETPQEIHTVSSEAVSLLEEVITPR | 3 | - | - | - | - | 3.30 | - | - | - |
|  |  |  | 0 | 0 | 2 | 0 | 4 | 0 | 0 |  |
| alcohol dehydrogenase 1A, NP_000658.1 | |  |  |  |  |  |  |  |  |  |
|  | C |  | - | - | - | - | - | 0.98 | 0.98 |  |
|  | AAVLWELK | 1 | - | - | - | - | - | - | 1.91 | NP_000660.1 |
|  | AKELGATECINPQDYK | 2 | - | - | - | - | - | 4.35 | 5.98 | NP_000660.1, NP_000659.2 |
|  | ELGATECINPQDYK | 2 | - | - | - | - | - | 4.85 | 5.15 | NP_000660.1, NP_000659.2 |
|  | EMTDGGVDFSFEVIGR | 2 | - | - | - | - | - | 3.20 | 4.40 | NP_000660.1, NP_000659.2 |
|  | FSLDALITHVLPFEK | 2 | - | - | - | - | - | 3.46 | 4.84 | NP_000659.2 |
|  | GAILGGFK | 1 | - | - | - | - | - | - | 2.11 | - |
|  | IDAASPLEK | 2 | - | - | - | - | - | 2.53 | 2.85 | NP_000660.1, NP_000659.2 |
|  | IIAVDINK | 2 | - | - | - | - | - | 2.17 | 2.81 | NP_000660.1, NP_000659.2 |
|  | IIAVDINKDK | 2 | - | - | - | - | - | 2.36 | 3.23 | NP_000660.1, NP_000659.2 |
|  | INEGFDLLHSGK | 2 | - | - | - | - | - | 3.52 | 4.22 | NP_000659.2 |
|  | KFSLDALITHVLPFEK | 3 | - | - | - | - | - | 4.04 | 5.91 | NP_000659.2 |
|  | KPFSIEEVEVAPPK | 2 | - | - | - | - | - | 2.92 | 5.05 | NP_000660.1 |
|  | KPIQEVLK | 2 | - | - | - | - | - | 3.02 | 3.41 | NP_000660.1, NP_000659.2 |
|  | LVADFMAK | 2 | - | - | - | - | - | 2.23 | 2.67 | NP_000660.1, NP_000659.2 |
|  | NDVSNPQGTLQDGTSR | 2 | - | - | - | - | - | 2.14 | 5.73 | - |
|  | NPESNYCLK | 2 | - | - | - | - | - | 3.13 | 3.08 | NP_000660.1, NP_000659.2 |
|  | VCLIGCGFSTGYGSAVNVAK | 2 | - | - | - | - | - | 6.78 | 7.29 | NP_000659.2 |
|  | VIPLAIPQCGK | 2 | - | - | - | - | - | - | 2.95 | - |
|  | VTPGSTCAVFGLGGVGLSAIMGCK | 2 | - | - | - | - | - | 5.58 | 6.71 | - |
|  |  |  | 0 | 0 | 0 | 0 | 0 | 16 | 19 |  |
| alcohol dehydrogenase 1B, NP_000659.2 | |  |  |  |  |  |  |  |  |  |
|  | C |  | - | - | - | - | - | 0.98 | 0.98 |  |
|  | AAVLWEVK | 2 | - | - | - | - | - | 3.32 | 3.18 | - |
|  | AKELGATECINPQDYK | 2 | - | - | - | - | - | 4.35 | 5.98 | NP_000658.1, NP_000660.1 |
|  | AYEVR | 1 | - | - | - | - | - | - | 1.73 | - |
|  | ELGATECINPQDYK | 2 | - | - | - | - | - | 4.81 | 5.15 | NP_000658.1, NP_000660.1 |
|  | EMTDGGVDFSFEVIGR | 2 | - | - | - | - | - | 3.20 | 4.40 | NP_000658.1, NP_000660.1 |
|  | FSLDALITHVLPFEK | 2 | - | - | - | - | - | 2.24 | 4.84 | NP_000658.1 |
|  | GAVYGGFK | 2 | - | - | - | - | - | - | 2.53 | - |
|  | GKPIHHFLGTSTFSQYTVVDENAVAK | 3 | - | - | - | - | - | - | 7.64 | - |
|  | GTLQDGTR | 2 | - | - | - | - | - | 2.31 | 2.41 | NP_000660.1 |
|  | IDAASPLEK | 2 | - | - | - | - | - | 2.53 | 2.85 | NP_000658.1, NP_000660.1 |
|  | IIAVDINK | 2 | - | - | - | - | - | 2.17 | 2.81 | NP_000658.1, NP_000660.1 |
|  | IIAVDINKDK | 2 | - | - | - | - | - | 2.36 | 3.23 | NP_000658.1, NP_000660.1 |
|  | INEGFDLLHSGK | 2 | - | - | - | - | - | 3.52 | 4.22 | NP_000658.1 |
|  | KFSLDALITHVLPFEK | 3 | - | - | - | - | - | 4.04 | 5.91 | NP_000658.1 |
|  | KPFSIEDVEVAPPK | 2 | - | - | - | - | - | 4.79 | 5.24 | - |
|  | KPIQEVLK | 2 | - | - | - | - | - | 3.02 | 3.41 | NP_000658.1, NP_000660.1 |
|  | LVADFMAK | 2 | - | - | - | - | - | 2.23 | 2.67 | NP_000658.1, NP_000660.1 |
|  | NDLGNPR | 1 | - | - | - | - | - | - | 1.75 | NP_000660.1 |
|  | NPESNYCLK | 2 | - | - | - | - | - | 3.13 | 3.08 | NP_000658.1, NP_000660.1 |
|  | VCKNPESNYCLK | 2 | - | - | - | - | - | 2.62 | 3.55 | - |
|  | VCLIGCGFSTGYGSAVNVAK | 2 | - | - | - | - | - | 6.78 | 7.29 | NP_000658.1 |
|  | VIPLFTPQCGK | 2 | - | - | - | - | - | 2.47 | 2.85 | NP_000660.1 |
|  | VTPGSTCAVFGLGGVGLSAVMGCK | 2 | - | - | - | - | - | 4.34 | 6.90 | NP_000661.2 |
|  |  |  | 0 | 0 | 0 | 0 | 0 | 19 | 23 |  |
| alcohol dehydrogenase 1C, NP_000660.1 | |  |  |  |  |  |  |  |  |  |
|  | C |  | - | - | - | - | - | 0.95 | 0.98 |  |
|  | AAVLWELK | 1 | - | - | - | - | - | - | 1.91 | NP_000658.1 |
|  | AKELGATECINPQDYK | 2 | - | - | - | - | - | 4.35 | 5.98 | NP_000658.1, NP_000659.2 |
|  | ELGATECINPQDYK | 2 | - | - | - | - | - | 4.85 | 5.15 | NP_000658.1, NP_000659.2 |
|  | EMTDGGVDFSFEVIGR | 2 | - | - | - | - | - | 3.20 | 4.40 | NP_000658.1, NP_000659.2 |
|  | FSLDALITNILPFEK | 2 | - | - | - | - | - | 2.38 | 4.45 | - |
|  | GAIFGGFK | 1 | - | - | - | - | - | - | 2.25 | - |
|  | GTLQDGTR | 2 | - | - | - | - | - | 2.31 | 2.41 | NP_000659.2 |
|  | IDAASPLEK | 2 | - | - | - | - | - | 2.53 | 2.85 | NP_000658.1, NP_000659.2 |
|  | IIAVDINK | 2 | - | - | - | - | - | 2.17 | 2.81 | NP_000658.1, NP_000659.2 |
|  | IIAVDINKDK | 2 | - | - | - | - | - | 2.36 | 3.23 | NP_000658.1, NP_000659.2 |
|  | INEGFDLLR | 2 | - | - | - | - | - | - | 3.48 | - |
|  | KFSLDALITNILPFEK | 2 | - | - | - | - | - | - | 5.62 | - |
|  | KPFSIEEVEVAPPK | 2 | - | - | - | - | - | 2.92 | 5.05 | NP_000658.1 |
|  | KPIQEVLK | 2 | - | - | - | - | - | 3.02 | 3.41 | NP_000658.1, NP_000659.2 |
|  | LVADFMAK | 2 | - | - | - | - | - | 2.23 | 2.67 | NP_000658.1, NP_000659.2 |
|  | NDLGNPR | 1 | - | - | - | - | - | - | 1.75 | NP_000659.2 |
|  | NPESNYCLK | 2 | - | - | - | - | - | 3.13 | 3.08 | NP_000658.1, NP_000659.2 |
|  | VCLIGCGFSTGYGSAVK | 2 | - | - | - | - | - | - | 6.36 | - |
|  | VCLIGCGFSTGYGSAVKVAK | 3 | - | - | - | - | - | - | 4.37 | - |
|  | VIPLFTPQCGK | 2 | - | - | - | - | - | 2.47 | 2.85 | NP_000659.2 |
|  | VTPGSTCAVFGLGGVGLSVVMGCK | 2 | - | - | - | - | - | - | 6.28 | NP_001095940.1, NP_000663.1 |
|  |  |  | 0 | 0 | 0 | 0 | 0 | 13 | 21 |  |
| alcohol dehydrogenase 4, NP_000661.2 | |  |  |  |  |  |  |  |  |  |
|  | C |  | - | - | - | - | - | 0.97 | 0.99 |  |
|  | AAIAWEAGKPLCIEEVEVAPPK | 2 | - | - | - | - | - | - | 6.61 | - |
|  | AALDCTTAGWGSCTFIGVAAGSK | 2 | - | - | - | - | - | - | 5.87 | - |
|  | ALGATDCLNPR | 2 | - | - | - | - | - | - | 3.77 | - |
|  | DLHKPIQEVIIELTK | 3 | - | - | - | - | - | 3.54 | 5.66 | - |
|  | FCLSPLTNLCGK | 2 | - | - | - | - | - | 2.47 | 4.33 | - |
|  | FEGLAFPVIVGHEAAGIVESIGPGVTNVKPGDK | 3 | - | - | - | - | - | 6.25 | 7.15 | - |
|  | FNLDALVTHTLPFDK | 2 | - | - | - | - | - | 3.94 | 5.11 | - |
|  | GGVDFALDCAGGSETMK | 2 | - | - | - | - | - | - | 6.34 | - |
|  | GLTIFPEELIIGR | 2 | - | - | - | - | - | 3.17 | 4.23 | - |
|  | IDDDANLER | 2 | - | - | - | - | - | - | 3.35 | - |
|  | IDDDANLERVCLLGCGFSTGYGAAINNAK | 3 | - | - | - | - | - | - | 3.03 | - |
|  | IIGIDINSEK | 2 | - | - | - | - | - | - | 3.38 | - |
|  | IQIIATSLCHTDATVIDSK | 3 | - | - | - | - | - | - | 3.98 | - |
|  | ISEAFDLMNQGK | 2 | - | - | - | - | - | - | 5.30 | - |
|  | KFNLDALVTHTLPFDK | 3 | - | - | - | - | - | - | 5.10 | - |
|  | LVTDYK | 1 | - | - | - | - | - | - | 2.01 | - |
|  | PLCIEEVEVAPPK | 2 | - | - | - | - | - | - | 3.56 | - |
|  | SPASDQQLMEDK | 2 | - | - | - | - | - | - | 4.81 | - |
|  | SVDSIPK | 1 | - | - | - | - | - | - | 1.84 | - |
|  | TINGTFFGGWK | 2 | - | - | - | - | - | - | 3.22 | - |
|  | VCLLGCGFSTGYGAAINNAK | 2 | - | - | - | - | - | - | 7.57 | - |
|  | VIPLYAPLCR | 2 | - | - | - | - | - | - | 2.56 | - |
|  | VTPGSTCAVFGLGGVGLSAVMGCK | 2 | - | - | - | - | - | 6.78 | 6.90 | NP_000659.2 |
|  |  |  | 0 | 0 | 0 | 0 | 0 | 6 | 23 |  |
| alcohol dehydrogenase 6, NP_001095940.1, NP_000663.1 | |  |  |  |  |  |  |  |  |  |
|  | C |  | - | - | - | - | - | - | 0.99 |  |
|  | AAILWKPGAPFSIEEVEVAPPK | 2 | - | - | - | - | - | - | 5.09 | - |
|  | AQELGATECLNPQDLK | 2 | - | - | - | - | - | - | 6.03 | - |
|  | EISVAK | 1 | - | - | - | - | - | - | 1.78 | - |
|  | GSVFGGWK | 2 | - | - | - | - | - | - | 2.51 | - |
|  | HLDLLYPTILGHEGAGIVESIGEGVSTVKPGDK | 3 | - | - | - | - | - | - | 6.34 | - |
|  | IDAVAPLEK | 2 | - | - | - | - | - | - | 3.25 | - |
|  | IIGVDVNK | 2 | - | - | - | - | - | - | 2.59 | - |
|  | IIGVDVNKEK | 2 | - | - | - | - | - | - | 3.05 | - |
|  | INEAVELMK | 2 | - | - | - | - | - | - | 2.85 | - |
|  | ISGQLFFSGR | 2 | - | - | - | - | - | - | 3.97 | - |
|  | KAQELGATECLNPQDLK | 3 | - | - | - | - | - | - | 5.14 | - |
|  | LNLDPLITHTLNLDK | 2 | - | - | - | - | - | - | 4.70 | - |
|  | LVADYMAEK | 2 | - | - | - | - | - | - | 3.19 | - |
|  | PGAPFSIEEVEVAPPK | 2 | - | - | - | - | - | - | 5.78 | - |
|  | SIYHFGNTSTFCEYTVIK | 3 | - | - | - | - | - | - | 4.06 | - |
|  | TQLMSDGTSR | 2 | - | - | - | - | - | - | 3.00 | - |
|  | VCLISCGFSTGFGAAINTAK | 2 | - | - | - | - | - | - | 7.37 | - |
|  | VTPGSTCAVFGLGGVGLSVVMGCK | 3 | - | - | - | - | - | - | 3.47 | NP_000660.1 |
|  | VVATGLCGTEMK | 2 | - | - | - | - | - | - | 4.56 | - |
|  |  |  | 0 | 0 | 0 | 0 | 0 | 0 | 19 |  |
| alcohol dehydrogenase class-3, NP_000662.3 | |  |  |  |  |  |  |  |  |  |
|  | C |  | 0.61 | 0.81 | 0.81 | 0.84 | 0.66 | 0.90 | 0.98 |  |
|  | AAVAWEAGKPLSIEEIEVAPPK | 3 | 3.00 | - | - | 2.58 | 3.80 | 2.54 | - | - |
|  | AGDTVIPLYIPQCGECK | 2 | 3.00 | 3.37 | - | - | - | 4.34 | - | - |
|  | IDPLAPLDK | 2 | 2.13 | 2.20 | 2.43 | 2.50 | - | 2.10 | 2.18 | - |
|  | VCLLGCGISTGYGAAVNTAK | 2 | - | - | - | - | - | - | 7.30 | - |
|  | VDEFVTHNLSFDEINK | 3 | - | 3.91 | 4.44 | 4.08 | 3.89 | 4.71 | - | - |
|  | VDEFVTHNLSFDEINKAFELMHSGKSIR | 3 | 2.55 | - | - | - | - | - | - | - |
|  |  |  | 4 | 3 | 2 | 3 | 2 | 4 | 2 |  |
| aldehyde dehydrogenase 1A1, NP_000680.2 | |  |  |  |  |  |  |  |  |  |
|  | C |  | 0.51 | - | - | - | 0.97 | 0.96 | 0.98 |  |
|  | ANNTFYGLSAGVFTK | 2 | - | - | - | - | 4.96 | - | 5.86 | - |
|  | EEIFGPVQQIMK | 2 | - | - | - | - | - | - | 2.49 | - |
|  | ELGEYGFHEYTEVK | 2 | - | - | - | - | - | - | 4.20 | - |
|  | GYFVQPTVFSNVTDEMR | 2 | - | - | - | - | - | - | 3.59 | - |
|  | IFVEESIYDEFVR | 2 | - | - | - | - | 4.42 | 4.31 | 5.20 | - |
|  | IGPALSCGNTVVVK | 2 | - | - | - | - | - | - | 4.28 | - |
|  | IGPALSCGNTVVVKPAEQTPLTALHVASLIK | 3 | - | - | - | - | - | - | 6.76 | - |
|  | ILDLIESGKK | 2 | - | - | - | - | - | - | 3.64 | - |
|  | KFPVFNPATEEELCQVEEGDK | 3 | - | - | - | - | - | - | 4.67 | - |
|  | KFPVFNPATEEELCQVEEGDKEDVDK | 3 | - | - | - | - | - | 6.13 | 6.13 | - |
|  | LADLIER | 2 | 2.32 | - | - | - | 2.11 | - | 2.43 | NP_000681.2 |
|  | LYSNAYLNDLAGCIK | 2 | - | - | - | - | 5.01 | 4.61 | 6.09 | - |
|  | RVTLELGGK | 2 | - | - | - | - | - | - | 3.36 | NP_000683.3, NP_000681.2 |
|  | SLDDVIK | 1 | - | - | - | - | - | - | 2.02 | - |
|  | SLDDVIKR | 2 | - | - | - | - | - | - | 2.89 | - |
|  | TIPIDGNFFTYTR | 2 | - | - | - | - | - | 2.76 | 3.03 | - |
|  | VAFTGSTEVGK | 2 | - | - | - | - | 2.73 | - | 3.31 | - |
|  | VTLELGGK | 2 | 2.02 | - | - | - | - | - | 2.23 | NP_000686.2, NP_001026786.1, NP_001128639.1, NP_001128640.1, NP_000687.3, NP_000682.3, NP_001026976.1, NP_000683.3, NP_000681.2, NP_000373.1 |
|  | YILGNPLTPGVTQGPQIDK | 2 | - | - | - | - | - | - | 5.74 | - |
|  | YILGNPLTPGVTQGPQIDKEQYDK | 2 | - | - | - | - | 3.14 | 3.09 | 4.66 | - |
|  |  |  | 2 | 0 | 0 | 0 | 6 | 5 | 20 |  |
| aldehyde dehydrogenase 1L1, NP_036322.2 | |  |  |  |  |  |  |  |  |  |
|  | C |  | - | - | - | - | - | 0.88 | 0.98 |  |
|  | ADPLGLEAEK | 2 | - | - | - | - | - | - | 3.37 | - |
|  | ADPLGLEAEKDGVPVFK | 2 | - | - | - | - | - | - | 4.25 | - |
|  | AGLILFGNDDK | 2 | - | - | - | - | - | - | 3.76 | - |
|  | AKGQALPDVVAK | 2 | - | - | - | - | - | - | 3.93 | - |
|  | ANATEFGLASGVFTR | 2 | - | - | - | - | - | - | 5.46 | - |
|  | DLGEAALNEYLR | 2 | - | - | - | - | - | - | 5.00 | - |
|  | ECEVLPDDTVSTLYNR | 2 | - | - | - | - | - | - | 5.27 | - |
|  | EGATLVCGGNQVPR | 2 | - | - | - | - | - | - | 3.73 | - |
|  | FADGDLDAVLSR | 2 | - | - | - | - | - | - | 4.70 | - |
|  | FAELTLK | 2 | - | - | - | - | - | - | 2.70 | - |
|  | FLFPEGIK | 2 | - | - | - | - | - | - | 2.34 | - |
|  | GAASSVLELTEAELVTAEAVR | 2 | - | - | - | - | - | 4.26 | 6.24 | - |
|  | GASAINWTLIHGDKK | 2 | - | - | - | - | - | - | 3.00 | - |
|  | GENCIAAGR | 2 | - | - | - | - | - | - | 3.51 | - |
|  | GQALPDVVAK | 2 | - | - | - | - | - | - | 2.45 | - |
|  | HGSIIYHPSLLPR | 2 | - | - | - | - | - | - | 3.06 | - |
|  | IAVIGQSLFGQEVYCHLR | 2 | - | - | - | - | - | - | 5.06 | - |
|  | IGFTGSTEVGK | 2 | - | - | - | - | - | - | 3.64 | - |
|  | INWDQPAEAIHNWIR | 2 | - | - | - | - | - | - | 4.37 | - |
|  | IQGSTIPINQAR | 2 | - | - | - | - | - | - | 3.91 | - |
|  | IQGSTIPINQARPNR | 2 | - | - | - | - | - | - | 2.51 | - |
|  | KEGHEVVGVFTVPDKDGK | 2 | - | - | - | - | - | - | 2.08 | - |
|  | KIGFTGSTEVGK | 2 | - | - | - | - | - | - | 3.83 | - |
|  | LADLMEQHQEELATIEALDAGAVYTLALK | 3 | - | - | - | - | - | 3.38 | 3.13 | - |
|  | LFVEDSIHDEFVR | 2 | - | - | - | - | - | - | 4.42 | - |
|  | LIAEGK | 1 | - | - | - | - | - | - | 1.74 | - |
|  | LPQPEEGATYEGIQK | 2 | - | - | - | - | - | - | 5.19 | - |
|  | LPQPEEGATYEGIQKK | 2 | - | - | - | - | - | - | 4.55 | - |
|  | LQAGTVFVNTYNK | 2 | - | - | - | - | - | - | 4.83 | - |
|  | LSDHPDVR | 2 | - | - | - | - | - | - | 2.61 | - |
|  | LTFFNSTLNTSGLVPEGDALPIPGAHRPGVVTK | 3 | - | - | - | - | - | 5.17 | 5.44 | - |
|  | LVEEVK | 1 | - | - | - | - | - | - | 2.08 | - |
|  | MILASNFFK | 2 | - | - | - | - | - | - | 3.13 | - |
|  | NIQLEDGK | 1 | - | - | - | - | - | - | 2.70 | - |
|  | RVVEEVR | 2 | - | - | - | - | - | - | 2.72 | - |
|  | SCAISNVK | 1 | - | - | - | - | - | - | 2.12 | - |
|  | SPLIIFADCDLNK | 2 | - | - | - | - | - | - | 4.94 | - |
|  | TAACLAAGNTVVIKPAQVTPLTALK | 3 | - | - | - | - | - | - | 4.34 | - |
|  | TDVAAPFGGFK | 2 | - | - | - | - | - | - | 3.26 | - |
|  | TSETINPTDGSVICQVSLAQVTDVDK | 2 | - | - | - | - | - | - | 6.23 | - |
|  | TSETINPTDGSVICQVSLAQVTDVDKAVAAAK | 3 | - | - | - | - | - | - | 2.76 | - |
|  | TVTFEY | 1 | - | - | - | - | - | - | 1.68 | - |
|  | VLEVEDSTDFFK | 2 | - | - | - | - | - | - | 4.57 | - |
|  | YFAGWCDK | 2 | - | - | - | - | - | - | 2.73 | - |
|  |  |  | 0 | 0 | 0 | 0 | 0 | 3 | 44 |  |
| aldehyde dehydrogenase 2, NP_000681.2 | |  |  |  |  |  |  |  |  |  |
|  | C |  | 0.97 | 0.98 | 0.94 | 0.97 | 0.96 | 0.96 | 0.98 |  |
|  | AAFQLGSPWR | 2 | - | - | - | 3.25 | - | - | - | - |
|  | ANNSTYGLAAAVFTK | 2 | 5.08 | 5.26 | - | 4.73 | 4.23 | 3.88 | 4.44 | - |
|  | EAGFPPGVVNIVPGFGPTAGAAIASHEDVDK | 3 | 4.29 | 3.90 | 3.71 | 3.50 | 3.14 | - | 3.69 | - |
|  | ELGEYGLQAYTEVK | 2 | 4.72 | 4.26 | - | - | 2.57 | 3.58 | 5.16 | - |
|  | GYFIQPTVFGDVQDGMTIAK | 2 | - | - | - | - | - | - | 2.65 | - |
|  | ILGYINTGK | 2 | - | - | - | - | - | - | 2.89 | - |
|  | KILGYINTGK | 2 | - | - | - | - | - | - | 3.38 | - |
|  | KTFPTVNPSTGEVICQVAEGDKEDVDK | 3 | 6.34 | 5.89 | 5.58 | 5.93 | - | - | 7.97 | - |
|  | LADLIER | 2 | 2.35 | - | - | 2.15 | 2.11 | - | 2.43 | NP_000680.2 |
|  | LGPALATGNVVVMK | 2 | 3.80 | - | - | 3.73 | - | - | - | - |
|  | LLCGGGIAADR | 2 | 3.17 | 3.08 | - | 2.89 | - | 3.31 | 4.04 | - |
|  | RVTLELGGK | 2 | - | - | - | - | - | - | 3.36 | NP_000683.3, NP_000680.2 |
|  | TEQGPQVDETQFK | 2 | 4.73 | 4.08 | - | 4.54 | - | 4.72 | 4.77 | - |
|  | TEQGPQVDETQFKK | 2 | - | - | - | - | - | - | 4.20 | - |
|  | TFPTVNPSTGEVICQVAEGDKEDVDK | 2 | - | - | - | - | - | - | 5.13 | - |
|  | TFVQEDIYDEFVER | 2 | 5.51 | 5.66 | 2.27 | 4.84 | 2.52 | - | 5.93 | - |
|  | TIEEVVGR | 2 | 2.52 | 2.10 | - | - | - | - | 3.03 | - |
|  | TIPIDGDFFSYTR | 2 | 2.45 | 3.56 | 2.63 | 2.92 | - | - | 2.88 | - |
|  | VAEQTPLTALYVANLIK | 2 | 5.48 | 5.86 | 5.13 | 5.38 | 5.05 | 5.90 | 5.93 | - |
|  | VAFTGSTEIGR | 2 | 3.43 | 3.28 | - | - | - | - | 4.00 | - |
|  | VIQVAAGSSNLK | 2 | 3.49 | - | 2.23 | - | - | 4.47 | 4.99 | - |
|  | VIQVAAGSSNLKR | 2 | 2.82 | - | - | 2.62 | - | - | 4.05 | - |
|  | VTLELGGK | 2 | 2.02 | - | - | - | - | - | 2.23 | NP_000686.2, NP_001026786.1, NP_001128639.1, NP_001128640.1, NP_000687.3, NP_000682.3, NP_001026976.1, NP_000683.3, NP_000373.1, NP_000680.2 |
|  | VVGNPFDSK | 2 | 2.43 | - | - | - | - | 2.30 | 2.68 | - |
|  |  |  | 17 | 11 | 6 | 12 | 6 | 7 | 22 |  |
| aldehyde dehydrogenase 3A2, NP_001026976.1, NP_000373.1 | | |  |  |  |  |  |  |  |  |
|  | M |  | - | - | 0.94 | - | - | 0.95 | 0.97 |  |
|  | DILTAIAADLCK | 2 | - | - | - | - | - | - | 4.65 | - |
|  | EFYGENIK | 2 | - | - | - | - | - | - | 2.05 | - |
|  | EFYGENIKESPDYER | 3 | - | - | - | - | - | - | 2.75 | - |
|  | EKDILTAIAADLCK | 2 | - | - | 3.28 | - | - | - | 5.28 | - |
|  | FDHIFYTGNTAVGK | 3 | - | - | 2.98 | - | - | - | 4.10 | - |
|  | HLTPVTLELGGK | 3 | - | - | - | - | - | - | 4.34 | NP_000686.2, NP_001026786.1, NP_001128639.1, NP_001128640.1, NP_000682.3 |
|  | HSFDTFSHQR | 3 | - | - | - | - | - | - | 3.70 | - |
|  | IAFGGETDEATR | 2 | - | - | 2.69 | - | - | 3.90 | 4.30 | - |
|  | ILSLLEGQK | 2 | - | - | - | - | - | - | 2.91 | - |
|  | IVMEAAAK | 2 | - | - | - | - | - | - | 2.59 | - |
|  | LQQLEALR | 2 | - | - | - | - | - | - | 2.46 | - |
|  | NVDEAINFINER | 2 | - | - | - | - | - | 3.72 | 4.62 | - |
|  | SPCYIDKDCDLDIVCR | 3 | - | - | - | - | - | - | 5.06 | - |
|  | VMQEEIFGPILPIVPVK | 3 | - | - | - | - | - | 4.25 | 5.59 | - |
|  | YIAPTVLTDVDPK | 2 | - | - | 3.81 | - | - | 3.19 | 4.36 | - |
|  |  |  | 0 | 0 | 4 | 0 | 0 | 4 | 15 |  |
| aldehyde dehydrogenase 7A1, NP_001173.2 | |  |  |  |  |  |  |  |  |  |
|  | C |  | 0.95 | 0.94 | - | 0.94 | 0.84 | - | 0.98 |  |
|  | DLPLAQGIK | 2 | 2.22 | 2.19 | - | - | 2.12 | - | 2.69 | - |
|  | EENEGVYNGSWGGR | 2 | 2.31 | - | - | - | - | - | 4.87 | - |
|  | EGGTVVYGGK | 2 | 2.73 | - | - | - | 2.52 | - | 2.74 | - |
|  | FKNEEEVFAWNNEVK | 2 | - | 4.19 | - | - | - | - | 6.32 | - |
|  | GAPTTSLISVAVTK | 2 | 5.23 | 4.97 | - | 4.73 | - | - | 4.70 | - |
|  | GEVITTYCPANNEPIAR | 2 | 5.15 | 4.86 | - | 4.80 | - | - | 5.65 | - |
|  | GSDCGIVNVNIPTSGAEIGGAFGGEK | 2 | 5.29 | 5.09 | - | - | - | - | 6.27 | - |
|  | ILVEGVGEVQEYVDICDYAVGLSR | 3 | 3.97 | 2.69 | - | - | - | - | - | - |
|  | IQVLGSLVSLEMGK | 2 | - | - | - | - | - | - | 5.12 | - |
|  | IWADIPAPK | 2 | - | 2.60 | - | - | - | - | 3.01 | - |
|  | MIGGPILPSER | 2 | - | - | - | - | - | - | 3.58 | - |
|  | QASVADYEETVKK | 2 | - | - | - | - | - | - | 2.73 | - |
|  | QAVSMFLGAVEEAK | 2 | 2.92 | 2.25 | - | - | 2.45 | - | - | - |
|  | QAVSMFLGAVEEAKK | 2 | - | - | - | - | - | - | 2.56 | - |
|  | STCTINYSK | 2 | - | - | - | - | - | - | 2.70 | - |
|  | VGNPWDPNVLYGPLHTK | 3 | - | - | - | 3.80 | 4.21 | - | - | - |
|  | VLEDNKLPGAICSLTCGGADIGTAMAK | 3 | - | - | - | - | - | - | 3.76 | - |
|  | VNLLSFTGSTQVGK | 2 | - | - | - | - | - | - | 4.66 | - |
|  |  |  | 8 | 8 | 0 | 3 | 4 | 0 | 15 |  |
| aldehyde dehydrogenase 8A1, NP_739577.1, NP_072090.1 | |  |  |  |  |  |  |  |  |  |
|  | C |  | - | - | - | - | - | - | 0.98 |  |
|  | AGVPPGVVNIVFGTGPR | 2 | - | - | - | - | - | - | 2.11 | - |
|  | ALAEGAQIWCGEGVDK | 2 | - | - | - | - | - | - | 5.33 | - |
|  | DSYDFFTEIK | 2 | - | - | - | - | - | - | 3.09 | - |
|  | FLPCSSYIDSYDPSTGEVYCR | 2 | - | - | - | - | - | - | 6.04 | - |
|  | ITQLSAPHCK | 2 | - | - | - | - | - | - | 2.99 | - |
|  | KLSLELGGK | 2 | - | - | - | - | - | - | 2.09 | - |
|  | NPAIIFEDANLDECIPATVR | 2 | - | - | - | - | - | - | 6.04 | - |
|  | PSELTSVTAWMLCK | 2 | - | - | - | - | - | - | 4.44 | - |
|  | VGIPSDPLVSIGALISK | 2 | - | - | - | - | - | - | 3.91 | - |
|  | VLNQVADLLEQSLEEFAQAESK | 2 | - | - | - | - | - | - | 7.15 | - |
|  | VPNSGKDEIEAAVK | 2 | - | - | - | - | - | - | 4.62 | - |
|  |  |  | 0 | 0 | 0 | 0 | 0 | 0 | 11 |  |
| aldehyde dehydrogenase 9A1, NP_000687.3 | |  |  |  |  |  |  |  |  |  |
|  | C |  | 0.93 | 0.93 | 0.93 | 0.94 | 0.94 | 0.94 | 0.96 |  |
|  | ANDTTFGLAAGVFTR | 2 | 2.40 | 2.65 | - | - | - | 3.57 | 4.55 | - |
|  | EILDKFTEEVVK | 2 | - | - | - | - | - | 4.19 | 4.55 | - |
|  | EVNLAVQNAK | 2 | 3.30 | 3.15 | - | 3.36 | - | 2.17 | 3.81 | - |
|  | GIKPVTLELGGK | 2 | 3.60 | 3.02 | 3.85 | 4.00 | - | 3.59 | - | - |
|  | IGDPLLEDTR | 2 | 2.93 | 3.19 | 3.76 | 3.74 | 3.45 | 3.88 | 4.04 | - |
|  | VEPADASGTEK | 2 | 2.28 | 2.44 | - | - | 2.38 | 2.20 | 2.29 | - |
|  | VIATFTCSGEK | 2 | - | - | - | - | - | 3.17 | 3.79 | - |
|  | VLCGGDIYVPEDPK | 2 | 4.56 | 4.39 | - | 4.20 | - | - | 4.73 | - |
|  | VTIEYYSQLK | 2 | 2.17 | - | - | - | - | - | 3.76 | - |
|  | VTLELGGK | 2 | 2.02 | - | - | - | - | - | - | NP_000686.2, NP_001026786.1, NP_001128639.1, NP_001128640.1, NP_000682.3, NP_001026976.1, NP_000683.3, NP_000681.2, NP_000373.1, NP_000680.2 |
|  | VVAELQAGTCFINNYNVSPVELPFGGYK | 3 | - | 2.70 | - | - | - | - | - | - |
|  |  |  | 8 | 7 | 2 | 4 | 2 | 7 | 8 |  |
| aldehyde dehydrogenase X, NP_000683.3 | |  |  |  |  |  |  |  |  |  |
|  | C |  | - | - | - | - | - | - | 0.97 |  |
|  | EAGFPPGVVNIITGYGPTAGAAIAQHVDVDK | 3 | - | - | - | - | - | - | 3.03 | - |
|  | EEIFGPVQPLFK | 2 | - | - | - | - | - | - | 3.91 | - |
|  | KIEEVVER | 2 | - | - | - | - | - | - | 2.87 | - |
|  | RVTLELGGK | 2 | - | - | - | - | - | - | 3.36 | NP_000681.2, NP_000680.2 |
|  | TFVEESIYNEFLER | 2 | - | - | - | - | - | - | 5.52 | - |
|  | VAEQTPLSALYLASLIK | 2 | - | - | - | - | - | - | 5.85 | - |
|  | VGNPFELDTQQGPQVDKEQFER | 3 | - | - | - | - | - | - | 3.69 | - |
|  | VTLELGGK | 2 | - | - | - | - | - | - | 2.23 | NP_000686.2, NP_001026786.1, NP_001128639.1, NP_001128640.1, NP_000687.3, NP_000682.3, NP_001026976.1, NP_000681.2, NP_000373.1, NP_000680.2 |
|  | VYLASLETLDNGKPFQESYALDLDEVIK | 3 | - | - | - | - | - | - | 2.93 | - |
|  |  |  | 0 | 0 | 0 | 0 | 0 | 0 | 9 |  |
| aldehyde dehydrogenase, dimeric NADP-preferring, NP_000682.3, NP_001128639.1, NP_001128640.1 | | | | |  |  |  |  |  |  |
|  | C |  | 0.95 | 0.92 | - | - | 0.95 | - | - |  |
|  | DLYPVINGGVPETTELLK | 2 | 2.72 | 3.23 | - | - | 4.33 | - | - | - |
|  | FDHILYTGSTGVGK | 3 | 3.53 | 2.91 | - | - | 3.68 | - | - | - |
|  | HLTPVTLELGGK | 2 | - | - | - | - | 2.77 | - | - | NP_000686.2, NP_001026786.1, NP_001026976.1, NP_000373.1 |
|  | IQQLEALQR | 2 | 3.55 | 3.46 | - | - | 3.63 | - | - | - |
|  | LIQEQEQELVGALAADLHK | 3 | 5.22 | 5.08 | - | - | 4.86 | - | - | - |
|  | LPEWAADEPVEK | 2 | - | - | - | - | 3.98 | - | - | - |
|  | NCDLDVACR | 2 | - | - | - | - | 3.39 | - | - | - |
|  | SFETFSHR | 2 | - | - | - | - | 2.09 | - | - | - |
|  | SLEEAIQFINQR | 2 | 3.61 | - | - | - | 3.79 | - | - | - |
|  | TRPLQFR | 2 | 2.01 | - | - | - | - | - | - | - |
|  | VAYGGTGDAATR | 2 | 4.06 | 3.76 | - | - | 3.55 | - | - | - |
|  | VTLELGGK | 2 | 2.02 | - | - | - | - | - | - | NP_000686.2, NP_001026786.1, NP_000687.3, NP_001026976.1, NP_000683.3, NP_000681.2, NP_000373.1, NP_000680.2 |
|  |  |  | 8 | 5 | 0 | 0 | 10 | 0 | 0 |  |
| aldehyde oxidase, NP_001150.3 | |  |  |  |  |  |  |  |  |  |
|  | C |  | - | - | - | - | - | 0.95 | 0.99 |  |
|  | ASELLFYVNGR | 2 | - | - | - | - | - | - | 2.52 | - |
|  | DACQTLLK | 2 | - | - | - | - | - | - | 2.09 | - |
|  | DEPGSYVPWNVPI | 2 | - | - | - | - | - | - | 2.32 | - |
|  | DILADVVQK | 2 | - | - | - | - | - | - | 3.24 | - |
|  | DILADVVQKLPEEK | 2 | - | - | - | - | - | - | 5.34 | - |
|  | EIDQTPYK | 1 | - | - | - | - | - | - | 1.97 | - |
|  | EIDQTPYKQEINAK | 2 | - | - | - | - | - | - | 4.26 | - |
|  | ELCISYGGVGPATICAK | 2 | - | - | - | - | - | - | 5.57 | - |
|  | ELLEFK | 1 | - | - | - | - | - | - | 2.11 | - |
|  | ENGVCCLDQGINGLPEFEEGSK | 2 | - | - | - | - | - | - | 4.79 | - |
|  | FLATDKVFCVGQLVCAVLADSEVQAK | 3 | - | - | - | - | - | 2.67 | - | - |
|  | FPVGLGSR | 2 | - | - | - | - | - | - | 2.58 | - |
|  | GEDMLITGGR | 2 | - | - | - | - | - | - | 3.26 | - |
|  | GEGQPFEYFVYGAACSEVEIDCLTGDHK | 3 | - | - | - | - | - | 3.97 | - | - |
|  | GFGFPQAALITESCITEVAAK | 2 | - | - | - | - | - | 5.92 | 6.40 | - |
|  | GLHGPLTLNSPLTPEK | 2 | - | - | - | - | - | - | 4.81 | - |
|  | GTSTETVPNANISGGSVVADLNGLAVK | 2 | - | - | - | - | - | - | 5.08 | - |
|  | GVFHPVIISPDR | 2 | - | - | - | - | - | - | 3.36 | - |
|  | HLGTLAGSQIR | 2 | - | - | - | - | - | - | 3.68 | - |
|  | HPDSDLNPILAVGNCTLNLLSK | 2 | - | - | - | - | - | 4.00 | 5.24 | - |
|  | IEELSVVNHAYNGLTLGAGLSLAQVK | 3 | - | - | - | - | - | 2.77 | 4.17 | - |
|  | IPAICDMPTELHIALLPPSQNSNTLYSSK | 3 | - | - | - | - | - | - | 6.36 | - |
|  | KLEYGNVDEAFK | 2 | - | - | - | - | - | - | 4.71 | - |
|  | LEYGNVDEAFK | 2 | - | - | - | - | - | - | 4.06 | - |
|  | LILNEVSLLGSAPGGK | 2 | - | - | - | - | - | - | 5.45 | - |
|  | MACEDKFTK | 2 | - | - | - | - | - | - | 3.19 | - |
|  | MPMSNVHLR | 2 | - | - | - | - | - | - | 2.04 | - |
|  | NHPEPTLDQLTDALGGNLCR | 2 | - | - | - | - | - | - | 4.96 | - |
|  | NLIQCWR | 2 | - | - | - | - | - | - | 2.68 | - |
|  | NMASLGGHIISR | 2 | - | - | - | - | - | - | 3.74 | - |
|  | NVDPETMLLPYLR | 2 | - | - | - | - | - | - | 3.38 | - |
|  | QENALAIVNSGMR | 2 | - | - | - | - | - | - | 4.50 | - |
|  | QIPLNEQFLSK | 2 | - | - | - | - | - | - | 2.36 | - |
|  | RLEPIISK | 2 | - | - | - | - | - | - | 2.80 | - |
|  | RVGGAFGGK | 2 | - | - | - | - | - | - | 2.43 | - |
|  | TGIIAAVTAFAANK | 2 | - | - | - | - | - | - | 5.20 | - |
|  | TGIIAAVTAFAANKHGRAVR | 3 | - | - | - | - | - | - | 2.65 | - |
|  | TNLPSNTAFR | 2 | - | - | - | - | - | - | 2.58 | NP_000370.2 |
|  | VFCVGQLVCAVLADSEVQAK | 2 | - | - | - | - | - | 4.13 | 7.76 | - |
|  | VFFGEGDGIIR | 2 | - | - | - | - | - | - | 3.79 | - |
|  | YESALEDLHSK | 2 | - | - | - | - | - | - | 3.00 | - |
|  | YIQDIVASTLK | 2 | - | - | - | - | - | - | 4.09 | - |
|  | YQNIGPK | 1 | - | - | - | - | - | - | 2.21 | - |
|  |  |  | 0 | 0 | 0 | 0 | 0 | 6 | 41 |  |
| aldo-keto reductase 1A1, NP_697021.1, NP_006057.1 | |  |  |  |  |  |  |  |  |  |
|  | C |  | 0.97 | - | - | 0.97 | 0.98 | 0.98 | 0.98 |  |
|  | ALEALVAK | 2 | - | - | - | - | - | 2.68 | 2.83 | - |
|  | AWRDPDEPVLLEEPVVLALAEK | 3 | 6.14 | - | - | - | 5.94 | 4.01 | 6.96 | - |
|  | DAGHPLYPFNDPY | 2 | - | - | - | - | - | - | 3.13 | - |
|  | DPDEPVLLEEPVVLALAEK | 3 | 5.71 | - | - | 5.50 | 6.12 | 6.14 | 6.75 | - |
|  | EELFVTSK | 1 | - | - | - | - | - | 2.08 | 2.50 | - |
|  | GDNPFPK | 1 | - | - | - | - | - | - | 1.93 | - |
|  | GLEVTAYSPLGSSDR | 2 | - | - | - | - | 4.50 | 4.61 | 5.24 | - |
|  | GLVQALGLSNFNSR | 3 | - | - | - | 3.33 | 2.57 | 3.71 | 5.40 | - |
|  | HHPEDVEPALR | 3 | - | - | - | 3.00 | - | - | 3.75 | - |
|  | HHPEDVEPALRK | 3 | - | - | - | - | - | - | 3.93 | - |
|  | HIDCAAIYGNEPEIGEALK | 3 | - | - | - | - | - | - | 4.55 | - |
|  | HIDCAAIYGNEPEIGEALKEDVGPGK | 3 | - | - | - | - | - | - | 7.87 | - |
|  | ILQNIK | 1 | - | - | - | - | - | - | 2.17 | - |
|  | KVICIPK | 2 | - | - | - | - | - | 2.29 | - | - |
|  | MPLIGLGTWK | 2 | - | - | - | - | - | - | 3.23 | - |
|  | NADGTICYDSTHYK | 2 | - | - | - | - | - | 2.94 | 4.90 | - |
|  | QIDDILSVASVR | 2 | - | - | - | - | - | 2.57 | 2.81 | - |
|  | SPAQILLR | 2 | - | - | - | - | 3.25 | - | 3.46 | - |
|  | VFDFTFSPEEMK | 2 | - | - | - | - | - | 2.14 | 3.87 | - |
|  | YALSVGYR | 2 | - | - | - | - | - | - | 3.16 | - |
|  | YIVPMLTVDGK | 2 | - | - | - | - | - | - | 3.36 | - |
|  |  |  | 2 | 0 | 0 | 3 | 5 | 10 | 20 |  |
| aldo-keto reductase 1B, NP_001074007.2, NP_064695.3 | |  |  |  |  |  |  |  |  |  |
|  | C |  | 0.97 | 0.97 | 0.97 | 0.96 | 0.96 | - | - |  |
|  | ACNVLQSSHLEDYPFNAEY | 2 | 2.50 | 2.51 | 3.40 | 2.52 | - | - | - | - |
|  | ALGVSNFSHFQIEK | 2 | 4.40 | 4.53 | 3.76 | - | - | - | - | - |
|  | ATFLDAWEAMEELVDEGLVK | 3 | 6.40 | 6.29 | 6.62 | 6.45 | 5.88 | - | - | - |
|  | GITVTAYSPLGSPDRPWAKPEDPSLLEDPK | 3 | 5.70 | 4.70 | 4.00 | 4.06 | - | - | - | - |
|  | HIDCAYVYQNEHEVGEAIQEK | 3 | 6.22 | 6.77 | 5.65 | 5.50 | 3.77 | - | - | - |
|  | IVENIQVFDFK | 2 | 4.57 | 4.64 | 4.51 | 4.51 | 4.39 | - | - | - |
|  | LLNKPGLK | 2 | 2.28 | 2.38 | 2.02 | - | - | - | - | - |
|  | LSDEEMATILSFNR | 2 | 4.09 | 4.61 | 4.12 | 4.18 | - | - | - | - |
|  | LSYLDVYLIHWPQGFK | 2 | 5.25 | 5.13 | 5.09 | 4.63 | - | - | - | - |
|  | REDLFIVSK | 2 | 3.09 | 3.09 | 2.81 | 2.93 | 2.42 | - | - | - |
|  | SGDDLFPK | 2 | 3.01 | 2.95 | 2.30 | - | - | - | - | - |
|  | TAAQVLIR | 2 | 3.07 | 3.16 | 2.81 | 3.17 | - | - | - | - |
|  | TTAQVLIR | 2 | 2.42 | 2.08 | - | 2.72 | 2.75 | - | - | - |
|  | VAIDAGYR | 2 | 2.71 | 2.89 | - | 2.66 | - | - | - | - |
|  | YKPVTNQVECHPYLTQEK | 3 | 5.39 | 2.81 | - | 2.75 | 2.70 | - | - | - |
|  |  |  | 15 | 15 | 12 | 12 | 6 | 0 | 0 |  |
| aldo-keto reductase 1C, NP_001809.2, NP_995317.1, NP_001128713.1, NP_001345.1, NP_001344.2, NP_003730.4 | | | | | |  |  |  |  |  |
|  | C |  | 0.97 | 0.98 | 0.96 | 0.97 | 0.97 | 0.98 | 0.98 |  |
|  | DIVLVAYSALGSHR | 2 | 4.99 | 4.45 | 4.38 | - | 2.82 | - | - | - |
|  | DIVLVAYSALGSQR | 2 | 2.37 | 2.64 | - | - | - | - | - | - |
|  | EDIFYTSK | 1 | - | - | - | - | - | - | 2.22 | - |
|  | EEPWVDPNSPVLLEDPVLCALAK | 3 | 6.32 | 6.75 | 5.23 | 4.91 | 5.47 | 7.20 | 7.37 | - |
|  | GVVVLAK | 1 | 1.83 | 1.71 | - | - | - | - | 1.88 | - |
|  | HIDSAHLYNNEEQVGLAIR | 3 | 5.73 | 6.09 | - | - | - | 5.37 | 6.73 | - |
|  | HIDSAYLYNNEEQVGLAIR | 2 | - | - | - | - | - | - | 6.83 | - |
|  | IADGSVKR | 2 | - | - | - | - | - | - | 2.18 | - |
|  | ILFDTVDLCATWEAMEK | 2 | 2.21 | 3.24 | - | - | - | - | 5.28 | - |
|  | ILFDTVDLCATWEAVEK | 2 | 4.90 | 5.86 | 3.59 | 2.71 | - | - | 5.97 | - |
|  | IRENIQVFEFQLTSEDMK | 2 | - | - | - | - | - | - | 2.18 | - |
|  | KLLDFCK | 2 | 2.99 | 2.84 | 2.95 | - | 2.49 | 2.93 | 3.02 | - |
|  | LAIEAGFHHIDSAHVYNNEEQVGLAIR | 3 | 3.49 | 3.21 | - | - | - | - | - | - |
|  | LAIEAGFR | 2 | 2.75 | 2.84 | 2.71 | 2.54 | - | 2.61 | 2.93 | - |
|  | LLDFCK | 1 | - | - | - | - | - | - | 1.93 | - |
|  | LLEMILNKPGLK | 2 | 3.16 | 2.66 | 2.67 | - | - | - | 4.11 | - |
|  | LNDGHFMPVLGFGTYAPAEVPK | 2 | 5.02 | 4.82 | 4.62 | 4.17 | 4.15 | - | 5.87 | - |
|  | LNDGHFMPVLGFGTYAPPEVPR | 3 | - | 3.09 | - | - | - | - | 4.43 | - |
|  | LWCNSHRPELVRPALER | 3 | - | 2.96 | - | - | - | - | - | - |
|  | LWCTFFQPQMVQPALESSLK | 3 | - | - | - | - | - | - | 6.46 | - |
|  | LWSNSHRPELVR | 3 | - | 2.61 | - | - | - | - | - | - |
|  | LWSNSHRPELVRPALER | 3 | 3.63 | 3.16 | 2.74 | - | 3.13 | - | - | - |
|  | LWVDPNSPVLLEDPVLCALAK | 3 | - | - | - | - | - | - | 6.66 | - |
|  | NLHYFNSDSFASHPNYPYSDEY | 3 | - | 2.65 | 2.80 | - | - | - | - | - |
|  | NLQLDYVDLYLIHFPVSVKPGEEVIPK | 3 | 7.52 | 7.58 | 7.38 | 7.04 | 7.37 | - | 7.69 | - |
|  | QLEMILNKPGLK | 2 | 3.23 | 3.00 | 2.86 | 3.04 | - | - | 3.35 | - |
|  | QNVQVFEFQLTAEDMK | 2 | - | - | - | - | - | - | 2.60 | - |
|  | REDIFYTSK | 2 | 3.40 | 3.30 | 3.15 | 2.88 | 3.06 | 2.91 | 3.38 | - |
|  | RTPALIALR | 2 | 3.29 | 3.13 | 3.04 | - | 2.86 | 3.23 | 3.50 | - |
|  | SIGVSNFNCR | 2 | - | - | - | - | - | - | 3.67 | - |
|  | SIGVSNFNHR | 2 | 3.38 | 3.31 | 2.98 | - | 3.02 | - | 3.63 | - |
|  | SIGVSNFNR | 2 | 2.93 | 3.05 | 2.79 | - | - | - | 2.92 | - |
|  | SKDIVLVAHSALGTQR | 3 | - | - | - | - | - | - | 5.92 | - |
|  | SKDIVLVAYSALGSHR | 3 | 5.67 | 6.04 | 5.95 | - | 5.18 | - | 5.91 | - |
|  | SKDIVLVAYSALGSQR | 3 | 3.72 | 4.38 | - | - | - | - | 4.65 | - |
|  | TPALIALR | 2 | 3.37 | 3.21 | 3.18 | 3.01 | 3.03 | 3.16 | 3.28 | - |
|  | VELNDGHFMPVLGFGTYAPPEVPR | 2 | - | - | - | - | - | - | 5.58 | - |
|  | WVDPNSPVLLEDPVLCALAK | 3 | 4.85 | 6.42 | 4.49 | - | - | 5.21 | 6.16 | - |
|  | YKPVCNQVECHPYFNQR | 2 | 3.64 | 4.38 | 3.48 | 3.58 | 3.27 | 3.39 | 5.10 | - |
|  | YKPVCNQVECHPYFNR | 3 | - | 3.02 | 3.21 | - | - | - | 3.75 | - |
|  | YKPVCNQVECHPYLNQSK | 2 | - | - | - | - | - | - | 5.74 | - |
|  | YLTLDIFAGPPNYPFSDEY | 2 | 5.42 | 5.49 | 4.59 | - | - | 5.10 | 5.21 | - |
|  | YVVMDFLMDHPDYPFSDEY | 2 | - | - | - | - | - | - | 4.89 | - |
|  |  |  | 25 | 30 | 21 | 9 | 12 | 10 | 36 |  |
| amine oxidase [flavin-containing] A, NP_000231.1 | |  |  |  |  |  |  |  |  |  |
|  | M |  | 0 | 0 | 0 | 0 | 0 | 0.936 | 0.983 |  |
|  | DIWVQEPESK | 2 | - | - | - | - | - | - | 3.58 | - |
|  | DVPAVEITHTFWER | 2 | - | - | - | - | - | - | 5.07 | - |
|  | ELGIETYK | 1 | - | - | - | - | - | - | 2.30 | - |
|  | FVGGSGQVSER | 2 | - | - | - | - | - | 3.32 | 3.79 | NP_000889.3 |
|  | ICELYAK | 2 | - | - | - | - | - | - | 2.30 | - |
|  | IFFAGTETATK | 2 | - | - | - | - | - | - | 3.81 | - |
|  | IFSVTNGGQER | 2 | - | - | - | - | - | - | 3.80 | - |
|  | IHFRPELPAER | 2 | - | - | - | - | - | - | 3.24 | - |
|  | IMDLLGDQVK | 2 | - | - | - | - | - | - | 3.68 | - |
|  | KFVGGSGQVSER | 3 | - | - | - | - | - | 3.98 | 4.69 | NP_000889.3 |
|  | KICELYAK | 2 | - | - | - | - | - | - | 3.11 | - |
|  | LLTEYGVSVLVLEAR | 2 | - | - | - | - | - | - | 5.00 | - |
|  | LPMGAVIK | 2 | - | - | - | - | - | - | 2.53 | - |
|  | NEHVDYVDVGGAYVGPTQNR | 2 | - | - | - | - | - | 3.89 | 6.78 | - |
|  | NLPSVSGLLK | 1 | - | - | - | - | - | - | 1.94 | - |
|  | VLGSQEALHPVHYEEK | 2 | - | - | - | - | - | - | 4.98 | - |
|  | VTEKDIWVQEPESK | 3 | - | - | - | - | - | - | 5.03 | - |
|  | WSGYMEGAVEAGER | 2 | - | - | - | - | - | - | 3.05 | NP_000889.3 |
|  | YVINAIPPTLTAK | 2 | - | - | - | - | - | 3.41 | 3.84 | - |
|  |  |  | 0 | 0 | 0 | 0 | 0 | 4 | 19 |  |
| amine oxidase [flavin-containing] B, NP_000889.3 | |  |  |  |  |  |  |  |  |  |
|  | M |  | 0 | 0 | 0 | 0 | 0 | 0.963 | 0.969 |  |
|  | APLAEEWDNMTMK | 2 | - | - | - | - | - | - | 4.82 | - |
|  | CIVYYK | 2 | - | - | - | - | - | - | 2.05 | - |
|  | EIPSDAPWK | 2 | - | - | - | - | - | - | 2.06 | - |
|  | ELLDKLCWTESAK | 2 | - | - | - | - | - | - | 4.05 | - |
|  | ENVLVETLNHEMYEAK | 2 | - | - | - | - | - | - | 5.61 | - |
|  | FVGGSGQVSER | 2 | - | - | - | - | - | 3.32 | 3.79 | NP_000231.1 |
|  | HLPSVPGLLR | 2 | - | - | - | - | - | - | 2.11 | - |
|  | IHFNPPLPMMR | 3 | - | - | - | - | - | - | 3.12 | - |
|  | IISTTNGGQER | 2 | - | - | - | - | - | 2.55 | 3.76 | - |
|  | IMDLLGDR | 2 | - | - | - | - | - | 2.87 | 2.92 | - |
|  | IPEDEIWQSEPESVDVPAQPITTTFLER | 3 | - | - | - | - | - | 5.67 | 6.10 | - |
|  | IYFAGTETATHWSGYMEGAVEAGER | 3 | - | - | - | - | - | - | 5.81 | - |
|  | KFVGGSGQVSER | 3 | - | - | - | - | - | 4.20 | 4.69 | NP_000231.1 |
|  | LCWTESAK | 2 | - | - | - | - | - | - | 2.65 | - |
|  | LERPVIYIDQTR | 3 | - | - | - | - | - | 3.49 | 4.65 | - |
|  | LLHDSGLNVVVLEAR | 2 | - | - | - | - | - | 3.28 | 5.03 | - |
|  | PVIYIDQTR | 2 | - | - | - | - | - | - | 2.37 | - |
|  | VKLERPVIYIDQTR | 3 | - | - | - | - | - | - | 3.72 | - |
|  | VLGSLEALEPVHYEEK | 2 | - | - | - | - | - | 2.98 | 4.88 | - |
|  | VPLGSVIK | 2 | - | - | - | - | - | 2.31 | 2.44 | - |
|  | WSGYMEGAVEAGER | 2 | - | - | - | - | - | - | 3.05 | NP_000231.1 |
|  | YVDLGGSYVGPTQNR | 2 | - | - | - | - | - | 5.29 | 5.66 | - |
|  | YVISAIPPTLGMK | 2 | - | - | - | - | - | - | 3.22 | - |
|  |  |  | 0 | 0 | 0 | 0 | 0 | 10 | 23 |  |
| bile salt sulfotransferase, NP_003158.2 | |  |  |  |  |  |  |  |  |  |
|  | C |  | - | - | - | - | - | - | 0.98 |  |
|  | DEDVIILTYPK | 2 | - | - | - | - | - | - | 4.06 | - |
|  | DEFVIRDEDVIILTYPK | 2 | - | - | - | - | - | - | 2.88 | - |
|  | ICQFLGK | 2 | - | - | - | - | - | - | 2.42 | - |
|  | KGVSGDWK | 2 | - | - | - | - | - | - | 2.19 | - |
|  | LFSSHLPIQLFPK | 3 | - | - | - | - | - | - | 3.98 | - |
|  | MSNYSLLSVDYVVDK | 2 | - | - | - | - | - | - | 5.24 | - |
|  | NFLLLSYEELK | 2 | - | - | - | - | - | - | 3.78 | - |
|  | NFLLLSYEELKQDTGR | 3 | - | - | - | - | - | - | 2.53 | - |
|  | NHFTVAQAEDFDK | 3 | - | - | - | - | - | - | 4.91 | - |
|  | NHFTVAQAEDFDKLFQEK | 3 | - | - | - | - | - | - | 4.34 | - |
|  | NSSFQSMK | 2 | - | - | - | - | - | - | 2.67 | - |
|  | SPWVESEIGYTALSETESPR | 2 | - | - | - | - | - | - | 7.53 | - |
|  | TLEPEELNLILK | 2 | - | - | - | - | - | - | 3.92 | - |
|  | VRDEFVIRDEDVIILTYPK | 0 | - | - | - | - | - | - | - | - |
|  | WIQSVPIWER | 2 | - | - | - | - | - | - | 3.34 | - |
|  |  |  | 0 | 0 | 0 | 0 | 0 | 0 | 14 |  |
| carbonyl reductase 4, NP_116172.2 | |  |  |  |  |  |  |  |  |  |
|  | C |  | - | - | - | - | - | - | 0.96 |  |
|  | AAAGDLGGDHLAFSCDVAK | 3 | - | - | - | - | - | - | 3.49 | - |
|  | VNFLVNAAGINR | 2 | - | - | - | - | - | - | 3.99 | - |
|  | VNVVAPGFVHTDMTK | 2 | - | - | - | - | - | - | 2.30 | - |
|  |  |  | 0 | 0 | 0 | 0 | 0 | 0 | 3 |  |
| carbonyl reductase [NADPH] 1, NP_001748.1 | |  |  |  |  |  |  |  |  |  |
|  | C |  | 0.97 | 0.97 | 0.97 | 0.97 | 0.97 | 0.97 | 0.98 |  |
|  | DVCTELLPLIKPQGR | 2 | 3.94 | 3.87 | 3.87 | 3.61 | - | 4.11 | 4.53 | - |
|  | EGWPSSAYGVTK | 2 | 2.76 | 2.80 | 2.83 | 2.73 | 2.63 | 2.36 | 2.96 | - |
|  | EYGGLDVLVNNAGIAFK | 2 | 5.34 | 5.28 | 3.72 | 5.56 | 5.21 | 4.12 | 5.66 | - |
|  | FHQLDIDDLQSIR | 2 | 4.98 | 4.85 | 4.69 | 5.05 | 4.50 | - | 5.15 | NP_001227.1 |
|  | FRSETITEEELVGLMNK | 2 | - | - | - | - | - | 2.58 | 5.10 | - |
|  | FVEDTKK | 2 | - | - | - | - | - | - | 2.39 | - |
|  | GIGLAIVR | 2 | 2.98 | 2.82 | - | 2.67 | 2.59 | - | 2.81 | - |
|  | GQAAVQQLQAEGLSPR | 2 | 5.36 | 5.27 | 5.26 | 5.20 | 5.25 | 5.67 | 5.83 | NP_001227.1 |
|  | IGVTVLSR | 2 | 3.05 | 2.98 | 2.94 | - | - | - | - | - |
|  | ILLNACCPGWVR | 2 | 4.38 | 4.13 | 4.29 | 3.97 | - | 4.27 | 4.45 | - |
|  | KEYGGLDVLVNNAGIAFK | 3 | 4.26 | 4.54 | 4.24 | 4.83 | - | - | 4.18 | - |
|  | LFSGDVVLTAR | 2 | 3.85 | 3.99 | 3.77 | 3.87 | 3.88 | 3.89 | 4.22 | - |
|  | SCSPELQQK | 2 | 2.17 | - | - | 2.16 | - | 2.08 | 2.41 | - |
|  | SETITEEELVGLMNK | 2 | 4.94 | 4.54 | 4.85 | 4.64 | 4.40 | 3.56 | 5.16 | - |
|  | SETITEEELVGLMNKFVEDTKK | 3 | 2.93 | - | 2.89 | 2.71 | 2.89 | - | 2.86 | - |
|  | SPEEGAETPVYLALLPPDAEGPHGQFVSEK | 3 | 6.71 | 6.85 | 7.04 | 6.98 | 6.05 | 7.00 | 7.66 | - |
|  | VVNVSSIMSVR | 2 | - | - | 3.20 | 3.04 | - | 4.27 | 4.72 | - |
|  |  |  | 14 | 12 | 13 | 14 | 9 | 11 | 16 |  |
| carbonyl reductase [NADPH] 3, NP_001227.1 | |  |  |  |  |  |  |  |  |  |
|  | C |  | 0.97 | 0.97 | 0.97 | 0.97 | 0.97 | 0.97 | 0.97 |  |
|  | AFENCSEDLQER | 2 | - | - | - | - | - | 4.00 | - | - |
|  | EYGGLNVLVNNAAVAFK | 2 | 2.23 | 2.64 | 3.24 | 2.23 | 2.02 | 5.08 | - | - |
|  | FHQLDIDDLQSIR | 2 | 4.98 | 4.85 | 4.69 | 5.05 | 4.50 | - | 5.15 | NP_001748.1 |
|  | FVEDTKNEVHER | 3 | - | - | - | - | - | 3.14 | - | - |
|  | GQAAVQQLQAEGLSPR | 2 | 5.36 | 5.27 | 5.26 | 5.20 | 5.25 | 5.10 | 5.83 | NP_001748.1 |
|  | ILVNACCPGPVK | 2 | - | - | - | - | - | 3.85 | - | - |
|  | TVEEGAETPVYLALLPPDATEPQGQLVHDK | 3 | - | - | - | - | 5.56 | 6.17 | - | - |
|  | VALVTGANR | 2 | - | - | - | - | - | 2.00 | - | - |
|  |  |  | 3 | 3 | 3 | 3 | 4 | 7 | 2 |  |
| carboxylesterase 2, NP_003860.2, NP_932327.1 | |  |  |  |  |  |  |  |  |  |
|  | M |  | - | - | - | - | - | - | 0.97 |  |
|  | ADHGDELPFVFR | 2 | - | - | - | - | - | - | 3.43 | - |
|  | APVYFYEFQHQPSWLK | 3 | - | - | - | - | - | - | 3.85 | - |
|  | EMDREASQAALQK | 2 | - | - | - | - | - | - | 3.24 | - |
|  | FAPPEPPESWSGVR | 3 | - | - | - | - | - | - | 4.12 | - |
|  | FTEEEEQLSR | 2 | - | - | - | - | - | - | 3.55 | - |
|  | FTEEEEQLSRK | 3 | - | - | - | - | - | - | 3.16 | - |
|  | HATGNWGYLDQVAALR | 2 | - | - | - | - | - | - | 5.19 | - |
|  | HPQELLASADFQPVPSIVGVNNNEFGWLIPK | 3 | - | - | - | - | - | - | 7.60 | - |
|  | IQELEEPEER | 2 | - | - | - | - | - | - | 3.76 | - |
|  | LGVLGFFSTGDK | 2 | - | - | - | - | - | - | 4.15 | - |
|  | MIPGVVDGVFLPR | 2 | - | - | - | - | - | - | 3.85 | - |
|  | NIRPPHMK | 2 | - | - | - | - | - | - | 2.41 | - |
|  | SFFGGNYIK | 2 | - | - | - | - | - | - | 2.44 | - |
|  | SKEEILAINKPFK | 3 | - | - | - | - | - | - | 4.71 | - |
|  | TTHTGQVLGSLVHVK | 3 | - | - | - | - | - | - | 5.27 | - |
|  | WVQQNIAHFGGNPDR | 2 | - | - | - | - | - | - | 5.29 | - |
|  | YWANFAR | 2 | - | - | - | - | - | - | 2.75 | - |
|  |  |  | 0 | 0 | 0 | 0 | 0 | 0 | 17 |  |
| catalase, NP_001743.1 | |  |  |  |  |  |  |  |  |  |
|  | C |  | 0.97 | 0.97 | 0.97 | 0.97 | 0.92 | 0.97 | 0.98 |  |
|  | ADVLTTGAGNPVGDK | 2 | 4.93 | 4.99 | 4.70 | 4.76 | - | 4.83 | 5.02 | - |
|  | ADVLTTGAGNPVGDKLNVITVGPR | 3 | 5.93 | 5.72 | 4.63 | 4.96 | - | - | 6.40 | - |
|  | AFYVNVLNEEQR | 2 | 4.48 | 4.70 | 3.72 | 3.76 | - | 4.31 | 4.79 | - |
|  | DAQIFIQK | 2 | 3.25 | 3.24 | 3.09 | 3.17 | - | - | 3.33 | - |
|  | DLFNAIATGK | 2 | 3.11 | 2.93 | 2.94 | 2.77 | - | - | 2.29 | - |
|  | DPASDQMQHWK | 2 | - | - | - | - | - | - | 3.89 | - |
|  | DPILFPSFIHSQK | 2 | 3.63 | 3.70 | 3.89 | 3.33 | - | 2.22 | 3.84 | - |
|  | DYPLIPVGK | 2 | 2.31 | 2.39 | 2.20 | - | - | - | - | - |
|  | FNTANDDNVTQVR | 2 | 4.38 | 4.18 | 4.47 | 4.59 | 3.39 | 4.59 | 4.91 | - |
|  | FSTVAGESGSADTVR | 2 | 5.29 | 5.64 | 4.75 | - | - | - | 5.35 | - |
|  | FSTVAGESGSADTVRDPR | 3 | - | - | - | - | - | - | 3.06 | - |
|  | FYTEDGNWDLVGNNTPIFFIR | 2 | - | - | 5.67 | - | - | - | 6.28 | - |
|  | GAGAFGYFEVTHDITK | 2 | 4.82 | 2.33 | - | 2.40 | - | 2.43 | 5.18 | - |
|  | GPLLVQDVVFTDEMAHFDR | 3 | 2.70 | 2.86 | 3.25 | - | - | - | 3.36 | - |
|  | LCENIAGHLK | 2 | 3.28 | 3.10 | - | - | - | 2.30 | 3.66 | - |
|  | LFAYPDTHR | 2 | 2.86 | 2.72 | 2.66 | - | - | 2.78 | 3.34 | - |
|  | LGPNYLHIPVNCPYR | 2 | 4.44 | 4.16 | 4.39 | 4.29 | - | - | 4.21 | - |
|  | LNVITVGPR | 2 | 3.40 | 3.35 | 3.34 | 3.28 | 3.14 | 3.26 | 3.55 | - |
|  | LSQEDPDYGIR | 2 | 3.67 | 3.59 | 3.47 | 3.50 | - | 3.11 | 3.60 | - |
|  | LVNANGEAVYCK | 2 | 3.95 | 4.20 | 2.67 | 2.83 | - | 2.82 | 4.19 | - |
|  | NAIHTFVQSGSHLAAR | 3 | 4.38 | 4.54 | 3.75 | 3.78 | 3.65 | 2.99 | 5.62 | - |
|  | NFTEVHPDYGSHIQALLDK | 3 | 4.75 | 5.11 | 4.58 | 4.54 | - | - | 2.98 | - |
|  | NLSVEDAAR | 2 | 2.61 | 2.45 | 2.08 | 2.19 | - | 2.61 | 2.84 | - |
|  | NPVNYFAEVEQIAFDPSNMPPGIEASPDK | 3 | 6.26 | 6.64 | 5.83 | - | - | - | 7.53 | - |
|  | RFNTANDDNVTQVR | 2 | - | - | - | - | - | - | 6.01 | - |
|  | VFEHIGK | 2 | - | - | - | - | - | - | 2.32 | - |
|  | VWPHKDYPLIPVGK | 3 | 3.04 | 2.63 | - | - | - | - | - | - |
|  |  |  | 22 | 22 | 20 | 15 | 3 | 12 | 25 |  |
| catechol O-methyltransferase, NP_000745.1, NP_009294.1, NP_001128633.1, NP_001128634.1 | | | | |  |  |  |  |  |  |
|  | C |  | 0.96 | 0.97 | 0.96 | 0.97 | 0.97 | - | 0.98 |  |
|  | AIYKGPGSEAGP | 2 | - | - | - | - | - | - | 4.01 | - |
|  | EVVDGLEK | 1 | 1.66 | 2.09 | - | 1.95 | - | - | - | - |
|  | GSSCFECTHYQSFLEYR | 2 | 3.08 | 2.66 | - | 2.60 | 2.84 | - | 5.56 | - |
|  | GTVLLADNVICPGAPDFLAHVR | 3 | 5.52 | 5.78 | 5.87 | 5.00 | 5.40 | - | 5.60 | - |
|  | ILNHVLQHAEPGNAQSVLEAIDTYCEQK | 3 | 5.78 | 6.60 | - | - | - | - | 8.48 | - |
|  | IVDAVIQEHQPSVLLELGAYCGYSAVR | 3 | 5.41 | 4.91 | 4.08 | 5.35 | 5.27 | - | 5.83 | - |
|  | LITIEINPDCAAITQR | 2 | 5.05 | 5.51 | 2.24 | 4.81 | 4.96 | - | 5.34 | - |
|  | VTLVVGASQDIIPQLK | 2 | 3.20 | 2.88 | - | 2.55 | 2.24 | - | 3.72 | - |
|  | YDVDTLDMVFLDHWK | 3 | - | - | - | - | - | - | 3.84 | - |
|  | YLPDTLLLEECGLLR | 2 | 5.81 | 5.27 | 5.19 | 5.41 | 5.31 | - | 5.61 | - |
|  |  |  | 8 | 8 | 4 | 7 | 6 | 0 | 9 |  |
| cytochrome P450 1A2, NP_000752.2 | |  |  |  |  |  |  |  |  |  |
|  | M |  | - | - | - | - | - | - | 0.97 |  |
|  | ASGNLIPQEK | 2 | - | - | - | - | - | - | 3.42 | - |
|  | DITGALFK | 2 | - | - | - | - | - | - | 2.57 | - |
|  | DTTLNGFYIPK | 2 | - | - | - | - | - | - | 3.42 | - |
|  | ELDTVIGR | 1 | - | - | - | - | - | - | 2.36 | - |
|  | FLTADGTAINKPLSEK | 2 | - | - | - | - | - | - | 5.42 | - |
|  | FLWFLQK | 2 | - | - | - | - | - | - | 2.60 | - |
|  | GRPDLYTSTLITDGQSLTFSTDSGPVWAAR | 3 | - | - | - | - | - | - | 7.02 | - |
|  | HSSFLPFTIPHSTTR | 3 | - | - | - | - | - | - | 3.90 | - |
|  | IGSTPVLVLSR | 2 | - | - | - | - | - | - | 3.84 | - |
|  | LAQNALNTFSIASDPASSSSCYLEEHVSK | 3 | - | - | - | - | - | - | 6.86 | - |
|  | MMLFGMGK | 2 | - | - | - | - | - | - | 2.38 | - |
|  | NTHEFVETASSGNPLDFFPILR | 2 | - | - | - | - | - | - | 6.37 | - |
|  | TVQEHYQDFDK | 2 | - | - | - | - | - | - | 3.99 | - |
|  | TVQEHYQDFDKNSVR | 3 | - | - | - | - | - | - | 3.80 | - |
|  | VDLTPIYGLTMK | 2 | - | - | - | - | - | - | 4.33 | - |
|  | YGDVLQIR | 2 | - | - | - | - | - | - | 3.65 | - |
|  | YLPNPALQR | 2 | - | - | - | - | - | - | 2.35 | - |
|  |  |  | 0 | 0 | 0 | 0 | 0 | 0 | 17 |  |
| cytochrome P450 2A6/2A7/2A13, NP_000753.3, NP_000755.2, NP_000757.2, NP_085079.2 | | | | |  |  |  |  |  |  |
|  | M |  | - | - | - | - | - | - | 0.97 |  |
|  | DFIDSFLIR | 2 | - | - | - | - | - | - | 3.64 | - |
|  | DKEFLSLLR | 2 | - | - | - | - | - | - | 2.81 | - |
|  | DPSFFSNPQDFNPQHFLNEK | 2 | - | - | - | - | - | - | 5.06 | - |
|  | EALVDQAEEFSGR | 2 | - | - | - | - | - | - | 4.50 | - |
|  | FGDVIPMSLAR | 2 | - | - | - | - | - | - | 3.85 | - |
|  | FSIATLR | 2 | - | - | - | - | - | - | 2.33 | - |
|  | GEQATFDWVFK | 2 | - | - | - | - | - | - | 3.97 | - |
|  | GTEVFPMLGSVLR | 2 | - | - | - | - | - | - | 3.58 | - |
|  | GTEVYPMLGSVLR | 2 | - | - | - | - | - | - | 3.72 | - |
|  | GTGGANIDPTFFLSR | 2 | - | - | - | - | - | - | 4.39 | - |
|  | GYGVVFSNGER | 2 | - | - | - | - | - | - | 4.28 | - |
|  | HVGFATIPR | 2 | - | - | - | - | - | - | 2.89 | - |
|  | IQEEAGFLIDALR | 2 | - | - | - | - | - | - | 5.15 | - |
|  | KSDAFVPFSIGK | 2 | - | - | - | - | - | - | 4.20 | - |
|  | MPYMEAVIHEIQR | 3 | - | - | - | - | - | - | 3.83 | - |
|  | MQEEEKNPNTEFYLK | 2 | - | - | - | - | - | - | 5.19 | - |
|  | NCFGEGLAR | 2 | - | - | - | - | - | - | 3.01 | - |
|  | NPNTEFYLK | 2 | - | - | - | - | - | - | 2.99 | - |
|  | NYTMSFLPR | 2 | - | - | - | - | - | - | 3.52 | - |
|  | RFSIATLR | 2 | - | - | - | - | - | - | 2.66 | - |
|  | RGIEERIQEEAGFLIDALR | 3 | - | - | - | - | - | - | 2.86 | - |
|  | SDAFVPFSIGK | 2 | - | - | - | - | - | - | 4.46 | - |
|  | TLDPNSPR | 2 | - | - | - | - | - | - | 2.29 | - |
|  | TVSNVISSIVFGDR | 2 | - | - | - | - | - | - | 4.76 | - |
|  | VVVLCGHDAVR | 2 | - | - | - | - | - | - | 3.40 | - |
|  | YGFLLLMK | 2 | - | - | - | - | - | - | 3.43 | - |
|  |  |  | 0 | 0 | 0 | 0 | 0 | 0 | 26 |  |
| cytochrome P450 2B6, NP_000758.1 | |  |  |  |  |  |  |  |  |  |
|  | M |  | - | - | - | - | - | - | 0.98 |  |
|  | DLIDTYLLHMEK | 2 | - | - | - | - | - | - | 4.54 | - |
|  | EALVDKAEAFSGR | 3 | - | - | - | - | - | - | 3.43 | - |
|  | GYGVIFANGNR | 2 | - | - | - | - | - | - | 2.64 | - |
|  | HRETLDPSAPK | 3 | - | - | - | - | - | - | 3.22 | - |
|  | IAMVDPFFR | 2 | - | - | - | - | - | - | 2.88 | - |
|  | IQEEAQCLIEELR | 2 | - | - | - | - | - | - | 4.90 | - |
|  | IQEEAQCLIEELRK | 2 | - | - | - | - | - | - | 4.76 | - |
|  | KTEAFIPFSLGK | 3 | - | - | - | - | - | - | 2.69 | - |
|  | MPYTEAVIYEIQR | 2 | - | - | - | - | - | - | 5.59 | - |
|  | NLQEINAYIGHSVEK | 2 | - | - | - | - | - | - | 5.35 | - |
|  | PLPLLGNLLQMDR | 2 | - | - | - | - | - | - | 3.30 | - |
|  | TEAFIPFSLGK | 2 | - | - | - | - | - | - | 3.63 | - |
|  |  |  | 0 | 0 | 0 | 0 | 0 | 0 | 12 |  |
| cytochrome P450 2C19, NP_000760.1 | |  |  |  |  |  |  |  |  |  |
|  | M |  | - | - | - | - | - | - | 0.97 |  |
|  | ASPCDPTFILGCAPCNVICSIIFQK | 3 | - | - | - | - | - | - | 6.99 | - |
|  | CLVEELR | 2 | - | - | - | - | - | - | 2.77 | NP_000761.3, NP_000762.2 |
|  | DFIDCFLIK | 2 | - | - | - | - | - | - | 3.22 | NP_000761.3 |
|  | DLDTTPVVNGFASVPPFYQLCFIPV | 2 | - | - | - | - | - | - | 3.69 | - |
|  | DQQFLNLMEK | 2 | - | - | - | - | - | - | 3.52 | NP_000762.2 |
|  | EALIDLGEEFSGR | 2 | - | - | - | - | - | - | 4.77 | NP_000762.2 |
|  | EHQESMDINNPR | 3 | - | - | - | - | - | - | 4.11 | - |
|  | FDYKDQQFLNLMEK | 3 | - | - | - | - | - | - | 5.35 | NP_000762.2 |
|  | FSLMTLR | 2 | - | - | - | - | - | - | 2.53 | NP_000762.2 |
|  | GFGIVFSNGK | 2 | - | - | - | - | - | - | 2.63 | NP_000762.2 |
|  | GHFPLAER | 2 | - | - | - | - | - | - | 2.33 | - |
|  | GHMPYTDAVVHEVQR | 3 | - | - | - | - | - | - | 4.34 | - |
|  | HFLDEGGNFKK | 3 | - | - | - | - | - | - | 4.60 | NP_000762.2 |
|  | ICVGEGLAR | 2 | - | - | - | - | - | - | 3.26 | - |
|  | NLAFMESDILEK | 2 | - | - | - | - | - | - | 4.30 | - |
|  | NRSPCMQDR | 2 | - | - | - | - | - | - | 3.14 | NP_000762.2 |
|  | NYLIPK | 1 | - | - | - | - | - | - | 2.07 | NP_000761.3, NP_000762.2 |
|  | RFSLMTLR | 2 | - | - | - | - | - | - | 3.11 | NP_000762.2 |
|  | SLTNLSK | 1 | - | - | - | - | - | - | 1.87 | NP_000762.2 |
|  | SNYFMPFSAGK | 2 | - | - | - | - | - | - | 3.17 | - |
|  | VKEHQESMDINNPR | 3 | - | - | - | - | - | - | 5.91 | - |
|  | VQEEIER | 2 | - | - | - | - | - | - | 2.76 | NP_000762.2, NP_000762.2 |
|  | YALLLLLK | 2 | - | - | - | - | - | - | 2.42 | NP_000762.2 |
|  | YFMPFSAGK | 2 | - | - | - | - | - | - | 2.41 | NP_000761.3, NP_000762.2 |
|  | YIDLIPTSLPHAVTCDVK | 3 | - | - | - | - | - | - | 2.95 | - |
|  |  |  | 0 | 0 | 0 | 0 | 0 | 0 | 25 |  |
| cytochrome P450 2C8, NP_000761.3 | |  |  |  |  |  |  |  |  |  |
|  | M |  | - | - | - | - | - | - | 0.98 |  |
|  | ASPCDPTFILGCAPCNVICSVVFQK | 3 | - | - | - | - | - | - | 6.60 | - |
|  | CLVEELR | 2 | - | - | - | - | - | - | 2.77 | NP_000762.2, NP_000760.1 |
|  | DFIDCFLIK | 2 | - | - | - | - | - | - | 3.22 | NP_000760.1 |
|  | DNQKSEFNIENLVGTVADLFVAGTETTSTTLR | 3 | - | - | - | - | - | - | 2.62 | - |
|  | DQNFLTLMK | 2 | - | - | - | - | - | - | 3.42 | - |
|  | EALIDNGEEFSGR | 2 | - | - | - | - | - | - | 4.37 | - |
|  | EHQASLDVNNPR | 2 | - | - | - | - | - | - | 4.41 | - |
|  | FNENFR | 2 | - | - | - | - | - | - | 2.17 | - |
|  | FSLTTLR | 2 | - | - | - | - | - | - | 2.32 | NP_000764.1 |
|  | GIVSLPPSYQICFIPV | 2 | - | - | - | - | - | - | 3.79 | - |
|  | GLGIISSNGK | 2 | - | - | - | - | - | - | 2.43 | - |
|  | GLGIISSNGKR | 2 | - | - | - | - | - | - | 2.48 | - |
|  | ICAGEGLAR | 2 | - | - | - | - | - | - | 2.97 | - |
|  | KSDYFMPFSAGK | 2 | - | - | - | - | - | - | 2.92 | - |
|  | LPPGPTPLPIIGNMLQIDVK | 2 | - | - | - | - | - | - | 4.59 | - |
|  | NLNTTAVTK | 2 | - | - | - | - | - | - | 2.87 | - |
|  | NYLIPK | 1 | - | - | - | - | - | - | 2.07 | NP_000762.2, NP_000760.1 |
|  | RFNENFR | 2 | - | - | - | - | - | - | 2.89 | - |
|  | RFSLTTLR | 2 | - | - | - | - | - | - | 3.48 | NP_000764.1 |
|  | RICAGEGLAR | 2 | - | - | - | - | - | - | 3.31 | - |
|  | SDYFMPFSAGK | 2 | - | - | - | - | - | - | 3.84 | - |
|  | SHMPYTDAVVHEIQR | 3 | - | - | - | - | - | - | 5.00 | - |
|  | VKEHQASLDVNNPR | 3 | - | - | - | - | - | - | 5.91 | - |
|  | VQEEAHCLVEELR | 3 | - | - | - | - | - | - | 3.40 | - |
|  | VQEEAHCLVEELRK | 3 | - | - | - | - | - | - | 3.34 | - |
|  | VQEEIDHVIGR | 2 | - | - | - | - | - | - | 4.53 | - |
|  | YFMPFSAGK | 2 | - | - | - | - | - | - | 2.41 | NP_000762.2, NP_000760.1 |
|  | YGLLLLLK | 2 | - | - | - | - | - | - | 3.30 | - |
|  | YSDLVPTGVPHAVTTDTK | 2 | - | - | - | - | - | - | 5.30 | - |
|  |  |  | 0 | 0 | 0 | 0 | 0 | 0 | 29 |  |
| cytochrome P450 2C9, NP_000762.2 | |  |  |  |  |  |  |  |  |  |
|  | M |  | - | - | - | - | - | - | 0.96 |  |
|  | ASPCDPTFILGCAPCNVICSIIFHK | 3 | - | - | - | - | - | - | 2.65 | - |
|  | CLVEELR | 2 | - | - | - | - | - | - | 2.77 | NP_000761.3, NP_000760.1 |
|  | DQQFLNLMEK | 2 | - | - | - | - | - | - | 3.52 | NP_000760.1 |
|  | EALIDLGEEFSGR | 2 | - | - | - | - | - | - | 4.77 | NP_000760.1 |
|  | EALIDLGEEFSGRGIFPLAERANR | 3 | - | - | - | - | - | - | 2.98 | - |
|  | EHQESMDMNNPQDFIDCFLMK | 3 | - | - | - | - | - | - | 5.19 | - |
|  | FDYKDQQFLNLMEK | 3 | - | - | - | - | - | - | 5.35 | NP_000760.1 |
|  | FSLMTLR | 2 | - | - | - | - | - | - | 2.53 | NP_000760.1 |
|  | GFGIVFSNGK | 2 | - | - | - | - | - | - | 2.63 | NP_000760.1 |
|  | GFGIVFSNGKK | 2 | - | - | - | - | - | - | 2.45 | - |
|  | GIFPLAER | 2 | - | - | - | - | - | - | 2.07 | - |
|  | HFLDEGGNFKK | 3 | - | - | - | - | - | - | 4.60 | NP_000760.1 |
|  | HNQPSEFTIESLENTAVDLFGAGTETTSTTLR | 3 | - | - | - | - | - | - | 6.71 | - |
|  | LPPGPTPLPVIGNILQIGIK | 2 | - | - | - | - | - | - | 5.25 | - |
|  | NLDTTPVVNGFASVPPFYQLCFIPV | 2 | - | - | - | - | - | - | 3.21 | - |
|  | NRSPCMQDR | 2 | - | - | - | - | - | - | 3.14 | NP_000760.1 |
|  | NYLIPK | 1 | - | - | - | - | - | - | 2.07 | NP_000761.3, NP_000760.1 |
|  | RFSLMTLR | 2 | - | - | - | - | - | - | 3.11 | NP_000760.1 |
|  | SHMPYTDAVVHEVQR | 3 | - | - | - | - | - | - | 4.53 | - |
|  | SLTNLSK | 1 | - | - | - | - | - | - | 1.87 | NP_000760.1 |
|  | SYILEK | 1 | - | - | - | - | - | - | 2.24 | - |
|  | VQEEIER | 2 | - | - | - | - | - | - | 2.76 | NP_000760.1, NP_000760.1 |
|  | YALLLLLK | 2 | - | - | - | - | - | - | 2.42 | NP_000760.1 |
|  | YFMPFSAGK | 2 | - | - | - | - | - | - | 2.41 | NP_000761.3, NP_000760.1 |
|  | YIDLLPTSLPHAVTCDIK | 2 | - | - | - | - | - | - | 5.04 | - |
|  |  |  | 0 | 0 | 0 | 0 | 0 | 0 | 25 |  |
| cytochrome P450 2D6, NP_001020332.1, NP_000097.2 | |  |  |  |  |  |  |  |  |  |
|  | M |  | - | - | - | - | - | - | 0.97 |  |
|  | ACLGEPLAR | 2 | - | - | - | - | - | - | 2.68 | - |
|  | AFLTQLDELLTEHR | 3 | - | - | - | - | - | - | 3.16 | - |
|  | AVSNVIASLTCGR | 2 | - | - | - | - | - | - | 5.11 | - |
|  | DIEVQGFR | 2 | - | - | - | - | - | - | 3.08 | - |
|  | DLTEAFLAEMEK | 2 | - | - | - | - | - | - | 5.11 | - |
|  | EALVTHGEDTADRPPVPITQILGFGPR | 3 | - | - | - | - | - | - | 4.72 | - |
|  | EVLNAVPVLLHIPALAGK | 2 | - | - | - | - | - | - | 4.65 | - |
|  | FGDIVPLGVTHMTSR | 3 | - | - | - | - | - | - | 4.16 | - |
|  | FSVSTLR | 2 | - | - | - | - | - | - | 2.17 | - |
|  | GTTLITNLSSVLK | 2 | - | - | - | - | - | - | 4.18 | - |
|  | LLDLAQEGLKEESGFLR | 2 | - | - | - | - | - | - | 5.09 | - |
|  | MTWDPAQPPR | 2 | - | - | - | - | - | - | 3.09 | - |
|  | RFSVSTLR | 2 | - | - | - | - | - | - | 3.34 | - |
|  | RVQQEIDDVIGQVR | 3 | - | - | - | - | - | - | 3.12 | - |
|  | SQGVFLAR | 2 | - | - | - | - | - | - | 2.46 | - |
|  | VQQEIDDVIGQVR | 2 | - | - | - | - | - | - | 5.18 | - |
|  |  |  | 0 | 0 | 0 | 0 | 0 | 0 | 16 |  |
| cytochrome P450 2E1, NP_000764.1 | |  |  |  |  |  |  |  |  |  |
|  | M |  | - | - | - | - | - | - | 0.97 |  |
|  | DIDLSPIHIGFGCIPPR | 3 | - | - | - | - | - | - | 4.97 | - |
|  | DLTDCLLVEMEK | 2 | - | - | - | - | - | - | 4.63 | - |
|  | DLTDCLLVEMEKEK | 2 | - | - | - | - | - | - | 4.94 | - |
|  | DRQEMPYMDAVVHEIQR | 3 | - | - | - | - | - | - | 6.12 | - |
|  | EAHFLLEALR | 3 | - | - | - | - | - | - | 2.67 | - |
|  | EAHFLLEALRK | 3 | - | - | - | - | - | - | 2.89 | - |
|  | EALLDYKDEFSGR | 2 | - | - | - | - | - | - | 4.18 | - |
|  | EHHQSLDPNCPR | 3 | - | - | - | - | - | - | 3.41 | - |
|  | FITLVPSNLPHEATR | 2 | - | - | - | - | - | - | 4.26 | - |
|  | FKPEHFLNENGK | 3 | - | - | - | - | - | - | 2.95 | - |
|  | FSLTTLR | 2 | - | - | - | - | - | - | 2.32 | NP_000761.3 |
|  | GDLPAFHAHR | 2 | - | - | - | - | - | - | 2.30 | - |
|  | GIIFNNGPTWK | 2 | - | - | - | - | - | - | 3.59 | - |
|  | GTVVVPTLDSVLYDNQEFPDPEK | 3 | - | - | - | - | - | - | 6.44 | - |
|  | HFDYNDEK | 2 | - | - | - | - | - | - | 2.78 | - |
|  | IPAIKDR | 2 | - | - | - | - | - | - | 2.20 | - |
|  | KHFDYNDEK | 2 | - | - | - | - | - | - | 2.29 | - |
|  | LHEEIDR | 2 | - | - | - | - | - | - | 2.44 | - |
|  | QEMPYMDAVVHEIQR | 2 | - | - | - | - | - | - | 4.48 | - |
|  | RFSLTTLR | 2 | - | - | - | - | - | - | 3.48 | NP_000761.3 |
|  | RVCAGEGLAR | 2 | - | - | - | - | - | - | 2.77 | - |
|  | VCAGEGLAR | 2 | - | - | - | - | - | - | 3.23 | - |
|  | VKEHHQSLDPNCPR | 3 | - | - | - | - | - | - | 4.61 | - |
|  | YPEIEEK | 2 | - | - | - | - | - | - | 2.10 | - |
|  | YSDYFKPFSTGK | 2 | - | - | - | - | - | - | 4.37 | - |
|  |  |  | 0 | 0 | 0 | 0 | 0 | 0 | 25 |  |
| cytochrome P450 2J2, NP_000766.2 | |  |  |  |  |  |  |  |  |  |
|  | M |  | - | - | - | - | - | - | 0.94 |  |
|  | TCQLYNVFPWIMK | 2 | - | - | - | - | - | - | 3.23 | - |
|  | VIGQGQQPSTAAR | 2 | - | - | - | - | - | - | 4.57 | - |
|  |  |  | 0 | 0 | 0 | 0 | 0 | 0 | 2 |  |
| cytochrome P450 3A4, NP_073731.1, NP_476437.1, NP_476436.1, NP_059488.2 | | | |  |  |  |  |  |  |  |
|  | M |  | - | - | - | - | - | - | 0.97 |  |
|  | APPTYDTVLQMEYLDMVVNETLR | 3 | - | - | - | - | - | - | 5.73 | - |
|  | DNIDPYIYTPFGSGPR | 2 | - | - | - | - | - | - | 5.37 | - |
|  | DVEINGMFIPK | 2 | - | - | - | - | - | - | 4.08 | - |
|  | EAETGKPVTLK | 2 | - | - | - | - | - | - | 3.34 | - |
|  | ECYSVFTNR | 2 | - | - | - | - | - | - | 3.21 | NP_000768.1 |
|  | EMVPIIAQYGDVLVR | 3 | - | - | - | - | - | - | 5.45 | - |
|  | ETQIPLK | 1 | - | - | - | - | - | - | 1.93 | NP_000768.1 |
|  | EVTNFLR | 1 | - | - | - | - | - | - | 2.11 | - |
|  | FALMNMK | 2 | - | - | - | - | - | - | 2.66 | NP_000768.1 |
|  | GVVVMIPSYALHR | 2 | - | - | - | - | - | - | 3.52 | - |
|  | KDVEINGMFIPK | 3 | - | - | - | - | - | - | 4.12 | - |
|  | LKEMVPIIAQYGDVLVR | 2 | - | - | - | - | - | - | 5.20 | - |
|  | LQEEIDAVLPNK | 2 | - | - | - | - | - | - | 4.07 | - |
|  | LSLGGLLQPEKPVVLK | 2 | - | - | - | - | - | - | 4.55 | - |
|  | NKDNIDPYIYTPFGSGPR | 3 | - | - | - | - | - | - | 5.76 | - |
|  | REAETGKPVTLK | 3 | - | - | - | - | - | - | 4.11 | - |
|  | SAISIAEDEEWK | 2 | - | - | - | - | - | - | 4.11 | - |
|  | SAISIAEDEEWKR | 2 | - | - | - | - | - | - | 3.91 | - |
|  | SLLSPTFTSGK | 2 | - | - | - | - | - | - | 2.77 | NP_000768.1 |
|  | VDFLQLMIDSQNSK | 2 | - | - | - | - | - | - | 5.65 | - |
|  | VLQNFSFKPCK | 2 | - | - | - | - | - | - | 3.35 | NP_000768.1 |
|  | VWGFYDGQQPVLAITDPDMIK | 3 | - | - | - | - | - | - | 6.60 | - |
|  | YWTEPEK | 1 | - | - | - | - | - | - | 2.28 | - |
|  |  |  | 0 | 0 | 0 | 0 | 0 | 0 | 23 |  |
| cytochrome P450 3A5, NP_000768.1 | |  |  |  |  |  |  |  |  |  |
|  | M |  | - | - | - | - | - | - | 0.94 |  |
|  | DSIDPYIYTPFGTGPR | 2 | - | - | - | - | - | - | 4.85 | - |
|  | ECYSVFTNR | 2 | - | - | - | - | - | - | 3.21 | NP_059488.2 |
|  | ETQIPLK | 1 | - | - | - | - | - | - | 1.93 | NP_073731.1, NP_059488.2, NP_476436.1 |
|  | FALMNMK | 2 | - | - | - | - | - | - | 2.66 | NP_059488.2 |
|  | LDTQGLLQPEKPIVLK | 3 | - | - | - | - | - | - | 2.59 | - |
|  | SLLSPTFTSGK | 2 | - | - | - | - | - | - | 2.77 | NP_059488.2 |
|  | VLQNFSFKPCK | 2 | - | - | - | - | - | - | 3.35 | NP_059488.2 |
|  |  |  | 0 | 0 | 0 | 0 | 0 | 0 | 7 |  |
| cytochrome P450 4A11, NP_000769.2 | |  |  |  |  |  |  |  |  |  |
|  | M |  | - | - | - | - | - | - | 0.97 |  |
|  | ACQLAHQHTDQVIQLR | 3 | - | - | - | - | - | - | 5.12 | - |
|  | CAFSHQGSIQVDR | 3 | - | - | - | - | - | - | 4.81 | - |
|  | ELSTPVTFPDGR | 2 | - | - | - | - | - | - | 3.43 | - |
|  | FELLPDPTRIPIPIAR | 3 | - | - | - | - | - | - | 2.80 | - |
|  | LPNPCEDKDQL | 2 | - | - | - | - | - | - | 4.07 | - |
|  | MENGSILSDKDLR | 3 | - | - | - | - | - | - | 3.10 | - |
|  | NSQSYIQAISDLNNLVFSR | 2 | - | - | - | - | - | - | 6.39 | - |
|  | QFAMNELK | 2 | - | - | - | - | - | - | 2.34 | - |
|  | RLPNPCEDKDQL | 3 | - | - | - | - | - | - | 2.82 | - |
|  | VATALTLLR | 2 | - | - | - | - | - | - | 3.39 | - |
|  | VQLYDPDYMK | 2 | - | - | - | - | - | - | 3.47 | - |
|  | VWPNPEVFDPFR | 2 | - | - | - | - | - | - | 3.17 | - |
|  |  |  | 0 | 0 | 0 | 0 | 0 | 0 | 12 |  |
| cytochrome P450 4F12, NP_076433.2 | |  |  |  |  |  |  |  |  |  |
|  | M |  | - | - | - | - | - | - | 0.97 |  |
|  | AEGGLWLR | 2 | - | - | - | - | - | - | 3.07 | - |
|  | CCTQDIVLPDGR | 2 | - | - | - | - | - | - | 4.30 | - |
|  | LDMFEHISLMTLDSLQK | 3 | - | - | - | - | - | - | 4.78 | - |
|  | LQCFPQPPK | 2 | - | - | - | - | - | - | 2.85 | - |
|  | LSDEDIR | 2 | - | - | - | - | - | - | 2.23 | - |
|  | NCIGQAFAMAEMK | 2 | - | - | - | - | - | - | 4.77 | - |
|  | QEVQELLK | 2 | - | - | - | - | - | - | 2.56 | - |
|  | SPLAFIPFSAGPR | 2 | - | - | - | - | - | - | 3.97 | - |
|  | TLDFIDVLLLSK | 2 | - | - | - | - | - | - | 5.38 | - |
|  | TLDFIDVLLLSKDEDGK | 2 | - | - | - | - | - | - | 5.52 | - |
|  |  |  | 0 | 0 | 0 | 0 | 0 | 0 | 10 |  |
| dimethylaniline monooxygenase [N-oxide-forming] 3, NP_001002294.1, NP_008825.4 | | | |  |  |  |  |  |  |  |
|  | M |  | - | - | - | - | - | - | 0.98 |  |
|  | DYKEPGVFNGK | 2 | - | - | - | - | - | - | 3.12 | - |
|  | EMMCFPDFPFPDDFPNFMHNSK | 3 | - | - | - | - | - | - | 3.64 | - |
|  | EPGVFNGKRVLVVGLGNSGCDIATELSR | 3 | - | - | - | - | - | - | 2.82 | - |
|  | EPVFNDELPASILCGIVSVKPNVK | 3 | - | - | - | - | - | - | 5.10 | - |
|  | ESAVFDAVMVCSGHHVYPNLPK | 3 | - | - | - | - | - | - | 3.89 | - |
|  | ESFPGLNHFK | 2 | - | - | - | - | - | - | 2.81 | - |
|  | FSDHAEEGR | 2 | - | - | - | - | - | - | 3.27 | - |
|  | GTCTLPSMEDMMNDINEK | 2 | - | - | - | - | - | - | 2.05 | - |
|  | GVFPPLLEK | 2 | - | - | - | - | - | - | 2.12 | - |
|  | HPDFATTGQWDVTTER | 3 | - | - | - | - | - | - | 5.00 | - |
|  | IQEYIIAFAK | 2 | - | - | - | - | - | - | 4.33 | - |
|  | KEPVFNDELPASILCGIVSVKPNVK | 3 | - | - | - | - | - | - | 7.44 | - |
|  | LVGPGQWPGAR | 2 | - | - | - | - | - | - | 3.11 | - |
|  | NAILTQWDR | 2 | - | - | - | - | - | - | 3.49 | - |
|  | NNEIILFK | 2 | - | - | - | - | - | - | 3.17 | - |
|  | NNLPTAISDWLYVK | 2 | - | - | - | - | - | - | 4.78 | - |
|  | PNIPWLFLTDPK | 2 | - | - | - | - | - | - | 4.41 | - |
|  | SCLEEGLEPTCFEK | 2 | - | - | - | - | - | - | 5.48 | - |
|  | SETIQTDYIVYMDELSSFIGAK | 2 | - | - | - | - | - | - | 6.52 | - |
|  | SGSWVMSR | 2 | - | - | - | - | - | - | 2.93 | - |
|  | SLKPMQTR | 2 | - | - | - | - | - | - | 2.07 | - |
|  | SNDIGGLWK | 2 | - | - | - | - | - | - | 3.56 | - |
|  | SRNNEIILFK | 3 | - | - | - | - | - | - | 3.25 | - |
|  | STIAVIGFVQSLGAAIPTVDLQSR | 3 | - | - | - | - | - | - | 6.33 | - |
|  | TAEQVMISSR | 2 | - | - | - | - | - | - | 4.23 | - |
|  | TFVSSVNK | 2 | - | - | - | - | - | - | 2.55 | - |
|  | TFVSSVNKHPDFATTGQWDVTTER | 3 | - | - | - | - | - | - | 6.21 | - |
|  | VAIIGAGVSGLASIR | 2 | - | - | - | - | - | - | 4.96 | - |
|  | VLVVGLGNSGCDIATELSR | 2 | - | - | - | - | - | - | 6.52 | - |
|  | VWDNGYPWDMLLVTR | 2 | - | - | - | - | - | - | 5.42 | - |
|  | WAAQVIK | 2 | - | - | - | - | - | - | 2.03 | - |
|  |  |  | 0 | 0 | 0 | 0 | 0 | 0 | 31 |  |
| dimethylaniline monooxygenase [N-oxide-forming] 5, NP_001138301.1, NP_001452.2 | | | |  |  |  |  |  |  |  |
|  | M |  | - | - | - | - | - | - | 0.98 |  |
|  | ALSQHPTLNDDLPNR | 3 | - | - | - | - | - | - | 5.21 | - |
|  | DYKNPEGFTGK | 2 | - | - | - | - | - | - | 3.42 | - |
|  | DYKNPEGFTGKR | 3 | - | - | - | - | - | - | 3.81 | - |
|  | EFDLLK | 1 | - | - | - | - | - | - | 2.22 | - |
|  | EFTETAAIFEDGSR | 2 | - | - | - | - | - | - | 5.25 | - |
|  | FDHEMFGLKPK | 3 | - | - | - | - | - | - | 4.14 | - |
|  | FQENPEEGR | 2 | - | - | - | - | - | - | 3.09 | - |
|  | IAVIGGGVSGLSSIK | 2 | - | - | - | - | - | - | 5.58 | - |
|  | ICGQSLANK | 2 | - | - | - | - | - | - | 2.88 | - |
|  | KQPDFATSGQWEVVTESEGK | 3 | - | - | - | - | - | - | 5.63 | - |
|  | KQPDFATSGQWEVVTESEGKK | 3 | - | - | - | - | - | - | 3.84 | - |
|  | NPEGFTGKR | 2 | - | - | - | - | - | - | 2.98 | - |
|  | QPDFATSGQWEVVTESEGKK | 3 | - | - | - | - | - | - | 4.40 | - |
|  | RGAWILNR | 2 | - | - | - | - | - | - | 2.98 | - |
|  | TDDIGGLWR | 2 | - | - | - | - | - | - | 3.41 | - |
|  | TLPSQSEMMAEISK | 2 | - | - | - | - | - | - | 4.25 | - |
|  | VGDYGYPADVLFSSR | 2 | - | - | - | - | - | - | 5.07 | - |
|  | VIIIGIGNSGGDLAVEISQTAK | 2 | - | - | - | - | - | - | 6.54 | - |
|  | WATQVFK | 2 | - | - | - | - | - | - | 2.46 | - |
|  |  |  | 0 | 0 | 0 | 0 | 0 | 0 | 19 |  |
| electron transfer flavoprotein-ubiquinone oxidoreductase, NP_004444.2 | | | |  |  |  |  |  |  |  |
|  | M |  | - | - | - | - | - | - | 0.98 |  |
|  | AAQIGAHTLSGACLDPGAFK | 3 | - | - | - | - | - | - | 5.55 | - |
|  | ALNEGGFQSIPK | 2 | - | - | - | - | - | - | 4.18 | - |
|  | ANCEPQTYGIGLK | 2 | - | - | - | - | - | - | 3.98 | - |
|  | DDSIPVNR | 2 | - | - | - | - | - | - | 2.58 | - |
|  | ELFPDWK | 2 | - | - | - | - | - | - | 2.09 | - |
|  | ELWVIDEK | 1 | - | - | - | - | - | - | 2.93 | - |
|  | FCPAGVYEFVPVEQGDGFR | 2 | - | - | - | - | - | - | 5.92 | - |
|  | FGILTEK | 2 | - | - | - | - | - | - | 2.52 | - |
|  | GAPLNTPVTEDR | 2 | - | - | - | - | - | - | 3.63 | - |
|  | GIATNDVGIQK | 2 | - | - | - | - | - | - | 3.79 | - |
|  | GMEPWTLK | 2 | - | - | - | - | - | - | 2.43 | - |
|  | IPVPILPGLPMNNHGNYIVR | 3 | - | - | - | - | - | - | 3.58 | - |
|  | ITTHYTIYPR | 2 | - | - | - | - | - | - | 3.57 | - |
|  | LKQLAVAHEK | 2 | - | - | - | - | - | - | 3.41 | - |
|  | LQINAQNCVHCK | 2 | - | - | - | - | - | - | 3.89 | - |
|  | LTFPGGLLIGCSPGFMNVPK | 2 | - | - | - | - | - | - | 5.14 | - |
|  | NLSIYDGPEQR | 2 | - | - | - | - | - | - | 3.54 | - |
|  | NSWVWK | 2 | - | - | - | - | - | - | 2.40 | - |
|  | SGILAAESIFNQLTSENLQSK | 3 | - | - | - | - | - | - | 7.07 | - |
|  | TIGLHVTEYEDNLK | 2 | - | - | - | - | - | - | 4.38 | - |
|  | VDHTVGWPLDR | 3 | - | - | - | - | - | - | 2.93 | - |
|  | VTIFAEGCHGHLAK | 2 | - | - | - | - | - | - | 4.02 | - |
|  | WEGVNMER | 2 | - | - | - | - | - | - | 2.49 | - |
|  |  |  | 0 | 0 | 0 | 0 | 0 | 0 | 23 |  |
| epoxide hydrolase 1, NP_001129490.1, NP_000111.1 | |  |  |  |  |  |  |  |  |  |
|  | M |  | - | - | - | - | - | 0.96 | 0.98 |  |
|  | DKEETLPLEDGWWGPGTR | 3 | - | - | - | - | - | 6.35 | 5.98 | - |
|  | DVELLYPVK | 2 | - | - | - | - | - | 2.95 | 3.24 | - |
|  | DVELLYPVKEK | 2 | - | - | - | - | - | - | 3.68 | - |
|  | EDDSIRPFK | 2 | - | - | - | - | - | 2.23 | 2.61 | - |
|  | EDDSIRPFKVETSDEEIHDLHQR | 3 | - | - | - | - | - | - | 5.29 | - |
|  | EETLPLEDGWWGPGTR | 3 | - | - | - | - | - | 3.29 | 5.42 | - |
|  | EKVFYSLMR | 2 | - | - | - | - | - | - | 2.58 | - |
|  | ENLGQGWMTQK | 2 | - | - | - | - | - | 3.07 | 3.61 | - |
|  | FLGLTER | 2 | - | - | - | - | - | 2.27 | 2.84 | - |
|  | FLGLTERDVELLYPVK | 3 | - | - | - | - | - | - | 5.08 | - |
|  | FLSVLER | 2 | - | - | - | - | - | 2.49 | 2.74 | - |
|  | FLSVLERQ | 2 | - | - | - | - | - | - | 3.35 | - |
|  | FSTWTNTEFR | 2 | - | - | - | - | - | 2.39 | 3.31 | - |
|  | FTPPLEDSCFHYGFNSNYLK | 2 | - | - | - | - | - | - | 4.22 | - |
|  | FYKENLGQGWMTQK | 3 | - | - | - | - | - | - | 5.13 | - |
|  | GFNSVATAR | 2 | - | - | - | - | - | - | 3.35 | - |
|  | GGHFAAFEEPELLAQDIR | 2 | - | - | - | - | - | 4.41 | 6.47 | - |
|  | GGHFAAFEEPELLAQDIRK | 3 | - | - | - | - | - | - | 5.46 | - |
|  | IIPLLTDPK | 1 | - | - | - | - | - | 2.01 | 2.65 | - |
|  | KFLSVLER | 2 | - | - | - | - | - | - | 3.06 | - |
|  | KGFNSVATAR | 2 | - | - | - | - | - | 3.16 | 3.77 | - |
|  | KQVEILNR | 2 | - | - | - | - | - | - | 2.70 | - |
|  | KVISYWR | 2 | - | - | - | - | - | - | 2.78 | - |
|  | LISYSYMVR | 2 | - | - | - | - | - | 2.35 | 2.89 | - |
|  | NEFDWK | 1 | - | - | - | - | - | - | 2.37 | - |
|  | NEFDWKK | 2 | - | - | - | - | - | - | 2.56 | - |
|  | NHGLSDEHVFEVICPSIPGYGFSEASSK | 3 | - | - | - | - | - | 2.73 | 6.97 | - |
|  | QVEILNR | 1 | - | - | - | - | - | - | 1.79 | - |
|  | VETSDEEIHDLHQR | 2 | - | - | - | - | - | - | 4.66 | - |
|  | VFYSLMR | 2 | - | - | - | - | - | - | 3.17 | - |
|  | VISYWR | 2 | - | - | - | - | - | - | 2.03 | - |
|  | VYVPTGFSAFPFELLHTPEK | 2 | - | - | - | - | - | - | 4.87 | - |
|  | YLEDGGLER | 2 | - | - | - | - | - | 3.14 | 3.41 | - |
|  |  |  | 0 | 0 | 0 | 0 | 0 | 14 | 33 |  |
| epoxide hydrolase 2, NP_001970.2 | |  |  |  |  |  |  |  |  |  |
|  | C |  | - | - | - | - | - | - | 0.98 |  |
|  | AAVFDLDGVLALPAVFGVLGR | 2 | - | - | - | - | - | - | 5.06 | - |
|  | ASPSEVVFLDDIGANLKPAR | 2 | - | - | - | - | - | - | 6.14 | - |
|  | AVASLNTPFIPANPNMSPLESIK | 2 | - | - | - | - | - | - | 5.43 | - |
|  | DGLAQLMCELK | 2 | - | - | - | - | - | - | 3.95 | - |
|  | DLGMVTILVQDTDTALK | 2 | - | - | - | - | - | - | 2.63 | - |
|  | GFTTAILTNTWLDDR | 2 | - | - | - | - | - | - | 4.56 | - |
|  | GGPEGATTR | 2 | - | - | - | - | - | - | 2.58 | - |
|  | GLLNDAFQK | 2 | - | - | - | - | - | - | 2.92 | - |
|  | ILIPALMVTAEK | 2 | - | - | - | - | - | - | 3.15 | - |
|  | MVTEEEIQFYVQQFK | 2 | - | - | - | - | - | - | 3.99 | - |
|  | TEEALALPR | 2 | - | - | - | - | - | - | 3.57 | - |
|  | VCEAGGLFVNSPEEPSLSR | 2 | - | - | - | - | - | - | 6.62 | - |
|  | YQIPALAQAGYR | 2 | - | - | - | - | - | - | 3.65 | - |
|  |  |  | 0 | 0 | 0 | 0 | 0 | 0 | 13 |  |
| gamma-glutamyl carboxylase, NP_001135741.1, NP_000812.2 | | |  |  |  |  |  |  |  |  |
|  | M |  | - | - | - | - | - | - | 0.95 |  |
|  | AAPQPSVSCVYK | 2 | - | - | - | - | - | - | 2.20 | - |
|  | DKAELISGPR | 2 | - | - | - | - | - | - | 3.22 | - |
|  | GGPEPTPLVQTFLR | 2 | - | - | - | - | - | - | 3.51 | - |
|  | LQQLLPLK | 2 | - | - | - | - | - | - | 2.85 | - |
|  | TGELGYLNPGVFTQSR | 2 | - | - | - | - | - | - | 5.16 | - |
|  | VDIVQAAWSPFQR | 2 | - | - | - | - | - | - | 4.19 | - |
|  | YLDGLDVCR | 2 | - | - | - | - | - | - | 3.66 | - |
|  |  |  | 0 | 0 | 0 | 0 | 0 | 0 | 7 |  |
| gamma-glutamyltransferase 5, NP_001093252.1, NP_004112.2, NP_001093251.1 | | | |  |  |  |  |  |  |  |
|  | M |  | - | - | - | - | - | 0.96 | 0.96 |  |
|  | DLLGETLAQLIR | 2 | - | - | - | - | - | 3.67 | 4.13 | - |
|  | EGSQLTLQDLAK | 2 | - | - | - | - | - | 2.87 | - | - |
|  | GCVEYEPNFSQEVQR | 2 | - | - | - | - | - | 5.30 | - | - |
|  | GGHVVAPVLSR | 2 | - | - | - | - | - | 2.30 | - | - |
|  | LGQMLVEDIAK | 2 | - | - | - | - | - | 4.21 | 3.90 | - |
|  | LQNASRDLLGETLAQLIR | 3 | - | - | - | - | - | 2.50 | - | - |
|  | SPSSMVPSILINK | 2 | - | - | - | - | - | 4.05 | - | - |
|  | TGIILNNELLDLCER | 2 | - | - | - | - | - | 4.84 | - | - |
|  |  |  | 0 | 0 | 0 | 0 | 0 | 8 | 2 |  |
| glutamate--cysteine ligase catalytic subunit, NP_001489.1 | |  |  |  |  |  |  |  |  |  |
|  | C |  | - | - | - | - | - | - | 0.83 |  |
|  | LGCPGFTLPEVKPNPVEGGASK | 3 | - | - | - | - | - | - | 2.92 | - |
|  | VLETLQEK | 2 | - | - | - | - | - | - | 2.67 | - |
|  | VVINVPIFK | 2 | - | - | - | - | - | - | 2.89 | - |
|  |  |  | 0 | 0 | 0 | 0 | 0 | 0 | 3 |  |
| glutathione peroxidase 3, NP_002075.2 | |  |  |  |  |  |  |  |  |  |
|  | C |  | - | - | - | - | - | 0.92 | - |  |
|  | NSCPPTSELLGTSDR | 2 | - | - | - | - | - | 4.35 | - | - |
|  | PGGGFVPNFQLFEK | 2 | - | - | - | - | - | 4.27 | - | - |
|  | QEPGENSEILPTLK | 2 | - | - | - | - | - | 3.36 | - | - |
|  |  |  | 0 | 0 | 0 | 0 | 0 | 3 | 0 |  |
| glutathione S-transferase alpha, NP_665683.1, NP_001503.1, NP_714543.1, NP_000837.3, NP_000838.3 | | | | | |  |  |  |  |  |
|  | C |  | 0.97 | 0.96 | 0.92 | 0.96 | - | 0.88 | 0.98 |  |
|  | ADIHLVELLYYVEELDSSLISSFPLLK | 3 | - | - | - | - | - | 4.24 | - | - |
|  | AILNYIASK | 2 | - | - | - | - | - | 3.37 | 3.49 | - |
|  | FLQPGSPR | 2 | - | - | - | - | - | 2.14 | 2.37 | - |
|  | GRMESIR | 2 | - | - | - | - | - | - | 2.10 | - |
|  | IALIKEK | 2 | - | - | - | - | - | - | 2.28 | - |
|  | IPNILSAFPFLQEYTVK | 2 | 5.53 | 5.33 | 4.87 | 5.17 | - | - | - | - |
|  | ISNLPTVK | 2 | - | - | - | - | - | 2.17 | 3.07 | - |
|  | ISNLPTVKK | 2 | - | - | - | - | - | - | 3.14 | - |
|  | KFLQPGSPR | 2 | - | - | - | - | - | - | 2.81 | - |
|  | KKPPPDEIYVR | 3 | 3.41 | 3.34 | - | 3.83 | - | - | - | - |
|  | KPPMDEK | 2 | - | - | - | - | - | - | 2.17 | - |
|  | LHYFNAR | 2 | - | - | - | - | - | - | 2.56 | - |
|  | LHYSNIR | 2 | - | - | - | - | - | - | 2.17 | - |
|  | LRNDGYLMFQQVPMVEIDGMK | 3 | - | - | - | - | - | - | 5.29 | - |
|  | LSNIPTIK | 2 | 2.45 | 2.26 | - | 2.68 | - | - | - | - |
|  | NDGYLMFQQVPMVEIDGMK | 2 | - | - | - | - | - | - | 5.51 | - |
|  | PEEKDAK | 1 | - | - | - | - | - | - | 1.57 | - |
|  | SAEDLDK | 1 | - | - | - | - | - | - | 2.12 | - |
|  | SAEDLDKLR | 2 | - | - | - | - | - | 2.61 | 3.24 | - |
|  | SHGQDYLVGNK | 2 | - | - | - | - | - | 2.07 | 3.84 | - |
|  | SILHYIADK | 2 | 2.36 | - | - | 2.25 | - | - | - | - |
|  | SLEEAR | 1 | - | - | - | - | - | - | 1.96 | - |
|  | VLKSHGQDYLVGNK | 3 | - | - | - | - | - | - | 3.31 | - |
|  | VLQSHGQDYLVGNK | 3 | - | - | - | - | - | - | 2.85 | - |
|  | WLLAAAGVEFEEK | 2 | - | - | - | - | - | 2.98 | 5.20 | - |
|  | WVLAAAGVEFDEEFLETK | 2 | 2.71 | - | 3.15 | - | - | - | - | - |
|  | YFPAFEK | 1 | - | - | - | - | - | - | 2.23 | - |
|  | YNLYGK | 1 | - | - | - | - | - | - | 1.94 | - |
|  |  |  | 5 | 3 | 2 | 4 | 0 | 7 | 22 |  |
| glutathione S-transferase kappa, NP_001137151.1, NP_001137153.1, NP_001137152.1, NP_057001.1 | | | | |  |  |  |  |  |  |
|  | C |  | - | - | - | - | - | - | 0.97 |  |
|  | AGMSAEQAQGLLEK | 2 | - | - | - | - | - | - | 5.21 | - |
|  | DFLSVMLEK | 2 | - | - | - | - | - | - | 3.00 | - |
|  | DSGNKPPGLLPR | 2 | - | - | - | - | - | - | 3.13 | - |
|  | FLTAVNLEHPEMLEK | 2 | - | - | - | - | - | - | 4.73 | - |
|  | GLYMANDLK | 2 | - | - | - | - | - | - | 2.06 | - |
|  | KGLYMANDLK | 2 | - | - | - | - | - | - | 3.37 | - |
|  | MELLAHLLGEK | 2 | - | - | - | - | - | - | 3.79 | - |
|  | NEDITEPQSILAAAEK | 2 | - | - | - | - | - | - | 6.19 | - |
|  | WMGPIPPAVNAR | 2 | - | - | - | - | - | - | 2.13 | - |
|  |  |  | 0 | 0 | 0 | 0 | 0 | 0 | 9 |  |
| glutathione S-transferase mu, NP_671489.1, NP_001135840.1, NP_666533.1, NP_000552.2, NP_000840.2, NP_000842.2, NP_000841.1, NP_000839.1 | | | | | | | | |  |  |
|  | C |  | 0.97 | 0.97 | 0.97 | 0.97 | - | 0.97 | 0.98 |  |
|  | CLDAFPNLK | 2 | - | - | - | - | - | - | 3.41 | - |
|  | CLDAFPNLKDFISR | 2 | - | - | - | - | - | - | 4.42 | - |
|  | CLDEFPNLK | 2 | 3.32 | 2.84 | - | - | - | 2.79 | - | - |
|  | FEGLEK | 1 | - | - | - | - | - | - | 1.67 | - |
|  | FKLDLDFPNLPYLLDGK | 3 | 5.25 | 5.56 | 4.54 | 4.84 | - | 4.15 | - | - |
|  | FLPRPVFSK | 2 | - | - | - | - | - | - | 2.26 | - |
|  | FSWFAGEK | 2 | 2.50 | 2.64 | - | - | - | 2.52 | - | - |
|  | HNLCGETEEEK | 2 | - | - | - | - | - | - | 4.09 | - |
|  | HNLCGETEEEKIR | 3 | - | - | - | - | - | - | 5.81 | - |
|  | IAAYLQSDQFCK | 2 | 4.51 | 4.74 | - | - | - | 4.53 | 4.64 | - |
|  | IFEPNCLDAFPNLK | 2 | - | - | 4.33 | - | - | 2.04 | 4.59 | - |
|  | IFEPNCLDAFPNLKDFISR | 3 | - | - | - | - | - | - | 3.47 | - |
|  | ISAYMK | 1 | - | - | - | - | - | - | 1.56 | - |
|  | ITFVDFIAYDVLER | 2 | 5.06 | - | - | - | - | 3.39 | - | - |
|  | ITFVDFLVYDVLDLHR | 3 | 4.59 | 5.95 | 3.80 | - | - | - | - | - |
|  | ITQSNAILCYIAR | 3 | - | 3.85 | - | - | - | 4.23 | 4.93 | - |
|  | ITQSNAILR | 2 | 3.15 | 3.31 | 2.77 | - | - | 3.12 | 3.20 | - |
|  | KHNLCGETEEEK | 2 | 3.04 | - | - | - | - | 2.53 | 4.32 | - |
|  | KHNLCGETEEEKIR | 3 | - | - | - | - | - | - | 4.83 | - |
|  | KHNMCGETEEEK | 3 | - | - | - | - | - | 3.22 | 3.17 | - |
|  | KYTMGDAPDYDR | 2 | - | - | - | - | - | - | 4.54 | - |
|  | LCYDPDFEK | 2 | - | - | - | - | - | 2.54 | - | - |
|  | LDLDFPNLPYLLDGK | 2 | 4.37 | 4.62 | 4.23 | - | - | 4.28 | 4.01 | - |
|  | LGLDFPNLPYLIDGAHK | 2 | 2.98 | 4.47 | 4.38 | - | - | 2.64 | 4.93 | - |
|  | LGLDFPNLPYLIDGTHK | 3 | 4.33 | 3.97 | - | - | - | 3.14 | - | - |
|  | LKPQYLEELPGQLK | 3 | 4.45 | 4.33 | 3.63 | 3.54 | - | 3.70 | 4.24 | - |
|  | LLLEFTDTSYEEK | 2 | 4.89 | 4.69 | 2.56 | - | - | 4.77 | - | - |
|  | LLLEFTDTSYEEKR | 3 | - | - | - | - | - | 3.06 | - | - |
|  | LLLEYTDSSYEEK | 2 | 2.65 | 4.68 | - | - | - | 3.32 | 5.19 | - |
|  | LLLEYTDSSYEEKK | 2 | - | - | - | - | - | - | 4.25 | - |
|  | LTFVDFLTYDILDQNR | 2 | 5.88 | 6.01 | 5.85 | 5.83 | - | 2.64 | 5.70 | - |
|  | LYSEFLGK | 2 | 2.26 | 2.69 | 2.12 | - | - | 2.11 | 2.66 | - |
|  | LYSQFLGK | 2 | - | - | - | - | - | 2.47 | - | - |
|  | MAQWGNKPVC | 2 | - | - | - | - | - | 3.07 | - | - |
|  | MAVWGNK | 2 | - | - | - | - | - | - | 2.35 | - |
|  | RPWFAGDK | 2 | - | - | - | - | - | 2.35 | 2.78 | - |
|  | RPWFAGNK | 2 | - | - | - | - | - | 2.23 | 3.50 | - |
|  | RPWFVGDK | 2 | - | - | - | - | - | - | 2.22 | - |
|  | SQWLDVK | 2 | 2.17 | 2.39 | - | - | - | 2.31 | - | - |
|  | SQWLNEK | 2 | - | - | - | - | - | - | 2.64 | - |
|  | VDIIENQVMDFR | 2 | 2.40 | 3.66 | - | - | - | - | - | - |
|  | YLEELPEK | 2 | 2.56 | 2.97 | 2.71 | - | - | - | 3.26 | - |
|  | YLEELPEKLK | 2 | - | - | - | - | - | 2.40 | 3.20 | - |
|  | YTCGEAPDYDR | 2 | 3.06 | 3.20 | - | - | - | 3.52 | 3.07 | - |
|  | YTMGDAPDYDR | 2 | - | - | - | - | - | - | 3.40 | - |
|  |  |  | 20 | 19 | 11 | 3 | 0 | 27 | 32 |  |
| glutathione S-transferase omega, NP_899062.1, NP_004823.1 | | |  |  |  |  |  |  |  |  |
|  | C |  | 0.91 | 0.93 | 0.91 | 0.90 | 0.85 | 0.93 | 0.95 |  |
|  | EDPTVSALLTSEK | 2 | 3.64 | 3.33 | - | 3.52 | 3.16 | - | 3.38 | - |
|  | EFTKLEEVLTNKK | 3 | - | - | - | - | - | - | 3.41 | - |
|  | GSAPPGPVPEGSIR | 2 | 2.35 | 2.37 | 2.94 | 2.04 | 2.60 | 2.09 | 2.52 | - |
|  | HEVININLK | 2 | 2.24 | 2.85 | - | 2.93 | 2.97 | 2.92 | 3.29 | - |
|  | LEEVLTNK | 2 | 2.23 | 2.20 | 2.13 | - | 2.14 | - | 2.91 | - |
|  | LEEVLTNKK | 2 | - | - | - | - | - | - | 3.02 | - |
|  | LNECVDHTPK | 2 | - | 3.30 | 3.58 | 2.79 | 3.64 | - | 3.96 | - |
|  | MILELFSK | 2 | - | - | - | - | - | - | 2.63 | - |
|  | NKPEWFFK | 2 | - | - | - | - | - | - | 2.16 | - |
|  | SQNKEDYAGLKEEFR | 3 | - | - | - | - | - | - | 4.60 | - |
|  | VPSLVGSFIR | 2 | 3.39 | 3.49 | 3.49 | 3.31 | 3.24 | 3.39 | 3.59 | - |
|  |  |  | 5 | 6 | 4 | 5 | 6 | 3 | 11 |  |
| glutathione S-transferase pi, NP_000843.1 | |  |  |  |  |  |  |  |  |  |
|  | C |  | 0.98 | 0.98 | 0.98 | 0.98 | 0.98 | 0.98 | 0.98 |  |
|  | AFLASPEYVNLPINGNGK | 2 | 5.66 | 5.21 | 3.76 | 3.81 | 4.01 | 3.39 | - | - |
|  | AFLASPEYVNLPINGNGKQ | 2 | 3.98 | 3.76 | - | 3.89 | 3.35 | 3.96 | - | - |
|  | ALPGQLKPFETLLSQNQGGK | 3 | 4.91 | 4.94 | 4.60 | 4.61 | 4.92 | 4.93 | 4.85 | - |
|  | ASCLYGQLPK | 2 | 3.43 | 3.46 | 3.38 | 3.56 | 3.20 | 3.37 | 3.55 | - |
|  | DQQEAALVDMVNDGVEDLR | 3 | 6.84 | 6.64 | 6.54 | 6.33 | 6.54 | 6.84 | 4.26 | - |
|  | EEVVTVETWQEGSLK | 2 | 6.12 | 6.06 | 5.55 | 5.94 | 5.98 | 5.72 | - | - |
|  | FQDGDLTLYQSNTILR | 2 | 6.61 | 6.63 | 6.66 | 6.27 | 6.58 | 6.88 | 6.74 | - |
|  | MLLADQGQSWK | 2 | 3.85 | 4.13 | 3.86 | 4.09 | 3.57 | 3.26 | 2.51 | - |
|  | MLLADQGQSWKEEVVTVETWQEGSLK | 3 | 4.20 | - | - | 5.23 | - | - | 4.95 | - |
|  | MPPYTVVYFPVR | 2 | 4.32 | 4.24 | - | - | 3.81 | - | - | - |
|  | PFETLLSQNQGGK | 2 | 2.33 | 4.54 | 2.27 | - | 2.03 | - | - | - |
|  | TLGLYGK | 1 | 1.61 | - | 1.57 | 1.54 | 1.65 | - | - | - |
|  | YISLIYTNYEAGK | 2 | 5.27 | 4.87 | 4.99 | 4.59 | 4.83 | - | - | - |
|  | YISLIYTNYEAGKDDYVK | 3 | 5.82 | 6.03 | 6.04 | 5.96 | 6.08 | 2.77 | 2.84 | - |
|  |  |  | 14 | 12 | 11 | 12 | 13 | 9 | 7 |  |
| glutathione S-transferase theta, NP_000845.1, NP_001074312.1, NP_000844.2 | | | |  |  |  |  |  |  |  |
|  | C |  | - | 0.91 | - | - | - | 0.90 | 0.98 |  |
|  | AKDFPPADPTIK | 2 | - | - | - | - | - | - | 3.44 | - |
|  | ARVDEYLAWQHTTLR | 3 | - | - | - | - | - | - | 5.25 | - |
|  | AVYIFAK | 2 | - | - | - | - | - | - | 2.26 | - |
|  | EFLQINSLGK | 2 | - | 2.78 | - | - | - | - | - | - |
|  | GQHLSDAFAQVNPLK | 2 | - | - | - | - | - | 3.29 | 5.65 | - |
|  | GQHLSDAFAQVNPLKK | 2 | - | - | - | - | - | 4.38 | 5.62 | - |
|  | GTFGIPLWVQVLGPLIGVQVPEEK | 3 | - | 5.57 | - | - | - | - | - | - |
|  | IVDLIK | 1 | - | - | - | - | - | - | 1.68 | - |
|  | KNDIPFELR | 2 | - | - | - | - | - | 2.19 | 3.07 | - |
|  | LMPWVLAMIR | 2 | - | - | - | - | - | - | 3.31 | - |
|  | QRVEAAVGEDLFQEAHEVILK | 3 | - | - | - | - | - | - | 8.81 | - |
|  | TAMDQALQWLEDK | 2 | - | - | - | - | - | - | 4.80 | - |
|  | VDEYLAWQHTTLR | 2 | - | - | - | - | - | 2.98 | 4.60 | - |
|  | VEAAVGEDLFQEAHEVILK | 2 | - | - | - | - | - | 3.19 | 5.63 | - |
|  | VPDYWYPQDLQAR | 2 | - | - | - | - | - | - | 4.14 | - |
|  | YQTPDHWYPSDLQAR | 3 | - | 2.91 | - | - | - | - | - | - |
|  |  |  | 0 | 3 | 0 | 0 | 0 | 5 | 13 |  |
| glutathione S-transferase zeta, NP_665877.1, NP_665878.2, NP_001504.2 | | | |  |  |  |  |  |  |  |
|  | C |  | - | - | - | - | - | - | 0.98 |  |
|  | DFQALNPMK | 2 | - | - | - | - | - | - | 3.50 | - |
|  | DGGQQFSK | 2 | - | - | - | - | - | - | 2.89 | - |
|  | FKVDLTPYPTISSINK | 2 | - | - | - | - | - | - | 5.42 | - |
|  | FKVDLTPYPTISSINKR | 3 | - | - | - | - | - | - | 4.07 | - |
|  | GIDYETVPINLIK | 2 | - | - | - | - | - | - | 4.72 | - |
|  | IALALK | 1 | - | - | - | - | - | - | 1.86 | - |
|  | LLPQDPK | 1 | - | - | - | - | - | - | 2.06 | - |
|  | LLVLEAFQVSHPCR | 2 | - | - | - | - | - | - | 4.54 | - |
|  | MISDLIAGGIQPLQNLSVLK | 3 | - | - | - | - | - | - | 6.02 | - |
|  | VDLTPYPTISSINKR | 3 | - | - | - | - | - | - | 2.64 | - |
|  |  |  | 0 | 0 | 0 | 0 | 0 | 0 | 10 |  |
| glutathione synthetase, NP_000169.1 | |  |  |  |  |  |  |  |  |  |
|  | C |  | 0.90 | 0.90 | - | 0.92 | 0.90 | 0.95 | - |  |
|  | AIEHADGGVAAGVAVLDNPYPV | 2 | 2.74 | - | - | - | 2.21 | 2.65 | - | - |
|  | AIENELLAR | 2 | 3.04 | - | - | - | - | 2.55 | - | - |
|  | ALAEGVLLR | 2 | 3.16 | 3.23 | - | 3.50 | 3.05 | - | - | - |
|  | HVLSVLSK | 2 | 2.11 | - | - | - | - | 2.13 | - | - |
|  | LFVDGQEIAVVYFR | 2 | - | 2.32 | - | 2.77 | 2.34 | - | - | - |
|  | QIEINTISASFGGLASR | 0 | - | - | - | - | - | 4.84 | - | - |
|  | VVQCISELGIFGVYVR | 2 | 2.35 | - | - | - | 2.48 | - | - | - |
|  |  |  | 5 | 2 | 0 | 2 | 4 | 4 | 0 |  |
| glyceraldehyde-3-phosphate dehydrogenase, NP_002037.2 | |  |  |  |  |  |  |  |  |  |
|  | C |  | 0.98 | 0.97 | 0.98 | 0.98 | 0.98 | 0.98 | 0.98 |  |
|  | AGAHLQGGAK | 2 | 2.85 | 2.95 | 3.09 | 2.25 | 2.68 | 2.71 | 3.02 | - |
|  | GALQNIIPASTGAAK | 2 | 4.47 | 4.33 | 4.33 | 4.28 | 4.21 | 4.41 | 4.34 | - |
|  | IISNASCTTNCLAPLAK | 2 | 5.14 | 4.85 | 4.94 | 4.63 | 4.90 | 4.75 | 5.01 | - |
|  | LEKPAKYDDIK | 3 | - | - | - | - | - | 2.64 | - | - |
|  | LISWYDNEFGYSNR | 2 | 4.99 | 5.07 | 4.85 | 4.83 | 4.88 | 5.01 | 5.28 | - |
|  | LTGMAFR | 1 | - | 1.57 | - | - | - | - | - | - |
|  | LVINGNPITIFQER | 2 | 5.93 | 5.70 | 5.44 | 5.44 | 5.25 | 5.57 | 5.43 | - |
|  | LVINGNPITIFQERDPSK | 2 | 2.62 | 4.05 | - | - | - | 3.25 | 2.77 | - |
|  | QASEGPLK | 1 | 1.67 | - | - | - | - | 2.03 | 1.50 | - |
|  | RVIISAPSADAPMFVMGVNHEK | 3 | - | 6.17 | 6.17 | - | - | - | 5.46 | - |
|  | VDIVAINDPFIDLNYMVYMFQYDSTHGK | 3 | 4.99 | 5.09 | 5.38 | 4.80 | 4.95 | - | - | - |
|  | VGVNGFGR | 2 | 2.81 | 2.66 | 2.43 | 2.65 | 2.81 | 2.55 | 3.02 | - |
|  | VIHDNFGIVEGLMTTVHAITATQK | 3 | 7.98 | 7.93 | 7.69 | 8.20 | 7.58 | 6.20 | 7.44 | - |
|  | VIPELNGK | 1 | 1.91 | 1.84 | 1.79 | 1.90 | 1.68 | - | - | - |
|  | VPTANVSVVDLTCR | 2 | 4.65 | 4.69 | 4.50 | 4.75 | 4.43 | 4.56 | 4.99 | - |
|  | VVDLMAHMASK | 2 | 3.94 | 4.09 | 4.19 | 3.71 | 3.82 | 3.67 | 4.18 | - |
|  | VVDLMAHMASKE | 2 | - | - | - | - | - | 4.32 | 4.20 | - |
|  | WGDAGAEYVVESTGVFTTMEK | 2 | 6.47 | 6.44 | 6.63 | 6.30 | 6.48 | 7.04 | 7.32 | - |
|  | YDDIKK | 1 | - | 1.81 | - | - | - | - | - | - |
|  | YDNSLK | 1 | 2.03 | 2.11 | 2.01 | 1.91 | 1.97 | 2.02 | 2.13 | - |
|  |  |  | 15 | 17 | 14 | 13 | 13 | 15 | 15 |  |
| histamine N-methyltransferase, NP_001019246.1, NP_001019245.1, NP_008826.1 | | | |  |  |  |  |  |  |  |
|  | C |  | - | - | - | - | - | - | 0.94 |  |
|  | FFHSLLGTNAK | 3 | - | - | - | - | - | - | 3.20 | - |
|  | ILSIGGGAGEIDLQILSK | 2 | - | - | - | - | - | - | 2.34 | - |
|  | TSNLENVK | 2 | - | - | - | - | - | - | 2.78 | - |
|  | VQAQYPGVCINNEVVEPSAEQIAK | 2 | - | - | - | - | - | - | 4.82 | - |
|  |  |  | 0 | 0 | 0 | 0 | 0 | 0 | 4 |  |
| hydroxysteroid dehydrogenase like 2, NP_115679.2 | |  |  |  |  |  |  |  |  |  |
|  | C |  | - | - | - | - | - | - | 0.97 |  |
|  | ALPCIVDVR | 2 | - | - | - | - | - | - | 2.10 | - |
|  | KVDIIADAAYSIFQKPK | 3 | - | - | - | - | - | - | 2.79 | - |
|  | KVESTGAVPEFK | 2 | - | - | - | - | - | - | 2.51 | - |
|  | LAGCTVFITGASR | 2 | - | - | - | - | - | - | 3.90 | - |
|  | LLGTIYTAAEEIEAVGGK | 2 | - | - | - | - | - | - | 6.01 | - |
|  | TAIHTAAMDMLGGPGIESQCR | 3 | - | - | - | - | - | - | 4.47 | - |
|  | VAHILNISPPLNLNPVWFK | 3 | - | - | - | - | - | - | 4.07 | - |
|  |  |  | 0 | 0 | 0 | 0 | 0 | 0 | 7 |  |
| kynurenine 3-monooxygenase, NP_003670.2 | |  |  |  |  |  |  |  |  |  |
|  | M |  | - | - | - | - | - | - | 0.98 |  |
|  | AVDSLEQISNLISR | 3 | - | - | - | - | - | - | 4.88 | - |
|  | CNPEEGMITVLGSDKVPK | 2 | - | - | - | - | - | - | 3.36 | - |
|  | DVTCDLIVGCDGAYSTVR | 2 | - | - | - | - | - | - | 5.10 | - |
|  | LLTSNDVVDFFQK | 2 | - | - | - | - | - | - | 4.84 | - |
|  | SFTCTLFMPFEEFEK | 2 | - | - | - | - | - | - | 4.55 | - |
|  |  |  | 0 | 0 | 0 | 0 | 0 | 0 | 5 |  |
| liver carboxylesterase 1, NP_001020366.1, NP_001020365.1, NP_001257.4 | | | |  |  |  |  |  |  |  |
|  | M |  | - | - | - | - | - | 0.97 | 0.97 |  |
|  | AGQLLSELFTNR | 2 | - | - | - | - | - | 2.78 | 4.89 | - |
|  | AGQLLSELFTNRK | 3 | - | - | - | - | - | - | 4.37 | - |
|  | AISESGVALTSVLVK | 3 | - | - | - | - | - | 2.09 | 5.70 | - |
|  | AVEKPPQTEHIEL | 2 | - | - | - | - | - | - | 2.93 | - |
|  | DAGAPTYMYEFQYR | 2 | - | - | - | - | - | 2.49 | 4.57 | - |
|  | DAGAPTYMYEFQYRPSFSSDMKPK | 3 | - | - | - | - | - | - | 3.73 | - |
|  | DKEVAFWTNLFAK | 3 | - | - | - | - | - | - | 5.33 | - |
|  | DLFLDLIADVMFGVPSVIVAR | 3 | - | - | - | - | - | 6.09 | - | - |
|  | EGASEEEIR | 2 | - | - | - | - | - | - | 2.61 | - |
|  | EGYLQIGANTQAAQK | 2 | - | - | - | - | - | 2.17 | 5.56 | - |
|  | ELIPEATEK | 1 | - | - | - | - | - | - | 2.23 | - |
|  | ENIPLK | 1 | - | - | - | - | - | - | 1.89 | - |
|  | ENIPLKLSEDCLYLNIYTPADLTK | 3 | - | - | - | - | - | - | 6.43 | - |
|  | ESQPLLGTVIDGMLLLK | 3 | - | - | - | - | - | 4.67 | 5.75 | - |
|  | EVAFWTNLFAK | 2 | - | - | - | - | - | - | 4.30 | - |
|  | FLSLDLQGDPR | 2 | - | - | - | - | - | 2.60 | 4.04 | - |
|  | FTPPQPAEPWSFVK | 3 | - | - | - | - | - | 2.42 | 4.06 | - |
|  | FWANFAR | 2 | - | - | - | - | - | 2.35 | 2.98 | - |
|  | GDVKPLAEQIAITAGCK | 3 | - | - | - | - | - | - | 5.12 | - |
|  | GNWGHLDQVAALR | 2 | - | - | - | - | - | 2.81 | 4.59 | - |
|  | KAVEKPPQTEHIEL | 2 | - | - | - | - | - | - | 4.07 | - |
|  | KENIPLK | 2 | - | - | - | - | - | - | 2.74 | - |
|  | KGDVKPLAEQIAITAGCK | 3 | - | - | - | - | - | 3.04 | 6.38 | - |
|  | LGIWGFFSTGDEHSR | 2 | - | - | - | - | - | - | 4.73 | - |
|  | LKDKEVAFWTNLFAK | 3 | - | - | - | - | - | - | 4.89 | - |
|  | LSEDCLYLNIYTPADLTK | 2 | - | - | - | - | - | - | 6.51 | - |
|  | NFHTVPYMVGINK | 3 | - | - | - | - | - | 2.81 | 4.36 | - |
|  | NGNPNGEGLPHWPEYNQK | 2 | - | - | - | - | - | - | 4.98 | - |
|  | PSFSSDMKPK | 2 | - | - | - | - | - | - | 3.79 | - |
|  | QEFGWLIPMLMSYPLSEGQLDQK | 3 | - | - | - | - | - | - | 2.91 | - |
|  | QEFGWLIPMQLMSYPLSEGQLDQK | 3 | - | - | - | - | - | - | 5.75 | - |
|  | QKTEEELLETTLK | 3 | - | - | - | - | - | - | 4.01 | - |
|  | SYPLVCIAK | 2 | - | - | - | - | - | - | 2.54 | - |
|  | TAMSLLWK | 2 | - | - | - | - | - | - | 3.07 | - |
|  | TEEELLETTLK | 2 | - | - | - | - | - | - | 4.57 | - |
|  | TPEELQAER | 2 | - | - | - | - | - | 2.85 | 3.69 | - |
|  | TTTSAVMVHCLR | 2 | - | - | - | - | - | - | 3.44 | - |
|  | TVIGDHGDELFSVFGAPFLK | 2 | - | - | - | - | - | 3.46 | 6.08 | - |
|  | YLGGTDDTVK | 2 | - | - | - | - | - | - | 3.27 | - |
|  | YLGGTDDTVKK | 2 | - | - | - | - | - | - | 3.56 | - |
|  |  |  | 0 | 0 | 0 | 0 | 0 | 14 | 39 |  |
| long-chain-fatty-acid--CoA ligase 1, NP_004449.1, NP_055977.3, NP_001986.2 | | | |  |  |  |  |  |  |  |
|  | M |  | 0.82 | - | - | - | - | 0.95 | 0.98 |  |
|  | AELSLVFVDKPEK | 3 | - | - | - | - | - | - | 2.68 | - |
|  | AILEDMVR | 2 | - | - | - | - | - | - | 2.71 | - |
|  | ATENTVNPCPDDTLISFLPLAHMFER | 3 | - | - | - | - | - | - | 3.15 | - |
|  | CGVEVTSMK | 2 | - | - | - | - | - | - | 2.70 | - |
|  | DGWLHTGDIGK | 2 | - | - | - | - | - | - | 3.49 | - |
|  | DSGLKPFEQVK | 2 | - | - | - | - | - | - | 3.23 | - |
|  | GFEGSFEELCR | 2 | - | - | - | - | - | 3.89 | 4.19 | - |
|  | GIQVSNNGPCLGSR | 2 | - | - | - | - | - | - | 5.23 | - |
|  | GITLHPELFSIDNGLLTPTMK | 3 | - | - | - | - | - | - | 2.74 | - |
|  | GPNVFQGYLK | 2 | - | - | - | - | - | - | 3.78 | - |
|  | GPNVFQGYLKDPAK | 2 | - | - | - | - | - | 2.62 | 5.02 | - |
|  | IENIYMR | 2 | - | - | - | - | - | - | 2.42 | - |
|  | IFGQANTTLK | 2 | - | - | - | - | - | - | 3.35 | - |
|  | IFGQANTTLKR | 2 | - | - | - | - | - | - | 3.14 | - |
|  | IGFFQGDIR | 2 | - | - | - | - | - | 3.43 | 3.78 | - |
|  | IGYSSPLTLSDQSSK | 2 | - | - | - | - | - | - | 5.30 | - |
|  | IIVVMDAYGSELVER | 2 | - | - | - | - | - | - | 5.16 | - |
|  | KAILEDMVR | 2 | - | - | - | - | - | - | 3.39 | - |
|  | KPDQPYEWLSYK | 2 | - | - | - | - | - | - | 4.17 | - |
|  | LAQGEYIAPEK | 2 | 2.61 | - | - | - | - | 3.67 | 4.01 | - |
|  | LAQGEYIAPEKIENIYMR | 3 | - | - | - | - | - | - | 4.62 | - |
|  | LLLEGVENK | 2 | - | - | - | - | - | 2.23 | 3.60 | - |
|  | LLMDDLK | 2 | - | - | - | - | - | - | 2.62 | - |
|  | LMVTGAAPVSATVLTFLR | 2 | - | - | - | - | - | - | 4.81 | - |
|  | LTLLAQQK | 2 | - | - | - | - | - | - | 2.43 | - |
|  | LVDVEEMNYMAAEGEGEVCVK | 3 | - | - | - | - | - | - | 5.13 | - |
|  | MPELVDFR | 2 | - | - | - | - | - | 2.71 | 2.88 | - |
|  | NIVSDCSAFVK | 2 | - | - | - | - | - | 3.56 | 3.95 | - |
|  | NNSLWDR | 2 | - | - | - | - | - | - | 2.07 | - |
|  | PLKPPCDLSMQSVEVAGSGGAR | 3 | - | - | - | - | - | - | 4.55 | - |
|  | QVAELSECIGSALIQK | 2 | - | - | - | - | - | 3.45 | 6.17 | - |
|  | SQIDDLYSTIK | 2 | - | - | - | - | - | - | 3.77 | - |
|  | SQIDDLYSTIKV | 2 | - | - | - | - | - | 4.16 | 5.16 | - |
|  | TAEALDKDGWLHTGDIGK | 3 | - | - | - | - | - | - | 5.10 | - |
|  | TALLDISCVK | 2 | - | - | - | - | - | - | 3.28 | - |
|  | VGAPLICCEIK | 2 | - | - | - | - | - | - | 3.49 | - |
|  | VLQPTVFPVVPR | 2 | 2.87 | - | - | - | - | 3.82 | 4.09 | - |
|  | VVECVMLCHGAK | 2 | - | - | - | - | - | - | 4.26 | - |
|  | WLLDFASK | 2 | - | - | - | - | - | 2.26 | 2.70 | - |
|  |  |  | 2 | 0 | 0 | 0 | 0 | 11 | 39 |  |
| membrane primary amine oxidase, NP_003725.1 | |  |  |  |  |  |  |  |  |  |
|  | M |  | - | - | - | - | - | 0.94 | - |  |
|  | DAFCVFEQNQGLPLR | 2 | - | - | - | - | - | 4.34 | - | - |
|  | DAFCVFEQNQGLPLRR | 3 | - | - | - | - | - | 3.51 | - | - |
|  | LGPGLVDAAQAR | 2 | - | - | - | - | - | 4.30 | - | - |
|  | SPVPPGPAPPLQFYPQGPR | 3 | - | - | - | - | - | 5.30 | - | - |
|  | YQLAVTQR | 2 | - | - | - | - | - | 2.38 | - | - |
|  | YVDGGFGMGK | 2 | - | - | - | - | - | 2.84 | - | - |
|  |  |  | 0 | 0 | 0 | 0 | 0 | 6 | 0 |  |
| methylmalonate-semialdehyde dehydrogenase [acylating], NP_005580.1 | | | |  |  |  |  |  |  |  |
|  | C |  | - | - | - | - | - | - | 0.96 |  |
|  | AFPAWADTSVLSR | 2 | - | - | - | - | - | - | 4.00 | - |
|  | AISFVGSNK | 2 | - | - | - | - | - | - | 2.74 | - |
|  | DMDLYSYR | 2 | - | - | - | - | - | - | 2.67 | - |
|  | EGASILLDGR | 2 | - | - | - | - | - | - | 3.50 | - |
|  | ENTLNQLVGAAFGAAGQR | 2 | - | - | - | - | - | - | 5.91 | - |
|  | GDTNFYGK | 2 | - | - | - | - | - | - | 2.77 | - |
|  | LITLEQGK | 2 | - | - | - | - | - | - | 2.37 | - |
|  | QGIQFYTQLK | 2 | - | - | - | - | - | - | 3.22 | - |
|  | TLADAEGDVFR | 2 | - | - | - | - | - | - | 3.80 | - |
|  | VCNLIDSGTK | 2 | - | - | - | - | - | - | 3.67 | - |
|  | VNAGDQPGADLGPLITPQAK | 2 | - | - | - | - | - | - | 6.28 | - |
|  | VPGATMLLAK | 2 | - | - | - | - | - | - | 2.78 | - |
|  | WLPELVEHAK | 2 | - | - | - | - | - | - | 2.88 | - |
|  |  |  | 0 | 0 | 0 | 0 | 0 | 0 | 13 |  |
| microsomal glutathione S-transferase 1, NP_665734.1, NP_665735.1, NP_064696.1, NP_665707.1 | | | | |  |  |  |  |  |  |
|  | M |  | 0.97 | 0.98 | 0.49 | - | - | 0.90 | 0.97 |  |
|  | IYHTIAYLTPLPQPNR | 2 | 5.47 | 6.00 | 3.30 | - | - | 3.39 | 5.45 | - |
|  | KVFANPEDCVAFGK | 3 | 2.95 | 3.98 | - | - | - | 2.98 | 4.62 | - |
|  | KVFANPEDCVAFGKGENAK | 3 | - | - | - | - | - | - | 6.25 | - |
|  | MMLMSTATAFYR | 2 | - | - | - | - | - | - | 4.77 | - |
|  | VFANPEDCVAFGK | 2 | 3.31 | 3.57 | 2.34 | - | - | 3.84 | 4.70 | - |
|  |  |  | 3 | 3 | 2 | 0 | 0 | 3 | 5 |  |
| NAD(P)H dehydrogenase [quinone] 1, NP_000894.1, NP_001020605.1, NP_001020604.1 | | | | |  |  |  |  |  |  |
|  | C |  | 0.89 | 0.93 | - | - | 0.96 | - | - |  |
|  | EGHLSPDIVAEQK | 2 | 3.07 | 3.52 | - | - | 4.01 | - | - | - |
|  | FGLSVGHHLGK | 2 | - | 2.18 | - | - | - | - | - | - |
|  | KLEAADLVIFQFPLQWFGVPAILK | 3 | - | 5.58 | - | - | 5.68 | - | - | - |
|  | LKDPANFQYPAESVLAYK | 3 | 4.07 | 4.53 | - | - | 3.84 | - | - | - |
|  |  |  | 2 | 4 | 0 | 0 | 3 | 0 | 0 |  |
| NADH-ubiquinone oxidoreductase, NP_004997.4 | |  |  |  |  |  |  |  |  |  |
|  | M |  | 0.97 | 0.96 | 0.97 | - | - | 0.98 | 0.98 |  |
|  | ALSEIAGMTLPYDTLDQVR | 2 | - | - | - | - | - | 6.02 | 5.91 | - |
|  | AVTEGAQAVEEPSIC | 2 | - | - | - | - | - | 3.99 | 4.84 | - |
|  | DVAAIAGGLVDAEALVALK | 3 | 5.02 | 4.48 | 5.03 | - | - | - | 2.84 | - |
|  | FASEIAGVDDLGTTGR | 2 | - | - | - | - | - | 6.16 | 6.00 | - |
|  | FEAPLFNAR | 2 | - | - | - | - | - | 2.72 | 3.34 | - |
|  | GLLTYTSWEDALSR | 2 | - | - | - | - | - | 4.56 | 5.20 | - |
|  | GNDMQVGTYIEK | 2 | - | - | - | - | - | - | 4.46 | - |
|  | GWNILTNSEK | 2 | - | - | - | - | - | 2.87 | 3.01 | - |
|  | IASQVAALDLGYKPGVEAIR | 3 | 2.84 | - | - | - | - | 3.65 | - | - |
|  | ILQDIASGSHPFSQVLK | 3 | 3.37 | - | - | - | - | 3.56 | 2.85 | - |
|  | KPMVVLGSSALQR | 3 | - | - | - | - | - | - | 4.16 | - |
|  | LEEVSPNLVR | 2 | - | - | - | - | - | - | 3.40 | - |
|  | LSVAGNCR | 2 | - | - | - | - | - | - | 2.42 | - |
|  | LTEPMVR | 2 | - | - | - | - | - | - | 2.10 | - |
|  | LVNQQLLADPLVPPQLTIK | 3 | - | - | - | - | - | 3.80 | 3.91 | - |
|  | MCLVEIEK | 2 | - | - | - | - | - | - | 2.56 | - |
|  | MTSGVTGDWK | 2 | - | - | - | - | - | - | 3.16 | - |
|  | NDGAAILAAVSSIAQK | 2 | - | - | - | - | - | 5.04 | 5.44 | - |
|  | NRLEEVSPNLVR | 3 | - | - | - | - | - | 3.13 | 3.23 | - |
|  | SATYVNTEGR | 2 | - | - | - | - | - | 3.35 | 3.73 | - |
|  | VAGMLQSFQGK | 2 | - | - | - | - | - | 4.02 | 4.30 | - |
|  | VALIGSPVDLTYTYDHLGDSPK | 3 | 3.31 | 3.08 | 2.57 | - | - | 3.10 | 3.55 | - |
|  | VAVTPPGLAR | 2 | 2.03 | - | - | - | - | 2.59 | 2.73 | - |
|  | VDSDTLCTEEVFPTAGAGTDLR | 3 | - | - | - | - | - | 4.96 | - | - |
|  | VLFLLGADGGCITR | 2 | - | - | 2.92 | - | - | 4.21 | 4.88 | - |
|  | VVAACAMPVMK | 2 | - | - | - | - | - | - | 3.82 | - |
|  | YDDIEGANYFQQANELSK | 2 | - | - | - | - | - | 3.73 | 3.88 | - |
|  |  |  | 5 | 2 | 3 | 0 | 0 | 18 | 25 |  |
| NADPH--cytochrome P450 reductase, NP_000932.3 | |  |  |  |  |  |  |  |  |  |
|  | M |  | 0.95 | 0.93 | 0.95 | - | - | - | 0.99 |  |
|  | DGALTQLNVAFSR | 2 | 3.17 | - | - | - | - | - | 4.43 | - |
|  | EEVPEFTK | 2 | - | - | - | - | - | - | 2.36 | - |
|  | EVGETLLYYGCR | 2 | - | - | - | - | - | - | 4.32 | - |
|  | FAVFGLGNK | 2 | - | - | - | - | - | - | 3.04 | - |
|  | GVATNWLR | 2 | - | - | - | - | - | - | 3.03 | - |
|  | HPFPCPTSYR | 2 | - | - | - | - | - | - | 2.15 | - |
|  | IQTLTSSVR | 2 | 2.55 | 2.19 | 2.28 | - | - | - | 3.11 | - |
|  | KEEVPEFTK | 2 | - | - | - | - | - | - | 3.02 | - |
|  | KKEEVPEFTK | 3 | - | - | - | - | - | - | 2.95 | - |
|  | LEQLGAQR | 2 | 2.23 | 2.21 | - | - | - | - | 2.73 | - |
|  | LKSYENQKPPFDAK | 3 | 2.14 | - | - | - | - | - | 3.35 | - |
|  | NIIVFYGSQTGTAEEFANR | 2 | - | 2.23 | 3.68 | - | - | - | 7.35 | - |
|  | NPFLAAVTTNR | 2 | 3.78 | 3.45 | 3.39 | - | - | - | 4.31 | - |
|  | NPFLAAVTTNRK | 2 | - | - | - | - | - | - | 3.97 | - |
|  | RLEQLGAQR | 2 | - | - | - | - | - | - | 3.76 | - |
|  | RSDEDYLYR | 2 | - | - | - | - | - | - | 3.08 | - |
|  | SDEDYLYR | 2 | - | - | - | - | - | - | 3.26 | - |
|  | SYENQKPPFDAK | 2 | 2.54 | - | - | - | - | - | 4.21 | - |
|  | TALTYYLDITNPPR | 3 | 3.44 | 3.13 | 3.70 | - | - | - | 5.45 | - |
|  | TNVLYELAQYASEPSEQELLR | 3 | 4.70 | 5.04 | 4.33 | - | - | - | 6.16 | - |
|  | TNVLYELAQYASEPSEQELLRK | 3 | - | - | - | - | - | - | 5.80 | - |
|  | TYEHFNAMGK | 2 | - | - | - | - | - | - | 3.20 | - |
|  | VYMGEMGR | 2 | - | - | - | - | - | - | 2.75 | - |
|  | VYVQHLLK | 2 | - | 2.23 | - | - | - | - | 3.17 | - |
|  | YESGDHVAVYPANDSALVNQLGK | 3 | 5.47 | 4.57 | 4.23 | - | - | - | 6.15 | - |
|  | YSLDVWS | 1 | - | - | - | - | - | - | 1.66 | - |
|  | YYSIASSSK | 2 | - | - | - | - | - | - | 2.69 | - |
|  |  |  | 9 | 8 | 6 | 0 | 0 | 0 | 27 |  |
| nicotinamide N-methyltransferase, NP_006160.1 | |  |  |  |  |  |  |  |  |  |
|  | C |  | - | - | - | - | - | - | 0.98 |  |
|  | DTYLSHFNPR | 2 | - | - | - | - | - | - | 3.72 | - |
|  | EAVEAAVK | 1 | - | - | - | - | - | - | 2.33 | - |
|  | EIVVTDYSDQNLQELEK | 2 | - | - | - | - | - | - | 6.62 | - |
|  | EPEAFDWSPVVTYVCDLEGNR | 3 | - | - | - | - | - | - | 5.28 | - |
|  | FSSLPLGR | 2 | - | - | - | - | - | - | 2.04 | - |
|  | GDLLIDIGSGPTIYQLLSACESFK | 3 | - | - | - | - | - | - | 6.88 | - |
|  | HSAESQILK | 2 | - | - | - | - | - | - | 3.41 | - |
|  | IFCLDGVK | 2 | - | - | - | - | - | - | 3.00 | - |
|  | KEPEAFDWSPVVTYVCDLEGNR | 3 | - | - | - | - | - | - | 6.70 | - |
|  | NLGSLLKPGGFLVIMDALK | 2 | - | - | - | - | - | - | 4.53 | - |
|  | SSYYMIGEQK | 2 | - | - | - | - | - | - | 4.06 | - |
|  | VKGPEKEEK | 2 | - | - | - | - | - | - | 2.10 | - |
|  |  |  | 0 | 0 | 0 | 0 | 0 | 0 | 12 |  |
| peroxiredoxin-1, NP_002565.1, NP_859047.1, NP_859048.1 | |  |  |  |  |  |  |  |  |  |
|  | C |  | 0.96 | 0.96 | 0.96 | 0.94 | 0.96 | 0.93 | 0.96 |  |
|  | ADEGISFR | 2 | 3.06 | 3.09 | 3.00 | 2.35 | 2.91 | 2.92 | 3.17 | - |
|  | ATAVMPDGQFK | 2 | 2.83 | 3.24 | 3.02 | - | 3.00 | 2.78 | 3.11 | - |
|  | DISLSDYK | 2 | 2.99 | 2.72 | 2.75 | 2.83 | 2.79 | 2.65 | 2.79 | - |
|  | GLFIIDDK | 2 | 3.09 | 3.09 | 3.06 | 2.95 | 2.98 | 3.00 | 3.03 | NP_006397.1 |
|  | HGEVCPAGWK | 2 | - | 2.04 | - | - | - | - | - | NP_006397.1, NP_005800.3 |
|  | HGEVCPAGWKPGSDTIKPDVQK | 3 | 5.69 | 5.59 | 6.07 | 5.58 | 5.71 | - | 6.44 | - |
|  | IGHPAPNFK | 2 | 2.70 | 2.50 | 2.71 | 2.49 | 2.39 | 2.44 | 2.73 | - |
|  | LNCQVIGASVDSHFCHLAWVNTPK | 3 | 3.92 | 4.53 | - | - | 4.43 | - | 4.71 | - |
|  | LVQAFQFTDK | 2 | 3.97 | 3.88 | 3.57 | 3.70 | 3.85 | 3.86 | 3.95 | - |
|  | PGSDTIKPDVQK | 3 | 3.86 | 3.99 | 3.66 | 3.77 | 4.23 | - | 2.77 | - |
|  | QGGLGPMNIPLVSDPK | 2 | 3.65 | 3.69 | 3.21 | 3.22 | 3.76 | 3.12 | 4.38 | - |
|  | QGGLGPMNIPLVSDPKR | 3 | - | - | - | - | - | - | 3.88 | - |
|  | QITVNDLPVGR | 2 | 3.48 | 3.50 | 3.25 | 3.26 | 3.31 | 3.26 | 3.49 | NP_005800.3 |
|  | RTIAQDYGVLK | 2 | 2.80 | - | - | - | 2.91 | - | 3.32 | - |
|  | SKEYFSK | 2 | 2.15 | - | - | - | - | - | 2.26 | NP_005800.3 |
|  | SVDETLR | 2 | 2.41 | 2.24 | 2.15 | - | 2.18 | 2.03 | 2.17 | NP_006397.1 |
|  | TIAQDYGVLK | 2 | 3.03 | 2.85 | 2.03 | 2.04 | 3.39 | 3.13 | 3.42 | - |
|  |  |  | 15 | 14 | 12 | 10 | 14 | 10 | 16 |  |
| peroxiredoxin-2, NP_005800.3, NP_859428.1 | |  |  |  |  |  |  |  |  |  |
|  | C |  | 0.98 | 0.98 | 0.97 | 0.98 | 0.98 | 0.91 | 0.97 |  |
|  | ATAVVDGAFK | 2 | 3.40 | 3.20 | 3.23 | 3.25 | 2.89 | 3.22 | 3.21 | - |
|  | EGGLGPLNIPLLADVTR | 2 | 5.15 | 5.25 | 4.83 | 5.50 | 4.97 | 4.48 | 4.87 | - |
|  | GLFIIDGK | 2 | 2.49 | 2.53 | 2.28 | 2.32 | 2.21 | 2.11 | 2.49 | - |
|  | GLFIIDGKGVLR | 2 | - | - | - | - | - | - | 2.38 | - |
|  | HGEVCPAGWK | 2 | - | 2.04 | - | - | - | - | - | NP_006397.1, NP_002565.1, NP_859047.1, NP_859048.1 |
|  | IGKPAPDFK | 2 | 2.29 | 2.15 | 2.10 | 2.01 | 2.02 | 2.22 | 2.22 | - |
|  | KEGGLGPLNIPLLADVTR | 2 | 6.26 | 5.97 | 5.92 | 5.82 | 6.06 | 3.44 | 6.10 | - |
|  | KLGCEVLGVSVDSQFTHLAWINTPR | 3 | - | 5.84 | - | 2.98 | - | - | - | - |
|  | LGCEVLGVSVDSQFTHLAWINTPR | 3 | 5.18 | 5.08 | 4.67 | 2.30 | 4.10 | 4.75 | - | - |
|  | LSEDYGVLK | 2 | 3.21 | 3.10 | 3.31 | 2.99 | 3.11 | 2.51 | 2.91 | - |
|  | QITVNDLPVGR | 2 | 3.48 | 3.50 | 3.52 | 3.26 | 3.31 | 3.13 | 3.49 | NP_002565.1, NP_859047.1, NP_859048.1 |
|  | RLSEDYGVLK | 2 | 2.77 | 3.09 | - | 2.53 | - | 3.24 | 3.52 | - |
|  | SKEYFSK | 2 | 2.15 | - | - | - | - | - | 2.26 | NP_002565.1, NP_859047.1, NP_859048.1 |
|  | SVDEALR | 1 | - | - | - | - | - | - | 1.80 | - |
|  | TDEGIAYR | 2 | 3.20 | 3.16 | 3.04 | 3.15 | 3.17 | 2.88 | 3.05 | - |
|  |  |  | 11 | 12 | 9 | 11 | 9 | 10 | 12 |  |
| peroxiredoxin-4, NP_006397.1 | |  |  |  |  |  |  |  |  |  |
|  | C |  | 0.85 | 0.85 | 0.85 | - | 0.94 | 0.96 | 0.98 |  |
|  | DYGVYLEDSGHTLR | 2 | - | - | - | - | 3.99 | 4.22 | - | - |
|  | GKYLVFFFYPLDFTFVCPTEIIAFGDR | 3 | - | - | - | - | 3.31 | - | - | - |
|  | GLFIIDDK | 2 | 3.09 | 3.09 | 3.06 | - | 2.98 | 2.97 | 3.03 | NP_002565.1, NP_859047.1, NP_859048.1 |
|  | HGEVCPAGWK | 2 | - | 2.04 | - | - | - | - | - | NP_002565.1, NP_859047.1, NP_859048.1, NP_005800.3 |
|  | HGEVCPAGWKPGSETIIPDPAGK | 3 | - | - | - | - | - | 2.99 | - | - |
|  | ISKPAPYWEGTAVIDGEFK | 3 | - | - | - | - | - | - | 4.18 | - |
|  | QITLNDLPVGR | 2 | - | - | - | - | - | - | 3.09 | - |
|  | SINTEVVACSVDSQFTHLAWINTPR | 3 | - | - | - | - | - | 5.56 | - | - |
|  | SVDETLR | 2 | 2.41 | 2.24 | 2.15 | - | 2.18 | 2.03 | 2.17 | NP_002565.1, NP_859047.1, NP_859048.1 |
|  | TREEECHFYAGGQVYPGEASR | 3 | - | - | - | - | - | - | 6.98 | - |
|  | VSVADHSLHLSK | 2 | - | - | - | - | - | - | 2.73 | - |
|  |  |  | 2 | 3 | 2 | 0 | 4 | 5 | 6 |  |
| peroxiredoxin-5, NP_857635.1, NP_857634.1, NP_036226.1 | |  |  |  |  |  |  |  |  |  |
|  | C |  | 0.97 | 0.97 | 0.94 | 0.97 | 0.97 | 0.97 | 0.97 |  |
|  | ALNVEPDGTGLTCSLAPNIISQL | 2 | 4.63 | 3.37 | - | 4.37 | 4.53 | 4.39 | 4.88 | - |
|  | ETDLLLDDSLVSIFGNR | 2 | 5.15 | 4.94 | - | 5.18 | 5.08 | 5.02 | 5.23 | - |
|  | FSMVVQDGIVK | 2 | - | - | - | - | 4.02 | - | - | - |
|  | GVLFGVPGAFTPGCSK | 2 | 3.99 | 3.96 | - | 3.53 | 4.40 | 3.54 | 4.17 | - |
|  | LLADPTGAFGK | 2 | 2.44 | - | 2.38 | 3.09 | 3.46 | 2.39 | 3.07 | - |
|  | THLPGFVEQAEALK | 3 | 4.42 | 3.85 | - | 3.95 | 4.11 | - | 3.84 | - |
|  | VGDAIPAVEVFEGEPGNK | 2 | 5.43 | 5.49 | 5.48 | 5.46 | 5.28 | - | - | - |
|  | VGDAIPAVEVFEGEPGNKVNLAELFK | 3 | - | 2.85 | 2.52 | 3.14 | 2.71 | 2.82 | 3.31 | - |
|  | VNLAELFK | 2 | 2.98 | 2.83 | 2.91 | 2.94 | 2.89 | 2.50 | 2.95 | - |
|  |  |  | 7 | 7 | 4 | 8 | 9 | 6 | 7 |  |
| peroxiredoxin-6, NP_004896.1 | |  |  |  |  |  |  |  |  |  |
|  | C |  | 0.97 | 0.97 | 0.97 | 0.98 | 0.98 | 0.97 | 0.98 |  |
|  | DFTPVCTTELGR | 2 | 3.59 | 3.72 | 3.17 | 3.48 | 2.65 | 3.56 | 3.62 | - |
|  | DGDSVMVLPTIPEEEAK | 2 | 3.76 | - | - | - | - | - | 4.19 | - |
|  | DINAYNCEEPTEK | 2 | 4.50 | 4.53 | 4.38 | 4.01 | - | 3.88 | 4.63 | - |
|  | DINAYNCEEPTEKLPFPIIDDR | 3 | - | - | - | - | - | 3.84 | 6.35 | - |
|  | ELAILLGMLDPAEK | 2 | 4.44 | - | - | - | - | 3.47 | 4.66 | - |
|  | ELAILLGMLDPAEKDEK | 2 | 2.94 | - | - | - | - | - | 5.61 | - |
|  | FHDFLGDSWGILFSHPR | 3 | 5.42 | 4.89 | 4.88 | 5.07 | 4.87 | - | - | - |
|  | LIALSIDSVEDHLAWSK | 2 | 5.56 | 5.50 | 5.09 | 5.22 | 5.70 | 5.08 | 5.87 | - |
|  | LPFPIIDDR | 2 | 3.40 | 3.44 | 3.33 | 3.46 | 2.02 | 3.08 | 3.46 | - |
|  | LSILYPATTGR | 2 | 3.21 | 3.53 | - | 3.01 | - | 2.13 | 3.41 | - |
|  | NFDEILR | 2 | 2.65 | 2.75 | 2.45 | 2.50 | - | 2.32 | 2.81 | - |
|  | RVATPVDWK | 2 | - | - | - | - | - | - | 2.03 | - |
|  | VATPVDWK | 1 | 2.13 | 2.09 | 1.96 | 1.96 | - | 2.12 | 2.04 | - |
|  | VVFVFGPDK | 2 | 3.27 | 3.11 | 3.28 | 3.02 | - | 3.13 | 3.23 | - |
|  | VVFVFGPDKK | 2 | 3.14 | 3.32 | 3.20 | - | - | 2.90 | 3.53 | - |
|  | VVISLQLTAEK | 2 | 3.66 | 3.57 | 3.07 | 3.22 | 3.71 | - | - | - |
|  |  |  | 14 | 11 | 10 | 10 | 5 | 11 | 14 |  |
| prostacyclin synthase, NP_000952.1 | |  |  |  |  |  |  |  |  |  |
|  | M |  | - | - | - | - | - | 0.96 | - |  |
|  | DPEIYTDPEVFK | 2 | - | - | - | - | - | 3.75 | - | - |
|  | IFDVQLPHYSPSDEK | 3 | - | - | - | - | - | 2.94 | - | - |
|  | LLLFPFLSPQR | 2 | - | - | - | - | - | 2.84 | - | - |
|  | LTAAPFITR | 2 | - | - | - | - | - | 2.18 | - | - |
|  | VLDSTPVLDSVLSESLR | 2 | - | - | - | - | - | 5.35 | - | - |
|  |  |  | 0 | 0 | 0 | 0 | 0 | 5 | 0 |  |
| quinone oxidoreductase PIG3, NP_004872.2, NP_671713.1 | |  |  |  |  |  |  |  |  |  |
|  | C |  | 0.97 | 0.97 | - | 0.97 | - | - | - |  |
|  | EVAKPSPGEGEVLLK | 3 | 3.01 | 3.05 | - | 2.85 | - | - | - | - |
|  | GAGVNLILDCIGGSYWEK | 2 | 5.47 | 5.60 | - | 5.52 | - | - | - | - |
|  | LGAAAGFNYK | 2 | 3.12 | 2.92 | - | 3.04 | - | - | - | - |
|  | VAASALNR | 2 | - | - | - | 2.04 | - | - | - | - |
|  |  |  | 3 | 3 | 0 | 4 | 0 | 0 | 0 |  |
| short-chain dehydrogenase/reductase 7, NP_057113.1, NP_001099041.1, NP_056325.2 | | | | |  |  |  |  |  |  |
|  | M |  | - | - | 0.95 | - | - | 0.96 | 0.96 |  |
|  | ADGDLTLLWAEWQGR | 2 | - | - | - | - | - | - | 4.07 | - |
|  | AVLQEFGR | 2 | - | - | - | - | - | 2.67 | 2.72 | - |
|  | DILVLPLDLTDTGSHEAATK | 2 | - | - | - | - | - | 5.80 | 5.78 | - |
|  | EKDILVLPLDLTDTGSHEAATK | 3 | - | - | - | - | - | 3.69 | 5.48 | - |
|  | IVTVNSILGIISVPLSIGYCASK | 3 | - | - | 4.84 | - | - | 4.22 | - | - |
|  | LIELNYLGTVSLTK | 2 | - | - | 3.81 | - | - | 2.90 | - | - |
|  | LMLISMANDLK | 2 | - | - | - | - | - | - | 4.21 | - |
|  | SGVDADSSYFK | 2 | - | - | - | - | - | 2.10 | 4.25 | - |
|  |  |  | 0 | 0 | 2 | 0 | 0 | 6 | 6 |  |
| succinate-semialdehyde dehydrogenase, NP_733936.1, NP_001071.1 | | | |  |  |  |  |  |  |  |
|  | C |  | - | - | - | - | - | - | 0.97 |  |
|  | EVGEAICTDPLVSK | 2 | - | - | - | - | - | - | 4.28 | - |
|  | IITAESGKPLK | 2 | - | - | - | - | - | - | 3.20 | - |
|  | ISFTGSTTTGK | 2 | - | - | - | - | - | - | 3.30 | - |
|  | LAGLSAALLR | 2 | - | - | - | - | - | - | 3.38 | - |
|  | NTGQTCVCSNQFLVQR | 2 | - | - | - | - | - | - | 5.04 | - |
|  | VGNGFEEGTTQGPLINEK | 2 | - | - | - | - | - | - | 4.67 | - |
|  | YGIDEYLELK | 2 | - | - | - | - | - | - | 3.63 | - |
|  |  |  | 0 | 0 | 0 | 0 | 0 | 0 | 7 |  |
| sulfide:quinone oxidoreductase, NP_067022.1 | |  |  |  |  |  |  |  |  |  |
|  | M |  | 0.78 | 0.85 | 0.87 | 0.78 | - | 0.68 | 0.98 |  |
|  | AEPLETFPFDQSK | 2 | 3.03 | 3.66 | 3.45 | 3.33 | - | 2.82 | 4.28 | - |
|  | ALQDFKEGNAIFTFPNTPVK | 3 | - | - | - | - | - | - | 4.69 | - |
|  | ANIIFNTSLGAIFGVK | 2 | 3.07 | - | - | - | - | - | - | - |
|  | EGNAIFTFPNTPVK | 2 | 3.37 | 3.63 | 2.75 | 2.71 | - | 2.90 | 4.21 | - |
|  | GYWGGPAFLR | 2 | - | - | - | - | - | - | 3.16 | - |
|  | IGSNYSVK | 2 | - | - | - | - | - | - | 2.61 | - |
|  | IMYLSEAYFR | 2 | - | - | - | - | - | - | 3.86 | - |
|  | KVGAENVAIVEPSER | 3 | - | 3.18 | - | - | - | - | 3.66 | - |
|  | KYADALQEIIQER | 3 | - | - | - | - | - | - | 3.58 | - |
|  | PTASVIPSGVEWIK | 2 | - | - | - | - | - | - | 3.94 | - |
|  | QLSSSGRPTASVIPSGVEWIK | 3 | - | - | - | - | - | - | 3.92 | - |
|  | RYPNVFGIGDCTNLPTSK | 3 | - | - | - | - | - | - | 5.09 | - |
|  | TAAAVAAQSGILDR | 2 | - | 2.07 | - | - | - | - | 5.21 | - |
|  | TSPVADAAGWVDVDK | 2 | - | - | - | - | - | - | 2.51 | - |
|  | TSPVADAAGWVDVDKETLQHR | 3 | - | - | - | - | - | - | 3.60 | - |
|  | VGAENVAIVEPSER | 2 | - | - | - | - | - | - | 4.18 | - |
|  | YADALQEIIQER | 2 | - | - | - | - | - | - | 4.44 | - |
|  | YPNVFGIGDCTNLPTSK | 2 | - | - | - | - | - | - | 5.27 | - |
|  |  |  | 3 | 4 | 2 | 2 | 0 | 2 | 17 |  |
| sulfotransferase 1A, NP_001045.1, NP_803878.1, NP_803880.1, NP_001046.2, NP_003157.1, NP_001017390.1, NP_001017389.1, NP_808220.1, NP_803564.1, NP_803566.1, NP_803565.1 | | | | | | | | | | |
|  | C |  | - | - | - | - | - | - | 0.95 |  |
|  | APGIPSGMETLK | 2 | - | - | - | - | - | - | 2.50 | - |
|  | FDADYAEK | 2 | - | - | - | - | - | - | 3.00 | - |
|  | ILEFVGR | 2 | - | - | - | - | - | - | 2.45 | - |
|  | MAGCSLSFR | 2 | - | - | - | - | - | - | 3.32 | - |
|  | NPMTNYTTVPQEFMDHSISPFMR | 3 | - | - | - | - | - | - | 3.58 | - |
|  | PDDLLISTYPK | 2 | - | - | - | - | - | - | 2.63 | - |
|  | THLPLALLPQTLLDQK | 2 | - | - | - | - | - | - | 5.25 | - |
|  | TTFTVAQNER | 2 | - | - | - | - | - | - | 3.67 | - |
|  | VHPEPGTWDSFLEK | 2 | - | - | - | - | - | - | 3.12 | - |
|  | VPFLEFK | 2 | - | - | - | - | - | - | 2.64 | - |
|  | YFAEALGPLQSFQARPDDLLISTYPK | 3 | - | - | - | - | - | - | 5.33 | - |
|  |  |  | 0 | 0 | 0 | 0 | 0 | 0 | 11 |  |
| sulfotransferase 2B1, NP_004596.2, NP_814444.1 | |  |  |  |  |  |  |  |  |  |
|  | C |  | 0.98 | 0.98 | 0.98 | 0.98 | - | - | - |  |
|  | APWCETIVGAFSLPDQYSPR | 3 | - | - | - | 3.90 | - | - | - | - |
|  | DDDIFIITYPK | 2 | 3.46 | 3.87 | 3.74 | 4.30 | - | - | - | - |
|  | DNFLFITYEELQQDLQGSVER | 3 | 6.02 | 6.16 | 6.27 | 6.13 | - | - | - | - |
|  | DPGTPDQFLR | 2 | - | 2.82 | 3.11 | 3.05 | - | - | - | - |
|  | DVVVSLYHYSK | 2 | - | - | - | 3.52 | - | - | - | - |
|  | EPRPNSSPSPSPGQASETPHPRPS | 3 | - | 4.20 | - | - | - | - | - | - |
|  | GKDNFLFITYEELQQDLQGSVER | 3 | 5.26 | 5.80 | 6.08 | 5.51 | - | - | - | - |
|  | GVPFPVGLYSLESISLAENTQDVR | 2 | 3.18 | 4.94 | 5.46 | 5.37 | - | - | - | - |
|  | LPGEYFR | 2 | - | 2.03 | - | 2.09 | - | - | - | - |
|  | NHFTVAQSEAFDR | 3 | - | 3.45 | 3.62 | 3.98 | - | - | - | - |
|  | PNSSPSPSPGQASETPHPRPS | 2 | 3.22 | - | - | - | - | - | - | - |
|  | PSLEPNTSLER | 2 | - | - | - | 2.97 | - | - | - | - |
|  |  |  | 5 | 8 | 6 | 10 | 0 | 0 | 0 |  |
| thiosulfate sulfurtransferase, NP_003303.2 | |  |  |  |  |  |  |  |  |  |
|  | C |  | 0.92 | - | - | 0.96 | - | - | 0.97 |  |
|  | DTASPYEMMLPSEAGFAEYVGR | 2 | - | - | - | - | - | - | 5.62 | - |
|  | EGHPVTSEPSRPEPAVFK | 2 | - | - | - | - | - | - | 5.12 | - |
|  | FLGTEPEPDAVGLDSGHIR | 3 | 3.29 | - | - | 3.89 | - | - | 5.28 | - |
|  | FQLVDSR | 2 | - | - | - | - | - | - | 2.35 | - |
|  | GAVNMPFMDFLTEDGFEK | 2 | - | - | - | - | - | - | 5.77 | - |
|  | HVPGASFFDIEECR | 3 | - | - | - | - | - | - | 4.99 | - |
|  | KVDLSQPLIATCR | 2 | - | - | - | - | - | - | 4.92 | - |
|  | LGPGLR | 1 | - | - | - | - | - | - | 1.67 | - |
|  | TVSVLNGGFR | 2 | - | - | - | - | - | - | 2.27 | - |
|  | TYEQVLENLESK | 2 | 3.82 | - | - | 4.63 | - | - | 5.30 | - |
|  | VDLSQPLIATCR | 2 | - | - | - | - | - | - | 4.43 | - |
|  | VLDASWYSPGTR | 2 | - | - | - | - | - | - | 3.98 | - |
|  | VWWMFR | 2 | - | - | - | - | - | - | 2.18 | - |
|  |  |  | 2 | 0 | 0 | 2 | 0 | 0 | 13 |  |
| xanthine dehydrogenase/oxidase, NP_000370.2 | |  |  |  |  |  |  |  |  |  |
|  | C |  | - | - | - | - | - | - | 0.96 |  |
|  | DPPADVQLFQEVPK | 2 | - | - | - | - | - | - | 4.33 | - |
|  | ITYEELPAIITIEDAIK | 2 | - | - | - | - | - | - | 5.16 | - |
|  | KVPGFVCFISADDVPGSNITGICNDETVFAK | 3 | - | - | - | - | - | - | 6.92 | - |
|  | LDPTFASATLLFQK | 2 | - | - | - | - | - | - | 4.65 | - |
|  | LDPTFASATLLFQKDPPADVQLFQEVPK | 3 | - | - | - | - | - | - | 2.97 | - |
|  | LDSPATPEK | 2 | - | - | - | - | - | - | 2.88 | - |
|  | LGQENLEDK | 2 | - | - | - | - | - | - | 3.13 | - |
|  | NADPETTLLAYLR | 2 | - | - | - | - | - | - | 4.05 | - |
|  | SIDTSEAK | 2 | - | - | - | - | - | - | 2.07 | - |
|  | TNLPSNTAFR | 2 | - | - | - | - | - | - | 2.58 | NP_001150.3 |
|  | VLFKPGTTEVQELALCYGGMANR | 3 | - | - | - | - | - | - | 3.21 | - |
|  |  |  | 0 | 0 | 0 | 0 | 0 | 0 | 11 |  |
|  |  |  |  |  |  |  |  |  |  |  |
